# Isoorotamide-based peptide nucleic acid nucleobases with extended linkers aimed at distal base recognition of adenosine in double helical RNA

**DOI:** 10.3762/bjoc.21.193

**Published:** 2025-11-12

**Authors:** Grant D Walby, Brandon R Tessier, Tristan L Mabee, Jennah M Hoke, Hallie M Bleam, Angelina Giglio-Tos, Emily E Harding, Vladislavs Baskevics, Martins Katkevics, Eriks Rozners, James A MacKay

**Affiliations:** 1 Department of Chemistry and Biochemistry, Elizabethtown College, 1 Alpha Drive, Elizabethtown, PA 17022, USA; 2 Department of Chemistry, Ohio Wesleyan University, Delaware, OH, 43015, USA; 3 Department of Chemistry, Binghamton University, 4400 Vestal Parkway East, Binghamton, NY 13902, USA; 4 Latvian Institute of Organic Synthesis, Aizkraukles 21, Riga, LV-1006, Latvia

**Keywords:** Hoogsteen hydrogen bonding, modified nucleobases, peptide nucleic acids, PNA–RNA triplexes, RNA recognition

## Abstract

Non-coding ribonucleic acid (RNA) impacts many biological processes; however, the complexities of its many roles are not completely understood. Therefore, designing tools for molecular recognition is of paramount importance. Peptide nucleic acids (PNA) show promise as a tool for selective recognition of double helical regions of RNA. We herein report the synthesis and binding studies of new isoorotamide-based PNA monomers that target uridine–adenosine base pairs via a distal base recognition strategy. Monomers were designed with an arylisoorotamide core attached to a linker aimed at bypassing the uridine in a U–A pair and ultimately forming Hoogsteen hydrogen bonds with adenosine. Three new monomers were prepared and incorporated into PNAs that were screened against matched RNA hairpins using UV thermal melting and isothermal titration calorimetry experiments. Two of the three PNA oligonucleotides that contained distal binding monomers (**Db**) demonstrated slightly higher affinity for A–U base pairs while one demonstrated slightly higher affinity for the G–C base pair. These results provide insight into the nature of PNA monomer design particularly around linker design and rigidity.

## Introduction

RNA is a key contributor in countless biological processes. Though coding RNA has a well-known role in the central dogma of biology, most RNA is non-coding (*nc*RNA) and plays a multitude of roles including regulation and catalysis [1–6]. As a result, targeting *nc*RNA through molecular recognition would afford important tools for molecular biology and biotechnology [7]. One approach focuses on recognition of double-helical regions of RNA (dhRNA) using oligomers called triplex-forming oligonucleotides (TFOs) [8]. TFOs are utilized in both RNA and DNA recognition [9–12] and a particular type of TFO, peptide nucleic acid (PNA), has emerged as a promising probe for recognizing *nc*RNA [13–16].

The Nielsen lab first designed PNA with the *N*-(2-aminoethyl)glycine backbone for the recognition of DNA via triplex formation [17–18]. In this approach, PNA has several advantages compared to other TFOs. PNA was originally designed to be a charge neutral oligonucleotide that reduces electrostatic repulsions with the anionic phosphate backbone of DNA. Additionally, PNA is stable to nucleases, and it is easily prepared using well established peptide synthesis protocols, allowing for accessible incorporation of synthetic nucleobases [17–18].

**Figure 1:**
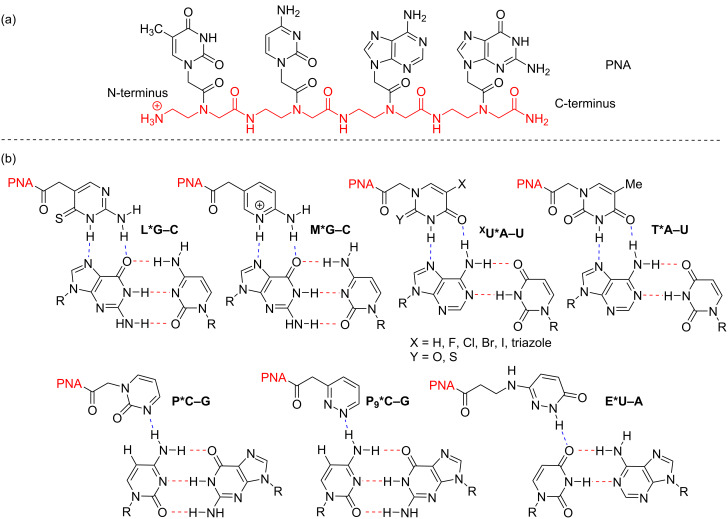
(a) Structure of a PNA oligomer with the *N*-(2-aminoethyl)glycine backbone (in red); (b) Representative monocyclic synthetic nucleobase triples through Hoogsteen hydrogen bonding (blue dashed lines). Watson–Crick hydrogen bonds are in red (dashed lines).

With these advantages in mind, Rozners’ lab first demonstrated in 2010 that PNA not only binds favorably and quickly to RNA, but that it binds more than 10 times stronger to RNA than to DNA [19]. Since the seminal report, much work has focused on exploring synthetic nucleobases in efforts to develop tools for sequence selective recognition of any RNA [13]. Figure 1 shows several of the most common monocyclic nucleobases used for each of the four possible base pairs (G–C, A–U, C–G, and U–A). Notably, each synthetic nucleobase takes advantage of favorable hydrogen bonding interactions to the proximal nucleobase in the Watson–Crick base pair. Strong binding has been reported for purine recognition given the propensity for two Hoogsteen hydrogen bonds. This includes G-recognition using 4-thiopseudoisocytidine (L) introduced by Chen [20–22] and 2-aminopyridine (M) utilized by Rozners [23–25]. For A-recognition, 5-halouracils (^X^U) [26] and 2-thiouracil [27] were both reported by Chen to have improved binding to RNA over the commonly used T monomer. We reported a similar result for 5-triazolyluracil [28]. However, despite the development of pyrimidine-recognizing bases (P, P_9_, E, etc.), the sequence selective recognition of any double helical region of RNA remains an elusive problem owing to the difficulties in pyrimidine recognition via a single hydrogen bond [29–30].

**Figure 2:**
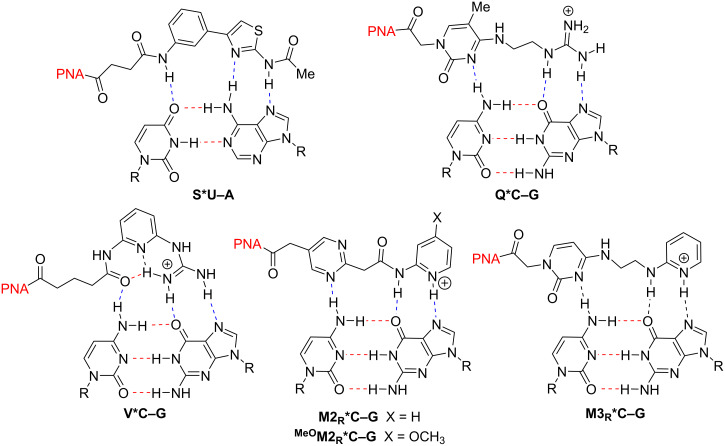
Representative extended nucleobase triples through Hoogsteen hydrogen bonding (blue dashed lines). Watson–Crick hydrogen bonds are in red (dashed lines).

In efforts toward improving pyrimidine recognition, several groups have explored PNA nucleobase monomers using extended nucleobases to utilize maximum hydrogen bonding interactions across the Hoogsteen face of the Watson–Crick base pair [13,15]. Notable examples of the PNA extended nucleobase for pyrimidine recognition of RNA include *N-*(4-(3-acetamidophenyl)thiazol-2-yl)acetamide (S) [31] and *N*^4^-(2-guanidoethyl)-5-methylcytosine (Q) [32] used by the Chen group, and cationic nucleobases such as 2-guanidylpyridine (V) [33] and substituted aminopyridine bases (M2_R_, ^MeO^M2_R_ and M3_R_) [28] reported by us (Figure 2). Despite significant effort, most known extended nucleobases show only modest selectivity and/or can only be used for a single pyrimidine interruption without significant loss in binding affinity.

**Figure 3:**
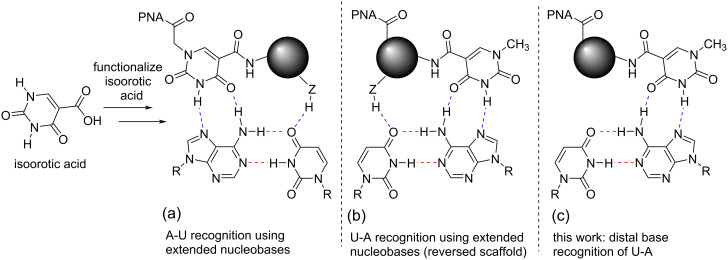
Evolution of the strategy for U–A recognition.

Given the challenges outlined above, our group has approached the pyrimidine recognition problem with a threefold strategy (Figure 3). First, we aimed to focus primarily on U–A recognition due to the poor selectivity of E [30], the promiscuous behavior of S [31], and the synthetic advantages imparted by commercially available uracil derivatives that could be readily functionalized. We noted that isoorotic acid offered an opportune core from which to prepare extended nucleobase monomers through amide bond formation (Figure 3a) [34]. Secondly, the work began with the initial goal to find an extended nucleobase scaffold capable of strong binding to A and then reverse its point of connection to the PNA with the goal of U-recognition (Figure 3b).

**Figure 4:**
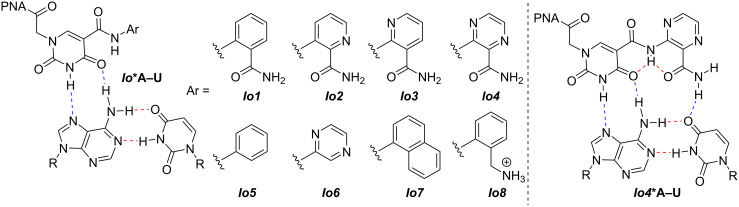
Isoorotamide-derived nucleobases for A–U recognition.

We first demonstrated that extended isoorotamide bases ***Io1****–****Io4*** (Figure 4) recognize A–U base pairs with good selectivity and affinity [35]. Using ***Io4***, this study highlighted the first example of a PNA with four consecutive extended nucleobases that showed enhanced triplex stability through cooperative effects compared to the control thymine base. In a follow up study, the pendant amide was removed from the ***Io*** core to determine the importance of the third hydrogen bond. To our surprise, these second-generation ***Io*** derivatives retained strong binding so long as the nucleobase lacked significant hydrophobicity [36]. This led to the hypothesis that quite possibly the third hydrogen-bond to the U base may not have been as important as originally believed.

**Figure 5:**
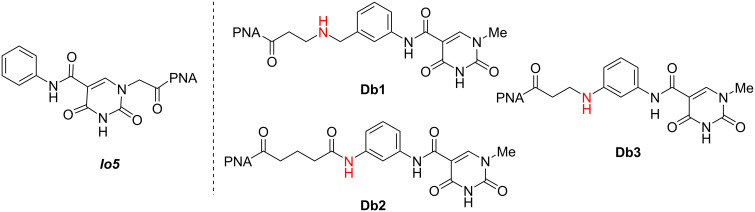
Proposed isoorotamide distal binding (**Db**) nucleobases designed from the ***Io5*** core. Hydrogen bonding donors to the linker are highlighted in red.

Given this result, we set out to design new nucleobases that reach across the entire Hoogsteen face of the RNA duplex allowing for recognition of U–A base pairs through a distal base recognition approach (Figure 3c). We proposed that by developing a linker of appropriate length that could reach across the uridine base, we could recognize the distal adenosine base and in turn essentially recognize U–A without formally hydrogen-bonding to U. This distal base recognition approach utilized design elements of our previous ***Io*** series [35–36], particularly ***Io5*** chosen for its synthetic simplicity and the ability to further functionalize the aromatic phenyl group. Three new nucleobases, **Db1**, **Db2**, and **Db3** were identified as targets (Figure 5). These monomers were informed by the linker design for V, which formed a stabilizing hydrogen bond between the linker NH and C=O of a linker connecting an adjacent M base to PNA backbone (vide infra) [33]. As a result, we sought to explore multiple N–H containing functional groups in the linker including an amine (**Db1**), an amide based on the V linker (**Db2**), and an aniline (**Db3**). In the case of **Db1**, physiological conditions would afford a protonated amine functionality that may allow for stabilization of the triplex through electrostatic interactions. Further, we intentionally varied the linker length from 4 to 6 atoms.

We describe herein the synthesis of the individual monomers, their incorporation into PNA oligomers, and the evaluation of those oligomers through UV-melting experiments.

## Results and Discussion

### Synthesis of Db PNA monomers

#### **Db1** Synthesis

**Scheme 1:**
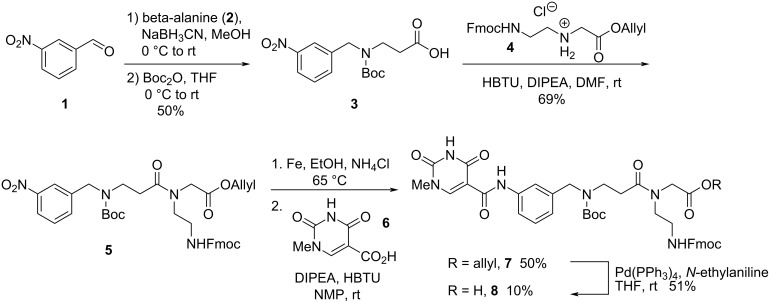
Synthesis of the **Db1** monomer (**8**).

The synthesis of **Db1** began from 3-nitrobenzaldehyde (**1**), which was treated with beta-alanine (**2**), in a reductive amination followed by subsequent Boc protection to afford **3** in 50% yield over two steps (Scheme 1). The stoichiometry of both the reducing agent and the beta-alanine proved important as incomplete conversion resulted in Boc protected beta-alanine that was challenging to separate from the desired product (**3**). Carboxylic acid **3** then underwent a peptide coupling reaction with allyl-protected PNA backbone **4** to afford nitrobenzene **5** in 69% yield. Nitrobenzene **5** was then reduced to the corresponding aniline with iron metal. Notably, the use of HCl as a proton source in the reduction led to significant removal of the Boc group necessitating the use of ammonium chloride as the proton source. The crude aniline was coupled to *N*-methylisoorotic acid **6** [37] to afford **7** in 50% yield. Surprisingly 10% of the final desired monomer **8** was also isolated in the coupling step, presumably due to hydrolysis of the allyl ester in the iron reduction step. The remaining allyl ester **7** was deallylated via Pd(PPh_3_)_4_ to afford the monomer **8** in 51% yield.

#### **Db2** Synthesis

**Scheme 2:**
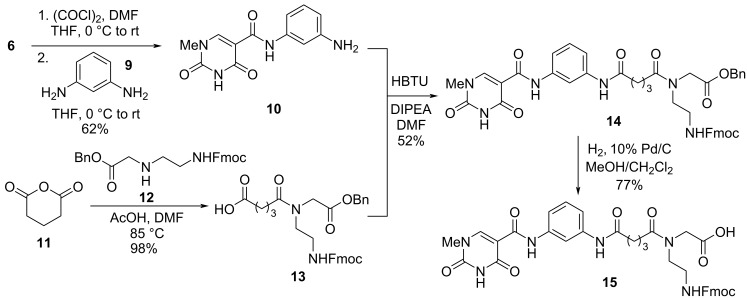
Synthesis of **Db2** monomer **15**.

**Db2** was prepared through a convergent synthesis of aniline **10** and carboxylic acid **13**. To access **10**, the isoorotic acid derivative **6** was treated with oxalyl chloride to form the corresponding acyl chloride in situ which was then slowly added to a solution of *m*-phenylenediamine (**9**) to afford the target aniline in 62% yield (Scheme 2). Slow addition of the acyl chloride to excess **9** was important to avoid forming bis-acylation of the phenylenediamine. The carboxylic acid **13** was formed using a microwave-promoted ring opening of glutaric anhydride (**11**) and benzyl-protected PNA backbone **12** [38]. A series of optimizations was performed in a microwave reactor with LCMS analysis, selecting for high conversion and minimal reaction time (Table 1). While high heat was originally feared to cause side reactions due to possible lability of the Fmoc and internal cyclization to form a piperazinone [39], it was discovered that the monomer was stable up to 100 °C. Table 1, entries 2–5 show that extended heat and time do not necessarily increase conversion for this reaction with the conversion capping at just over 90%. However, the conversion was improved when the reaction was performed with a greater excess of glutaric anhydride (Table 1, entries 6–8). Unreacted anhydride in this reaction could be easily removed during workup. In entry 8, >97% conversion was obtained in only 5 minutes with a 98% isolated yield. While a microwave was exclusively used to study this reaction at high temperatures, it is reasonable to expect that the reaction would perform equally well using conventional heating methods.

**Table 1:** Microwave promoted optimization of glutaric anhydride opening with **12**^a^.

Entry	Equivalents of **11**	Temp (°C)	Time (min)	% Conversion

1	1.32	75	15	90
2	1.32	85	7.5	93
3	1.32	95	3.75	93
4	1.32	95	7.5	90
5	1.32	100	7.5	92
6	1.64	75	15	97
7	1.64	85	7.5	97
**8**	**1.64**	**100**	**5**	**>97**

^a^Reactions were run at a 1.2 mmol scale in 7 mL DMF and 100 μL AcOH. % Conversion determined from relative peak areas on LCMS chromatograms comparing unreacted **12** to **13** taken from the crude solution.

Aniline **10** and carboxylic acid **13** were combined under standard (HBTU) amide coupling conditions to afford benzyl ester **14** in 52% yield. Compound **14** was subsequently debenzylated via hydrogenolysis to afford the target **Db2** monomer **15** in 77% yield.

#### **Db3** Synthesis

**Scheme 3:**
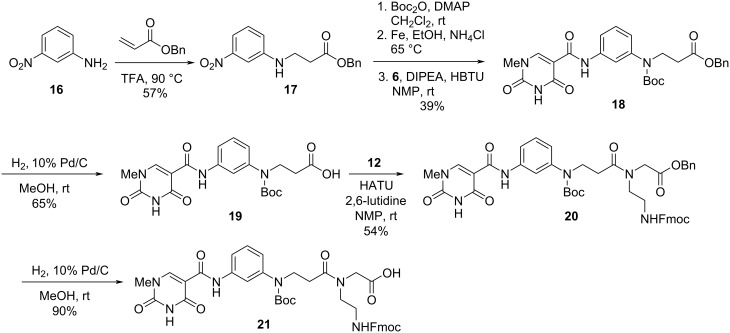
Synthesis of **Db3** monomer **21**.

The synthesis of **Db3** was originally envisioned to be completed with an unprotected aniline nitrogen, however, this proved problematic in the PNA synthesis. The ultimate route was developed to have an *N*-Boc protected monomer (Scheme 3). The synthesis started with a conjugate addition of *m*-nitroaniline into benzyl acrylate to afford compound **17** in 57% yield (Scheme 3). Aniline **17** then was subjected to a three-step Boc protection, nitro reduction, and coupling with isoorotic acid derivative **6** that afforded **18** in 39% yield over 3 steps. The benzyl ester was then cleaved under hydrogenolysis conditions to afford carboxylic acid **19** in 65% yield, followed by coupling with the PNA benzyl backbone **12** [38] to provide ester **20** in 54% yield. The final **Db3** monomer **21** was obtained in 90% yield through benzyl cleavage using the standard hydrogenolysis conditions, similar to previous monomers.

#### PNA Synthesis and biophysical assays

**Figure 6:**
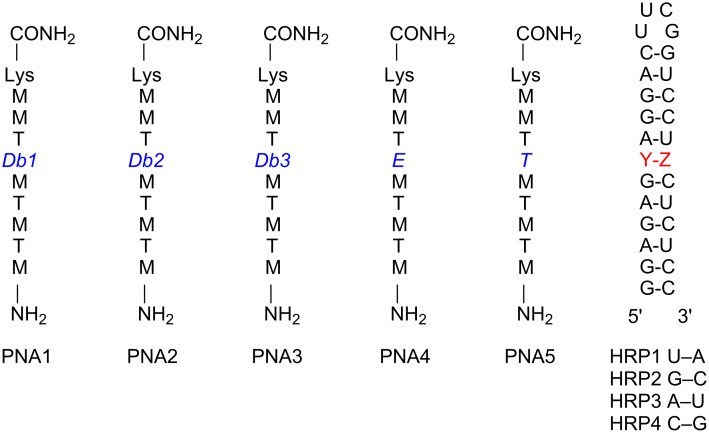
Sequences for RNA hairpins and PNA ligands used for binding studies.

With **Db1–Db3** monomers prepared, new nucleobases were incorporated into PNA oligonucleotides (PNA1–PNA3) via solid phase peptide synthesis using Fmoc chemistry on an Expedite 8909 DNA/RNA/PNA synthesizer and following established protocols [40]. Following purification by reversed-phase HPLC, these PNAs were studied against model RNA hairpins containing a purine-rich 5’-end and a variable base pair, Y–Z (Figure 6). The expected matched HRP1 (Y = U; Z = A) was designed to test affinity for the newly prepared **Db** nucleobases and mismatched HRP2–HRP4 were utilized to test selectivity for each of the remaining three mismatched base pair combinations.

Binding studies were then performed by annealing each of the PNA strands (PNA1–PNA3) with the four RNA hairpins (HRP1–HRP4). UV thermal melting studies reveal an inflection point at the temperature where the PNA dissociates from the RNA hairpin (Figures S4–S6, Supporting Information File 1). Table 2 shows that these new monomers are relatively weak binders for the matched HRP1 where melting experiments demonstrate *T*_m_ values ≈40 °C. In contrast, typical strong binders afford *T*_m_ values of >50 °C as shown by controls PNA4 and PNA5 [30]. Further, isothermal titration calorimetry experiments for PNA containing **Db1**–**Db3** verify this result with *K*_a_ values for PNA1–3 significantly lower than for controls PNA4 and PNA5. It was encouraging to determine that **Db1** and **Db3** have the expected higher affinity for the HRP1 (U–A base pair) compared to others, however, the difference is minimal. Still, the selectivity for **Db1** and **Db3** is comparable to or better than E, which currently is the gold standard for U-recognition. **Db2** surprisingly demonstrated slightly higher affinity for the C–G base pair than for the desired U–A base pair. For both UV melting and ITC, **Db2** showed especially poor affinity for the U–A base pair in HRP1.

**Table 2:** Binding data^a^ for PNA containing **Db** monomers using UV thermal melting^b^ and isothermal titration calorimetry.^c^

PNA X	HRP1 (X*U–A)	HRP 2 (X*G–C)	HRP 3 (X*A–U)	HRP 4 (X*C–G)	ITC

PNA1(**Db1**)	**41.8 ± 0.4**	35.9 ± 0.5	36.1 ± 0.3	38.0 ± 0.5	4.6 ± 0.1^d^
PNA2 (**Db2**)	36.2 ± 0.2	27.5 ± 0.6	33.3 ± 0.5	**41.4 ± 0.2**	1.0 ± 0.0^d^
PNA3 (**Db3**)	**41.3 ± 0.3**	27.5 ± 0.3	34.4 ± 0.2	38.3 ± 0.4	3.2 ± 0.2^d^
PNA4 (**E**)^e^	**53.8 ± 0.6**	33.7 ± 0.5	49.6 ± 0.3	49.0 ± 0.4	11 ± 1^d^
PNA5 (**T**)^e^	34.6 ± 0.2	46.4 ± 0.5	**69.6 ± 0.8**	35.4 ± 0.4	12 ± 1^f^

^a^All binding data conducted in 50 mM potassium phosphate buffer (pH 7.4) containing 2 mM MgCl_2_, 90 mM KCl, and 10 mM NaCl. ^b^UV melting temperatures (*T_m_*, °C) are averages of six experiments ± the standard deviation measured at 300 nm and 18 µM of each RNA hairpin and PNA. ^c^Association constants *K*_a_ × 10^6^ M^−1^, average of three experiments ± standard deviation, for binding of PNA with the matched RNA hairpin. ^d^ITC using HRP1. ^e^Reference [30]. ^f^ITC using HRP3.

**Figure 7:**
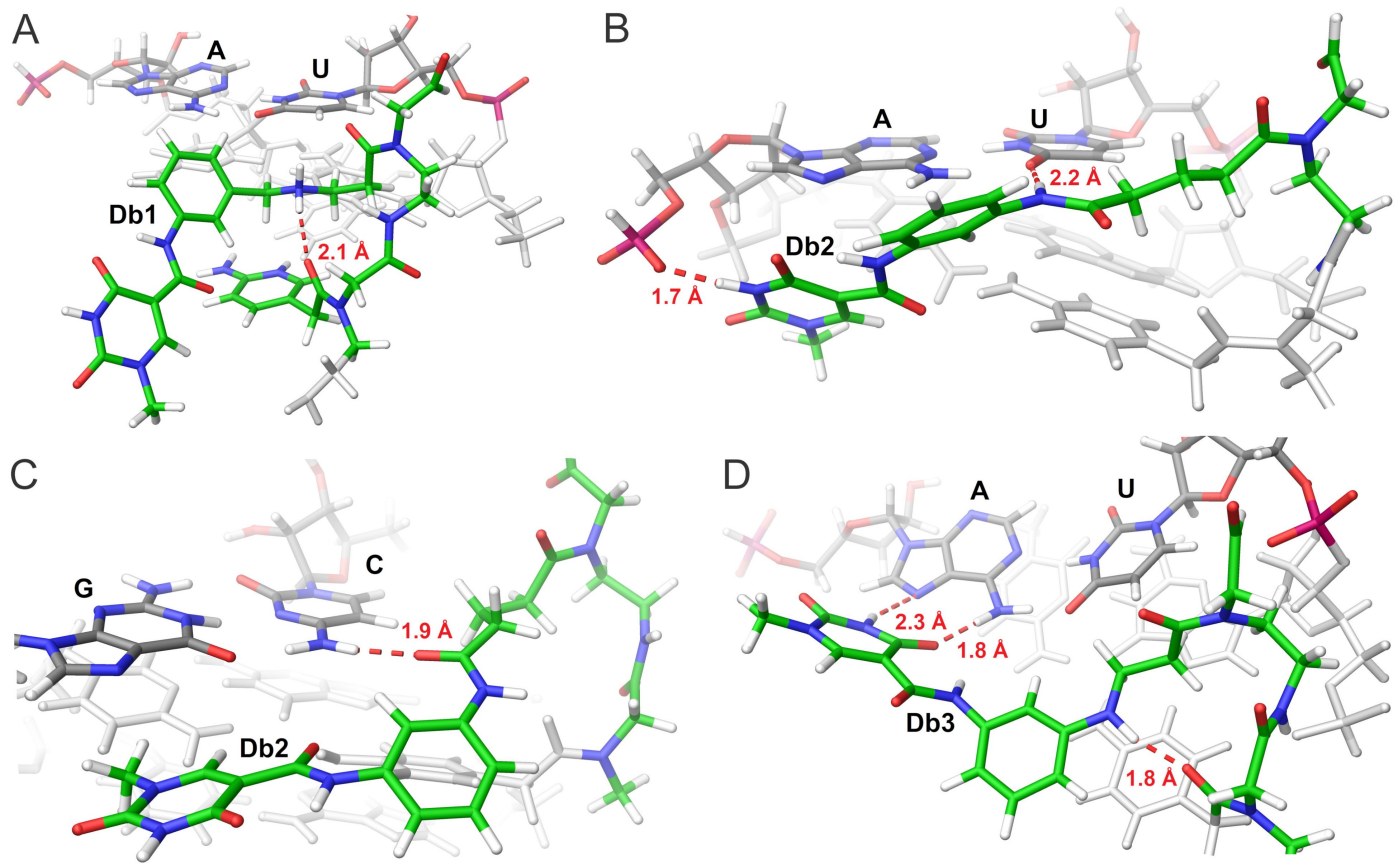
Major-groove view of hydrogen-bonding interactions in the (A) Db1*U–A triplet, (B) Db2*U–A triplet, (C) Db2*C–G triplet, and (D) Db3*U–A triplet. Carbon, hydrogen, oxygen, and nitrogen are labelled in green, white, red, and blue, respectively.

To help explain experimental binding results, we turned to computational molecular dynamics modeling using HRP1 and PNA1–3 as a model based on the PNA–dhRNA triplex provided by previous NMR studies [41] (for details see Supporting Information File 1). The **Db** nucleobases were individually incorporated into the PNA model oligonucleotide and subjected to 50 ns unrestricted Desmond molecular dynamics. Pictures from the molecular dynamics simulations shown in Figure 7 represent the conformation of the PNA bases with the highest probability.

The snapshots in Figure 7 reveal problems with the **Db** bases in the binding pocket. As discussed earlier, each **Db** base has a linker containing an NH moiety aimed at forming a stabilizing hydrogen bond with the carbonyl from the linker of the adjacent PNA M-base. Such a phenomenon was observed previously with V and this design element was intentionally incorporated with an expectation toward aiding in triplex stability [33]. For **Db1**, calculations reveal that the intended H-bond between the cationic –NH_2_^+^– moiety did in fact form (ca. 2.1 Å, Figure 7A). However, the distal portion of the nucleobase (the isoorotamide) simply dissolved, or rotated away from the binding pocket, not forming the intended interaction with distal A base in silico (Figure 7A). In the case of **Db2**, the amide NH in the linker was apparently too far from the PNA backbone and did not form the intended stabilizing H-bond but rather formed a hydrogen bond with the U in the U–A base pair (ca. 2.2 Å, Figure 7B). This interaction resulted in pushing the entire residue towards the RNA backbone where the ***Io*** base could further bind to a phosphate which likely explains why **Db2** shows the lowest triplex stability of the three bases. Given that the **Db2** linker is the same as that for V, it is not surprising to observe that binding for C–G was slightly higher than that of U–A and we propose that the binding of the **Db2** linker with C may mimic the binding of V [33] and other synthetic nucleobases with linkers derived from glutaric anhydride [30]. In fact, molecular dynamics simulations did confirm that the amide carbonyl of the linker forms a stable H-bond to the amino group of C (Figure 7C). Since the distal guanosine in the C–G base pair is a mismatch with the isoorotic acid binding moiety, the isoorotamide, heterocycle of **Db2** is rotated, placing its polar face towards solute and non-polar methyl group towards RNA. This results in the whole **Db2** base being tilted, which breaks the intended stabilizing H-bond between the **Db2** linker and the adjacent M. **Db3** resulted in the best interaction in silico despite the low binding affinities. In this case, the ***Io*** base forms the key H-bonds to the distal A, and the anilino NH in the linker formed the intended hydrogen bond with the adjacent M-linker (Figure 7D). However, it is notable that for the isoorotamide to reach the distal A-base, a significant distortion of the phenyl group out of the plane of the isoorotamide is required. Such distortion likely causes a disruption in triplex stability.

Together, the UV melting data and computational modeling offer several lessons for future monomer design. First, despite the success of past nucleobases that incorporate a physiologically cationic nitrogen (i.e., M, V, and ***Io7****)*, an ammonium moiety in the linker seemingly does not assist in stabilizing binding to the RNA sequence, as is indicated by comparison of the modeling of **Db1** and the UV melting. Additionally, the extended length of the PNA monomer and linker brings in an entropic issue that may be challenging to overcome. To date, the best extended nucleobases have been V and ***Io4***, both of which contain an extensive hydrogen bonding network that likely pre-organizes and planarizes the π-system. All of the new monomers in this study contained much greater conformational flexibility. Yet, for the Hoogsteen hydrogen bonding to occur using distal recognition monomers, the entirety of the flexible linker must enter the major groove being forced into a conformation that no longer is freely rotating. Additionally, these longer chains bring in potential issues of steric conflict that add more complexity to triplex formation.

## Conclusion

Overall, three new PNA monomers were synthesized and incorporated into PNA oligonucleotides. **Db** monomers demonstrated a lack of strong affinity for the U–A base pair in dhRNA. However, selectivity of **Db1** and **Db3** for the intended U–A base pair was comparable to the selectivity of E and these scaffolds may present a lead for future nucleobase designs. The study further demonstrated a conflict between linker flexibility and triplex formation. However, the results point to the importance of entropy and related pre-organization of PNA bases for efficient binding and further underscore the complexity of these systems due to conformational and energetic preferences. Taken together with previous studies, we postulate that future nucleobases should be designed to overcome the positional challenges related to entropy and geometry.

## Supporting Information

File 1 General synthetic details and procedures, characterization data for synthetic intermediates, biophysical assays, and computational details.

## Data Availability

All data that supports the findings of this study is available in the published article and/or the supporting information of this article.
