# Supplementary material for: Isoorotamide-based peptide nucleic acid nucleobases with extended linkers aimed at distal base recognition of adenosine in double helical RNA
Source: Beilstein J Org Chem. 2025 Nov 12;21:2513–23. doi: 10.3762/bjoc.21.193 (PMC12621619; doi:10.3762/bjoc.21.193)
Supplement: File 1 — General synthetic details and procedures, characterization data for synthetic intermediates, biophysical assays, and computational details. [file Beilstein_J_Org_Chem-21-2513-s001.pdf]

BEILSTEIN JOURNAL OF ORGANIC CHEMISTRY

Supporting Information

for

Isoorotamide-based peptide nucleic acid nucleobases with extended linkers aimed at distal base recognition of adenosine in double helical RNA

Grant D. Walby, Brandon R. Tessier, Tristan L. Mabee, Jennah M. Hoke, Hallie M. Bleam, Angelina Giglio-Tos, Emily E. Harding, Vladislavs Baskevics, Martins Katkevics, Eriks Rozners and James A. MacKay

Beilstein J. Org. Chem. **2025,** *21,* 2513–2523. doi:10.3762/bjoc.21.193

General synthetic details and procedures, characterization data for synthetic intermediates, biophysical assays, and computational details

License and Terms: This is a supporting information file under the terms of the Creative Commons Attribution License (https://creativecommons.org/licenses/by/4.0). Please note that the reuse, redistribution and reproduction in particular requires that the author(s) and source are credited and that individual graphics may be subject to special legal provisions.
The license is subject to the *Beilstein Journal of Organic Chemistry* terms and conditions: (https://www.beilstein-journals.org/bjoc/terms)

Table of contents

General synthetic details S3

Procedures for synthesis of **Db1**, **Db2**, and **Db3** monomers S4

NMR Spectra for synthetic intermediates and monomers S25

Procedures for PNA synthesis, quantification, and purification..S42

Biophysical assays S47

Computational details..S53

References for SI S54

General synthetic details

All solvents and reagents were purchased commercially and used without further purification, with the exception of *m*-aminoaniline, diisopropylethylamine (DIPEA), tetrahydrofuran (THF), and dimethylformamide (DMF). Commercial *m*-aminoaniline (**9**) was sublimed under vacuum to afford a white solid. DIPEA was distilled over CaH_2 and stored under N_2 in a Schlenk flask, THF was distilled over sodium and benzophenone, and DMF was stored under N_2 in a Schlenk flask. All water was deionized unless otherwise stated. All reactions were performed with oven dried glassware under an inert atmosphere (N_2 gas). Analytical thin layer chromatography (TLC) was carried out using silica gel 60 F_{254} glass plates for normal phase. TLCs were viewed through use of a UV lamp (254 and/or 365 nm). A *Teledyne ISCO* CombiFlash® EZ Prep flash chromatography system was used for all flash system chromatography. IR spectra were obtained using a Nicolet iS50 FT-IR with diamond ATR accessory. ^{1}H and ^{13}C NMR spectra were obtained with a Varian 400-MR spectrometer and samples prepared in 5 mm OD tubes with deuterated solvent. Chemical shifts (δ) were reported relative to the solvent peak ($CDCl_3$ or DMSO-d_6) as a reference. The following abbreviations (or combinations thereof) were used to describe multiplicities: s = singlet, d = doublet, t = triplet, q = quartet, m = multiplet, br = broad. Coupling constants (*J*) were reported in Hertz (Hz). High resolution mass spectra were obtained using a Q-Exactive Orbitrap high resolution mass spectrometer (Thermo Scientific) equipped with a heated electrospray (HESI) source. Data were analyzed using Xcalibur software(Thermo Scientific)

Procedures for synthesis of Db1, Db2 and Db3 monomers.

Experimental for Synthesis of ***Db1***

Compound 3:

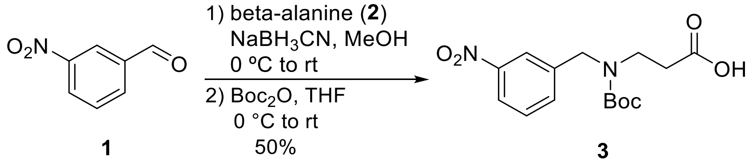

3-((tert-Butoxycarbonyl)(3-nitrobenzyl)amino)propanoic acid: 3-nitrobenzaldehyde (2.86 g, 18.2 mmol) was added to a solution of beta-alanine (1.08 g, 12.1 mmol) and AcOH (800 mg, 13.3 mmol, 0.76 mL) in MeOH (48 mL) at room temperature. The solution was stirred for 5 h whereupon the reaction was cooled to 0 °C and $NaCNBH_3$ (1.14 g, 18.2 mmol) was added. The reaction was warmed to room temperature and stirred overnight. The solution was concentrated under reduced pressure and the crude residue was dissolved in a 1:1 H_2O:1,4-dioxane (48 mL). Boc_2O (5.29 g, 24.2 mmol) and Et_3N (3.68 g, 36.4 mmol, 5.1 mL) were added to the reaction mixture and the solution was stirred at room temperature overnight. The solution was concentrated under reduced pressure and the residue was cooled to 0 °C whereupon 10% HCl was added until a pH of 2 was obtained. The aqueous layer was extracted with EtOAc (3 × 50 mL). The organic layers were combined and washed with H_2O (50 mL) and brine (50 mL). The organic layer was dried ($MgSO_4$) and was concentrated under reduced pressure. The crude product was purified via column chromatography (SiO_2) eluting with hexane:EtOAc (80:20 → 60:40 → 20:80 → 0:100) to afford 1.97 g (50% over two steps) of **3** as a white solid. Analytical TLC, 25:75 hexanes/EtOAc eluent, R_f = 0.16. HRMS (ESI) m/z: $[M – H]^-$ calcd for $C_{15}H_{19}N_2O_6$ 323.1249; found 323.12469. IR (neat, cm^{-1}) 2973, 1691, 1526, 1157. ^1H NMR (400 MHz, DMSO-d_6, ppm) δ 12.25 (1H, s), 8.40-7.87 (2H, m), 7.85-7.46 (2H, m) 4.53 (2H, s), 3.40 (2H, s),

2.46 (2H, t), 1.39 (9H, d, J = 33.0 Hz). ^{13}C NMR (101 MHz, DMSO-d$_6$, ppm, mixture of rotamers) δ 173.0, 155.2, 154.7, 148.1, 141.4, 134.0, 130.2, 122.2, 121.9, 79.7, 50.1, 49.3, 43.4, 33.5, 33.1, 28.1.

Compound 5:

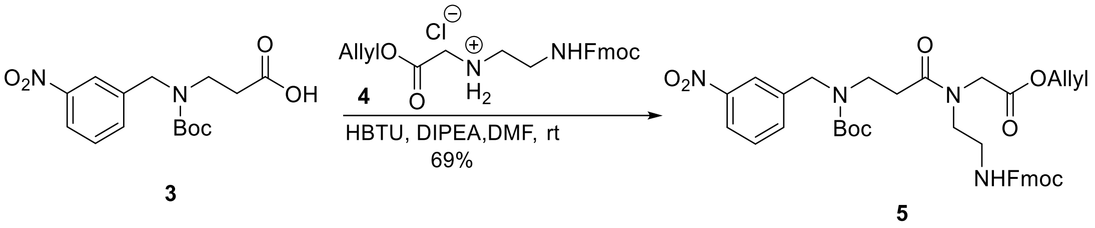

Allyl *N*-(2-((((9*H*-fluoren-9-yl)methoxy)carbonyl)amino)ethyl)-*N*-(3-((*tert*-butoxycarbonyl)(3-nitrobenzyl)amino)propanoyl)glycinate: In a round bottom flask, compound **3** (575 mg, 1.77 mmol) and HBTU (672 mg, 1.77 mmol) were combined and purged with N_2 for 30 minutes, then the solids were dissolved in dry DMF (16.7 mL). In a separate 50 mL flask, allyl protected backbone **4** [1] (808 mg, 2.12 mmol) was purged under N_2 for 30 minutes before the solution of **3** and HBTU in DMF was cannulated into the flask. The reaction flask was cooled to 0 °C before dropwise addition of DIPEA (617 µL, 3.54 mmol) over 60 minutes. The reaction mixture was warmed to room temperature while stirring under N_2 for 24 h. The mixture was concentrated to afford a thick oil, which was dissolved in CH_2Cl_2 (75 mL), and washed with saturated $NaHCO_3$ (75 mL). The aqueous layer was back-extracted with CH_2Cl_2 (65 mL × 2) and the combined organic layers were washed with brine (75 mL), dried with anhydrous Na_2SO_4, and concentrated. The crude brown oil was dissolved in CH_2Cl_2, dry loaded on celite, and purified by a manual column using a silica gel column (3.5 cm × 15 cm) with a 50–100% gradient of EtOAc in hexanes. The column purification afforded 836 mg (69%) **5** as a white glassy foam. Analytical TLC, 3:1 EtOAc/hexanes eluent, R_f = 0.42. HRMS (ESI) m/z: $[M+H]^+$

calcd for $C_{37}H_{43}N_4O_9$ 687.3025; found 687.30133. IR (neat, cm^{-1}) 2944, 1691, 1646, 1528, 1348, 1158. ^{1}H NMR (400 MHz, DMSO-d_6, ppm) δ 8.15 – 8.01 (m, 2 H), 7.88 (ddt, *J* = 7.57, 3.85, 0.98 Hz, 2 H), 7.73 – 7.53 (m, 4 H), 7.45 – 7.17 (m, 5 H), 6.02 – 5.77 (m, 1 H), 5.40 – 5.15 (m, 2 H), 4.66 – 4.42 (m, 4 H), 4.37 – 4.15 (m, 3 H), 4.03 (s, 1 H), 3.43 – 3.35 (m, 2 H), 3.32 (s, 2 H), 3.21 – 3.02 (m, 2 H), 2.62 (t, *J* = 7.17 Hz, 1 H), 1.37 (d, *J* = 42.59 Hz, 9 H). ^{13}C NMR (101 MHz, DMSO-d_6, ppm) δ 171.4 (HMBC), 169.5, 169.0, 156.2, 147.8, 143.8, 140.7, 140.7, 133.7, 132.3, 130.0, 127.6, 127.6, 127.0, 125.1, 125.0, 122.0, 121.6, 121.5, 120.1, 117.7, 79.4, 65.4, 65.3, 64.8, 47.6, 46.7, 43.4, 38.2, 27.9.

Compound 6:

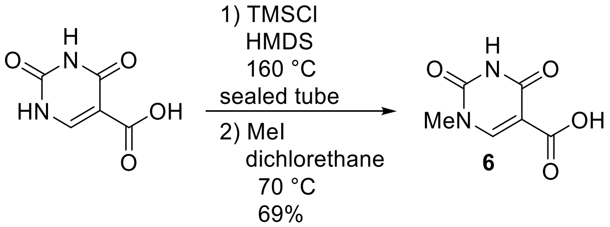

1-Methyl-2,4-dioxo-1,2,3,4-tetrahydropyrimidine-5-carboxylic acid: To a 150 mL pressure vessel was added isoorotic acid (13.75 g, 88.1 mmol), TMSCl (11.2 mL, 88.1 mmol), and HMDS (110 mL), yielding a cloudy solution which was stirred at 160 ˚C for 24 h. The solution was brought to room temperature, transferred to a round bottom flask, and the solvent was evaporated in vacuo, affording a light-yellow oil. Dichloroethane (110 mL) and methyl iodide (11 mL, 180 mmol) were added to the round bottom flask and the suspension was heated at 70 ˚C for 20 hours. Upon completion, the solution was cooled to room temperature and then added to a cold (0 °C) solution of 1:1 H_2O/acetic acid. The mixture was stirred at 0 °C for 30 minutes. The resulting solid was filtered, washed with cold water (150 mL) and cold ethyl acetate (110 mL). The resulting light yellow amorphous powder was dried in vacuo to give 10.4 g of **6** (69%)

with spectral properties that matched the literature [2,3]. ^{1}H NMR (400 MHz, DMSO-d$_6$, ppm) δ: 11.20 (s, 1 H), 7.61 (d, J = 7.8 Hz, 1 H), 5.51 (d, J = 7.8 Hz, 1H), 3.22 (s, 3 H).

Compound 7:

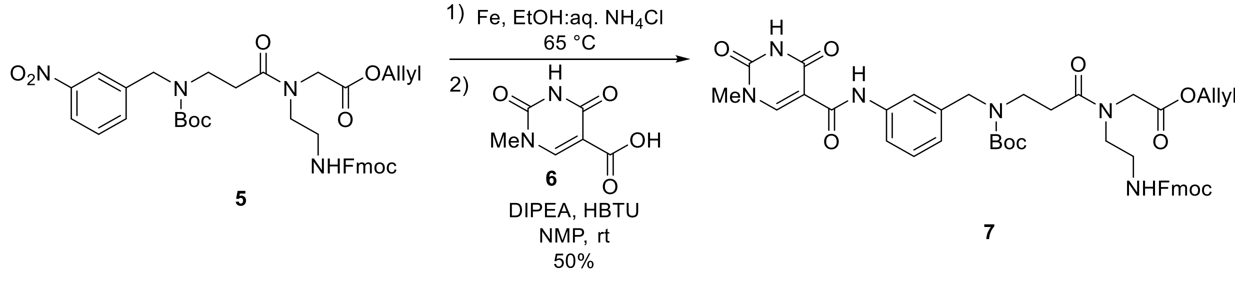

Allyl *N*-(2-((((9*H*-fluoren-9-yl)methoxy)carbonyl)amino)ethyl)-*N*-(3-((*tert*-butoxycarbonyl)(3-(1-methyl-2,4-dioxo-1,2,3,4-tetrahydropyrimidine-5-carboxamido)benzyl)amino)propanoyl)glycinate: Iron powder (926 mg, 16.6 mmol) was added to a solution of **5** (1.14 g, 1.66 mmol) in 1:1 EtOH:aq. NH_4Cl (34 mL) at room temperature. The solution was then heated to 75 °C and stirred for 2.5 h. The solution was cooled to room temperature and was filtered through celite. The celite was washed with MeOH (150 mL) and the filtrate was concentrated under reduced pressure. The resulting aqueous solution was extracted with EtOAc (3 × 100 mL). The organic layers were combined, dried ($MgSO_4$) and concentrated under reduced pressure to crude product as a brown oil that was directly taken to the next reaction. Analytical TLC, 0:100 hexanes/EtOAc eluent, R_f = 0.71.

DIPEA (310 mg, 2.40 mmol, 0.42 mL) was added to solution of **6** (215 mg, 1.26 mmol) and HATU (480 mg, 1.26 mmol) in NMP (5.5 mL) at room temperature. The solution was stirred for 30 minutes whereupon it was transferred via cannula to a solution of crude iron reduction product (790 mg, 1.20 mmol) in NMP (5.5 mL). The solution was then stirred at room temperature overnight. The next day the solution was concentrated under reduced pressure and

the remaining oil was diluted in a 3:1 mixture of $CHCl_3$:iPrOH (75 mL). The organic layer was washed with H_2O (4 × 50 mL), sat. $NaHCO_3$ (25 mL), and brine (25 mL). The organic layer was then collected, dried ($MgSO_4$) and concentrated under reduced pressure. The crude product was purified via automated flash chromatography using a silica gel column (24 g, 35 mL/min) eluting with a MeOH:CH_2Cl_2 gradient (0:100 → 15:85) to afford 631 mg of **7** (50% over two steps) as a waxy yellow oil and 157 mg of **8** (10% over two steps) as a thick yellow oil.

Compound **7**: HRMS (ESI) m/z: $[M + H]^+$ calcd for $C_{43}H_{49}N_6O_{10}$ 809.3505; found 809.34913. IR (neat, cm^{-1}) 2970, 1722, 1686, 1448, 1409, 1161. ^1H NMR (400 MHz, DMSO-d_6, ppm, as a mixture of rotamers) δ 12.04 (s, 1 H), 10.95 (s, 1 H), 8.61 (s, 1 H), 7.87 (dd, *J* = 7.65, 3.84 Hz, 2 H), 7.65 (d, *J* = 7.55 Hz, 2 H), 7.58 – 7.17 (m, 8 H), 6.91 (d, *J* = 7.59 Hz, 1 H), 5.89 (dt, *J* = 11.35, 5.68 Hz, 1 H), 5.25 (dd, *J* = 41.00, 13.97 Hz, 2 H), 4.59 (dd, *J* = 21.65, 5.47 Hz, 2 H), 4.43 – 4.11 (m, 7 H), 4.03 (s, 1 H), 3.40 (s, 3 H), 3.19 – 3.02 (m, 2 H), 2.61 (s, 1 H), 2.43 (br s, 1 H), 2.17 (t, *J* = 8.09 Hz, 2 H), 1.90 (p, *J* = 7.51 Hz, 2 H), 1.39 (d, *J* = 20.29, 9 H). ^{13}C NMR (101 MHz, DMSO-d_6, ppm, as a mixture of rotamers) δ 174.5 (HSQC), 172.1 (HSQC), 169.6 (HSQC), 164.7 (HSQC), 160.2, 152.7, 150.3, 143.8, 140.7, 132.3, 127.6, 127.0, 125.0, 122.5, 120.1, 117.7, 103.9, 79.2, 64.8, 62.0, 48.5, 46.7, 36.3, 30.1, 29.0, 28.0, 17.2.

Compound 8 (Db1):

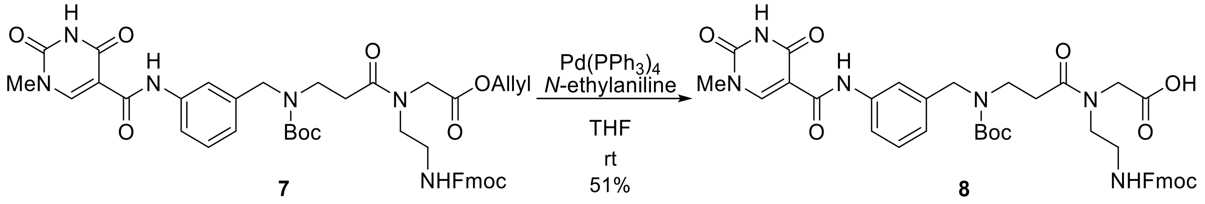

***N*-(2-((((9*H*-Fluoren-9-yl)methoxy)carbonyl)amino)ethyl)-*N*-(3-((tert-butoxycarbonyl)(3-(1-methyl-2,4-dioxo-1,2,3,4-tetrahydropyrimidine-5-carboxamido)benzyl)amino)propanoyl)glycine**: Anhydrous THF (2 mL) was added to a mixture of Pd(PPh_3) (10.7 mg, 0.0093 mmol) and **7** (150 mg, 0.185 mmol) at room temperature whereupon the solution was stirred for 15 minutes. *N*-Ethylaniline (44.9 mg, 50 µL) was added and the reaction was stirred for 3.5 h upon which the solution was concentrated under reduced pressure. The residue was redissolved in CH_2Cl_2 (25 mL) and was washed with 10% w/v aq. $KHSO_4$ (10 mL). The organic layer was collected, dried ($MgSO_4$), and concentrated under reduced pressure. The crude product was purified via column chromatography (SiO_2) eluting with EtOAc:MeOH (100:0 → 95:5 → 90:10) to afford 72.5 (51%) **8** as a glassy yellow solid. Analytical TLC, 100% EtOAc eluant, R_f = 0.13. HRMS (ESI) m/z: $[M + H]^+$ calcd for $C_{40}H_{45}N_6O_{10}$ 769.3192; found 769.31891. IR (neat, cm^{-1}) 3268, 2972, 1718, 1687, 1643, 1617, 1448, 1248, 1161. ^1H NMR (400 MHz, DMSO-d_6, ppm) δ 12.04 (s, 1 H), 10.95 (s, 1 H), 8.61 (s, 1 H), 7.87 (dd, *J* = 7.54, 4.56 Hz, 2 H), 7.65 (d, *J* = 7.50 Hz, 2 H), 7.56 – 7.20 (m, 8 H), 6.92 (d, *J* = 7.63 Hz, 1 H), 4.50 – 4.14 (m, 6 H), 4.09 – 3.99 (m, 1 H), 3.91 (s, 1 H), 3.40 (s, 3 H), 3.20 – 3.04 (m, 2 H), 2.69 (s, 1 H), 2.61 (d, *J* = 7.28 Hz, 1 H), 2.50 – 2.40 (m, 1 H), 2.17 (t, *J* = 8.03 Hz, 1 H), 1.90 (p, *J* = 7.54 Hz, 1 H), 1.39 (d, *J* = 22.89 Hz, 9 H). ^{13}C NMR (101 MHz, DMSO-d_6, ppm) δ 171.5 (HMBC), 164.2, 160.2, 152.7, 150.3, 143.8, 140.7, 140.7, 138.4, 127.6, 127.6, 127.0, 125.0, 122.5, 120.1, 118.2, 103.9, 79.2, 65.4, 62.0, 46.7, 36.3, 28.0, 28.0, 25.5.

Compound SI - 1:

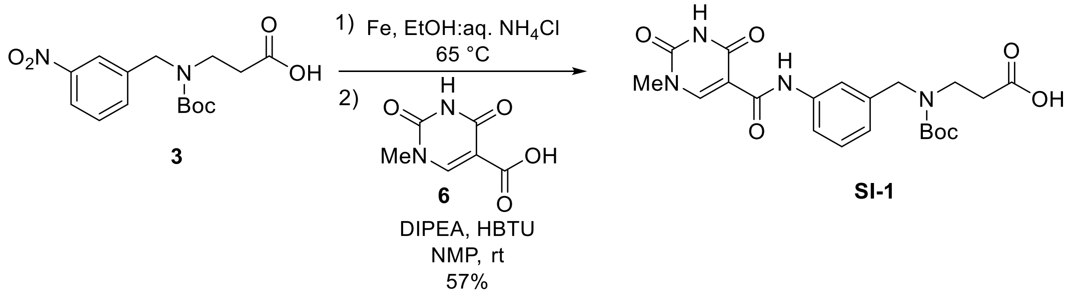

3-((*tert*-Butoxycarbonyl)(3-(1-methyl-2,4-dioxo-1,2,3,4-tetrahydropyrimidine-5-carboxamido)benzyl)amino)propanoic acid. Fe (496 mg, 8.88 mmol) was added to a solution of **3** (576 mg, 1.78 mmol) in a 2:1 mixture of EtOH:aq. NH_4Cl (8.9 mL) at room temperature. The solution was heated to 65 °C and stirred overnight. The solution was allowed to cool to room temperature, filtered through a pad of celite, and the celite was then washed with EtOH (50 mL). The organic solution was then concentrated under reduced pressure and the aqueous layer was extracted with EtOAc (3 ≈ 50 mL). The organic layers were combined, dried ($MgSO_4$), and concentrated under reduced pressure. The crude product was then taken directly to the next reaction.

HBTU (459 mg, 1.21 mmol) was added to a solution of **6** (206 mg, 1.21 mmol) in anhydrous NMP (10 mL) at room temperature and the solution was stirred for 3 h followed by addition of a solution of crude iron reduction product (375 mg, 1.27 mmol) in NMP (7 mL) at room temperature. The reaction was stirred for 30 minutes whereupon DIPEA (329 mg, 2.55 mmol, 0.45 mL) was added at room temperature and the solution was stirred overnight. The solution was then concentrated under reduced pressure and the resultant residue was redissolved in a 3:1 mixture of $CHCl_3$:iPrOH (35 mL). The organic solution was sequentially washed with H_2O (3 × 25 mL), 10% w/v aq. $KHSO_4$ (15 mL) and brine (25 mL). The organic layer was collected, dried

($MgSO_4$), and concentrated under reduced pressure. The crude product was purified via automated flash chromatography using a HP silica gel column (12 g, 30 mL/min) eluting with a hexane:EtOAc gradient (100:0 → 0:100) to afford 306 mg (57% over 2 steps) of **SI-1** as a brownish-red solid. IR (neat, cm^{-1}) 2978, 1717, 1671, 1621, 1562, 1415, 1155. ^{1}H NMR (400 MHz, DMSO-d_6, ppm) δ 12.06 (s, 1 H), 10.97 (s, 1 H), 8.63 (s, 1 H), 7.64 – 7.38 (m, 2 H), 7.31 (t, *J* = 7.85 Hz, 1 H), 6.95 (d, *J* = 7.66 Hz, 1 H), 4.36 (s, 2 H), 3.41 (s, 3 H), 3.23 – 3.09 (br s, 2 H), 1.68 (p, *J* = 7.04 Hz, 2 H), 1.40 (d, *J* = 15.48 Hz, 9 H). ^{13}C NMR (101 MHz, DMSO-d_6, ppm) δ 174.2, 164.7, 160.6, 155.5 (HMBC) 153.2, 150.8, 140.6 (HMBC), 138.8, 129.5, 124.9, 118.6, 104.3, 79.3, 49.4, 46.1, 36.7, 28.5, 23.5.

Experimental for Synthesis of ***Db2***

Compound 10:

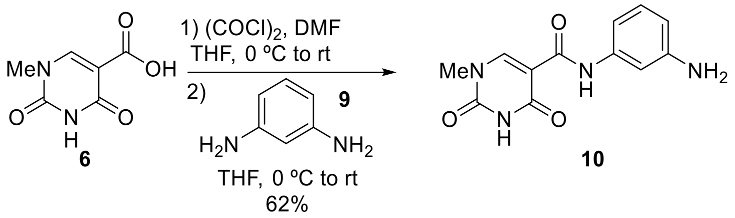

***N*-(3-Aminophenyl)-1-methyl-2,4-dioxo-1,2,3,4-tetrahydropyrimidine-5-carboxamide**: Compound **6** (500 mg, 2.94 mmol) was dissolved in THF (30 mL), cooled to 0 °C, and DMF (68 µL, 0.88 mmol) was added dropwise. Next, freshly distilled $(COCl)_2$ (0.63 mL, 7.35 mmol) was subsequently added slowly down the side of the flask, and the homogeneous mixture was allowed to passively warm to room temperature while stirring for 2 h. Volatiles were then removed via rotary evaporation under reduced pressure at 30 °C. The acyl chloride intermediate was redissolved in THF (60 mL) and cooled to 0 °C. Separately, *m*-aminoaniline (1.589 g, 14.70

mmol, freshly sublimed) was dissolved in THF (30 mL) and cooled to 0 °C. The isoorotyl chloride solution was then slowly added to the *m*-aminoaniline solution over 1 h at 0 °C. The cloudy yellow mixture was stirred at room temperature for 2 h, at which point TLC showed a disappearance of starting material. The mixture was then vacuum filtered and the solid was washed with 20% EtOH in H_2O (3 × 6 mL) and dried to afford 472 mg of **10** as a silvery white solid (62% over two steps). Analytical TLC, 9:1 CH_2Cl_2:MeOH eluent, R_f = 0.48. HRMS (ESI) m/z: $[M + H]^+$ calcd for $C_{12}H_{13}N_4O_3$ 261.0982; found 261.09808. IR (neat, cm^{-1}): 3443, 3359, 3044, 1740, 1709, 1678, 1606. ^1H NMR (400 MHz, DMSO-d_6, ppm) δ 12.0 (br s, 1 H), 10.7 (s, 1 H), 8.6 (s, 1 H), 7.0 (t, J = 7.8 Hz, 1 H), 6.9 (s, 1 H), 6.8 (d, J = 7.8 Hz, 1 H), 6.3 (d, J = 7.8 Hz, 1 H), 5.1 (br s, 2 H), 3.4 (s, 3 H). ^{13}C NMR (101 MHz, DMSO-d_6, ppm) 164.3, 159.8, 152.5, 150.4, 149.3, 138.8, 129.3, 109.8, 107.2, 104.8, 104.1, 36.3.

Compound 13:

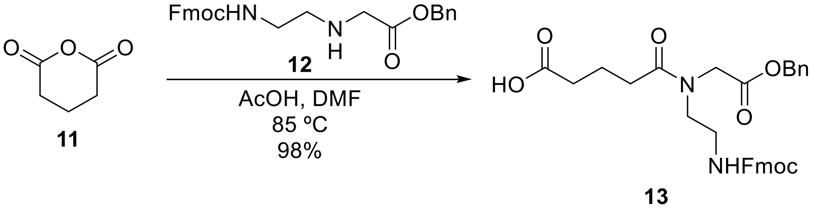

5-((2-((((9*H*-Fluoren-9-yl)methoxy)carbonyl)amino)ethyl)(2-(benzyloxy)-2-oxoethyl)amino)-5-oxopentanoic acid: Compound **12** [1] (500 mg, 1.20 mmol) was combined with glutaric anhydride (224 mg, 2.0 mmol) and dissolved in DMF (7 mL) in a G30 microwave vial. Glacial acetic acid (100 µL) was then added. The resulting homogenous solution was placed in an Anton-Paar monowave 450 and heated as fast as possible to 100 °C. The solution was stirred at that temperature for 6 minutes. The resulting clear, slightly red solution was cooled to room temperature and the DMF was removed by rotary evaporation. The crude solid was redissolved in EtOAc (10 mL) and was washed with water (10 mL). The water layer was then

back extracted with EtOAc (3 × 5 mL). All organic layers were combined and then washed with 0.1 M HCl (10 mL) to remove unreacted starting material. The organic layer was separated, dried over Na_2SO_4, and concentrated under reduced pressure to yield 622 mg (98%) of **13** as a white, sticky foam. Analytical TLC, 9:1 CH_2Cl_2: MeOH eluent, R_f = 0.45. HRMS (ESI) m/z: $[M + H]^+$ calcd for $C_{31}H_{33}N_2O_7$ 545.2282; found 545.22761. IR (neat, cm^{-1}) 3324, 1708, 1179, 735. ^1H NMR (400 MHz, DMSO-d_6, ppm, 7:3 mixture of rotamers) δ 12.04 (s, 1 H), 7.88 (d, J = 7.5 Hz, 2 H), 7.67 (d, J = 7.5 Hz, 2 H), 7.42-7.3 (m, 9 H), 5.18 (s, 0.6 H), 5.13 (s, 1.4 H), 4.38-4.16 (m, 3.6 H), 4.06 (s, 1.4 H), 3.48-3.28 (m, 2 H), 3.22-3.04 (m, 2 H), 2.37 (t, J = 7.3 Hz, 1.4 H), 2.29-2.13 (m, 3.3 H), 1.76-1.63 (m, 2 H) . ^{13}C NMR (101 MHz, DMSO-d_6, ppm, mixture of rotamers) 174.3, 174.2, 174.1, 172.6, 172.3, 169.9, 169.4, 156.3, 156.2, 143.91, 143.86, 140.8, 140.7, 135.9, 135.7, 128.5, 128.4, 128.2, 128.0, 127.8, 127.6, 127.1, 125.1, 125.0, 120.12, 120.09, 66.4, 65.8, 65.4, 65.3, 49.8, 47.7, 47.6, 46.76, 46.72, 46.4, 38.3, 32.9, 32.8, 32.8, 31.3, 30.8, 20.3, 20.03, 19.98.

Compound 14:

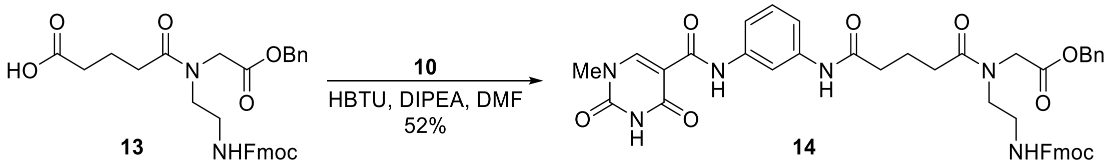

Benzyl *N*-(2-((((9*H*-fluoren-9-yl)methoxy)carbonyl)amino)ethyl)-*N*-(5-((3-(1-methyl-2,4-dioxo-1,2,3,4-tetrahydropyrimidine-5-carboxamido)phenyl)amino)-5-oxopentanoyl)glycinate: HBTU (219 mg, 0.576 mmol) and **13** (285 mg, 0.524 mmol) were dissolved in DMF (5 mL) and the clear light-yellow solution was stirred for 2 h. The solution was then cooled to 0 °C. Separately, **10** (150 mg, 0.576 mmol) was dissolved in DMF (3 mL) and was cooled to 0 °C. The solution of **10** was then transferred via syringe dropwise down the side of the flask containing **13** over approximately 5 minutes. DIPEA (137 μL, 0.786 mmol) was

then added down the flask side over 20 minutes and the reaction was passively warmed to room temperature and stirred for 22 h, at which point TLC showed full consumption of starting material. DMF was removed via rotary evaporation, and the resulting brown-red oil was redissolved in CH_2Cl_2 (75 mL). The organic solution was washed with water (60 mL), 20% citric acid solution (60 mL, pH 2), then brine (60 mL). The brine was rinsed with CH_2Cl_2 (3 × 75 mL). The organic layers were combined, dried (Na_2SO_4), and concentrated under reduced pressure to afford a yellow-brown solid that was purified by automated flash chromatography using a silica gel column (24 g, 35 mL/min) with a 0–20% gradient of MeOH in CH_2Cl_2 to afford 213 mg (52%) of **14** as a white powder. Analytical TLC, 9:1 CH_2Cl_2:MeOH eluent, R_f = 0.46. HRMS (ESI) m/z: $[M + H]^+$ calcd for $C_{43}H_{43}N_6O_9$ 787.3086; found 787.30830. IR (neat, cm^{-1}) 3044, 1678, 1608, 1540, 1416, 691. ^1H NMR (400 MHz, DMSO-d_6, ppm, 7:3 mixture of rotamers) δ 12.0 (s, 1 H), 10.9 (s, 1 H), 9.9 (s, 1 H), 8.6 (s, 1 H), 8.08 (m, 0.3 H), 7.8 (m, 2.69 H), 7.6 (s, 2 H), 7.49-7.17 (m, 13 H), 5.1 (s, 2 H), 4.5-4.1 (m, 3 H), 4.07 (s, 1 H), 3.4 (s, 4 H), 3.3 (s, 1H), 3.1 (s, 2 H), 2.4-2.1 (m, 4 H), 1.8 (s, 2 H). ^{13}C NMR (101 MHz, DMSO-d_6, ppm, mixture of rotamers) δ 172.6, 172.5, 171.1, 171.0, 169.9, 169.5, 164.3, 160.1, 156.3, 156.2, 152.7, 150.3, 143.90, 143.86, 140.8, 139.9, 138.4, 135.9, 135.6, 129.2, 128.5, 128.2, 128.1, 128.0, 127.9, 127.6, 127.1, 125.1, 125.0, 120.1, 114.6, 114.3, 110.2, 103.9, 66.4, 65.8, 65.4, 65.3, 49.9, 47.7, 46.7, 46.4, 36.3, 35.6, 35.5, 31.6, 31.0, 20.7, 20.5.

Compound 15 (*Db2*):

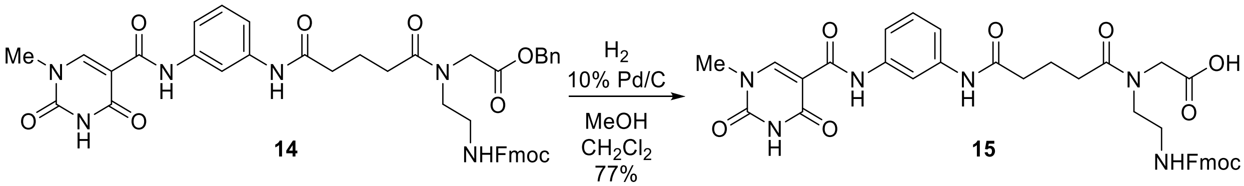

***N*-(2-((((9*H*-Fluoren-9-yl)methoxy)carbonyl)amino)ethyl)-*N*-(5-((3-(1-methyl-2,4-dioxo-1,2,3,4-tetrahydropyrimidine-5-carboxamido)phenyl)amino)-5-oxopentanoyl)glycine:**

Compound **14** (97 mg, 0.123 mmol) was dissolved in 20 mL of 10% MeOH:CH_2CL_2 with gentle heating and sonication until a clear, whitish solution was obtained. An N_2 atmosphere was established, upon which Pd/C (25 mg) was quickly added. The solution was placed under vacuum and backfilled with H_2 gas three times, and the solution was stirred overnight at room temperature. The cloudy mixture was then filtered through a celite pad to remove Pd/C followed by concentration under reduced pressure to afford 66 mg (77% yield) of **15** as a gray solid. HRMS (ESI) m/z: $[M + H]^+$ calcd for $C_{36}H_{37}N_6O_9$ 697.2617; found 697.26068. IR (neat, cm^{-1}) 3044, 1678, 1608, 1540, 1416, 691.^{1}H NMR (400 MHz, DMSO-*d*$_6$, ppm, 7:3 mixture of rotamers) δ 12.1 (br s, 1 H), 10.9 (s, 1 H), 9.9 (s, 1 H), 8.6 (d, J = 3.1 Hz, 1 H), 7.9-7.8 (m, 3 H), 7.65 (t, J = 8.1 Hz, 2 H), 7.42-7.28 (m, 7 H), 4.33-4.24 (m, 2 H), 4.2 (t, J = 6.47, 2 H), 4.0 (s, 0.7 H), 3.92 (s, 1.3 H), 3.41 (s, 3 H), 2.4-2.2 (m, 4 H), 1.23 (s, 2 H). ^{13}C NMR (101 MHz, DMSO-*d*$_6$, ppm, mixture of rotamers) δ 172.6, 172.2, 171.0, 164.3, 160.1, 156.2, 156.2, 152.7, 150.4, 143.9, 143.9, 140.7, 139.9, 138.4, 129.2, 128.5, 128.1, 127.9, 127.6, 127.1, 125.2, 125.1, 120.1, 114.6, 114.2, 110.2, 103.9, 65.4, 47.5, 47.3, 46.7, 36.3, 35.6, 31.6, 31.0, 20.7, 20.5.

Experimental for Synthesis of ***Db3***

Compound 17:

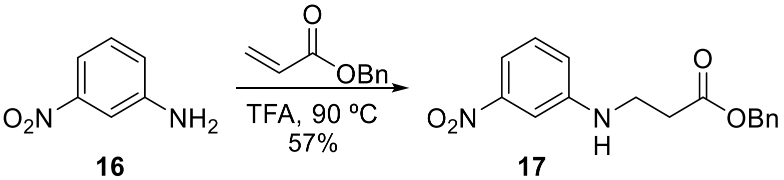

Benzyl 3-((3-nitrophenyl)amino)propanoate: TFA (3 drops) was added to a suspension of 3-nitroaniline (4.39 g, 31.8 mmol) in benzyl acrylate (9.5 mL) at room temperature whereupon the solution was heated to 200 °C under N_2 atmosphere and stirred overnight. The solution was then cooled to room temperature and concentrated under reduced pressure. The residue was redissolved in $CHCl_3$:iPrOH (3:1) (75 mL) and the organic layer was washed with H_2O (3 × 50 mL), sat. $NaHCO_3$ (50 mL). The organic layer was combined, dried ($MgSO_4$), and concentrated under reduced pressure. The crude product was purified via column chromatography (SiO_2) eluting with a hexanes/EtOAc (70:30 → 60:40 → 50:50) to afford 1.86 g (57%) of **17** as an orange oil. Analytical TLC, 50:50 hexanes/EtOAc eluent, R_f = 0.51. HRMS (ESI) m/z: $[M + H]^+$ calcd for $C_{16}H_{17}N_2O_4$ 301.1183; found 301.11812. IR (neat, cm^{-1}) 3402, 3089, 1726, 1525, 1343, 1183. ^1H NMR (400 MHz, DMSO-d_6, ppm) δ 7.40 – 7.25 (m, 7 H), 7.00 (dt, J = 7.05, 2.21 Hz, 1 H), 6.47 (t, J = 5.67 Hz, 1 H), 5.11 (s, 2 H), 3.39 (q, J = 6.38 Hz, 2 H), 2.67 (t, J = 6.61 Hz, 2 H). ^{13}C NMR (101 MHz, DMSO-d_6, ppm) δ 171.3, 149.5, 148.9, 136.1, 130.0, 128.4, 128.0, 127.9, 118.2, 110.0, 105.3, 65.6, 38.5, 33.4.

Compound 18:

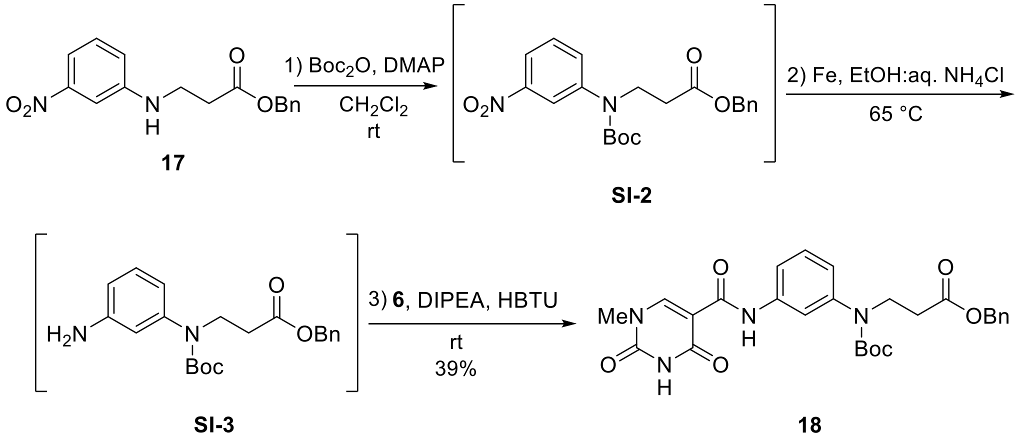

Benzyl 3-((*tert*-butoxycarbonyl)(3-(1-methyl-2,4-dioxo-1,2,3,4-tetrahydropyrimidine-5-carboxamido)phenyl)amino)propanoate: Boc_2O (4.96 g, 22.7 mmol) was added to a solution of **17** (3.41 g, 11.4 mmol) and DMAP (694 mg, 5.68 mmol) in CH_2Cl_2 (57 mL) at room temperature. The solution was stirred overnight upon which the organic solution was washed with sat. $CuSO_4$ (50 mL), H_2O (2 × 50 mL), and sat. $NaHCO_3$ (50 mL). The organic layer was collected, dried ($MgSO_4$), and concentrated under reduced pressure. The crude product, **SI-2**, was directly taken to the next reaction.

Fe (908 mg, 16.3 mmol) was added to a solution of **SI-2** (3.25 g, 8.13 mmol) in 1:1 EtOH:aq. NH_4Cl (41 mL) at room temperature. The solution was heated to 60 °C and stirred for 12 h. The solution was cooled to room temperature and was then filtered through celite. The celite was washed with EtOH (150 mL) and H_2O (150 mL) and the solution was concentrated under reduced pressure. The aqueous layer was extracted with EtOAc (3 × 150 mL) and the organic layers were combined, dried ($MgSO_4$) and concentrated under reduced pressure. The crude product, **SI-3**, was directly taken to the next reaction.

DIPEA (3.15 mg, 24.4 mmol, 4.2 mL) was added to a solution of **6** (2.08 g, 12.2 mmol) in anhydrous NMP (30 mL) at room temperature and was stirred for 30 minutes. HBTU (5.08 g, 13.4 mmol) was then added to the reaction which was stirred for 1.5 h whereupon a solution of **SI-3** (4.51 g, 12.2 mmol) was added. The solution was stirred at room temperature overnight and was then concentrated under reduced pressure. The crude oil was redissolved in CH_2Cl_2 (100 mL) and was washed sequentially with H_2O (3 × 50 mL), sat. $NaHCO_3$ (25 mL) and brine (25 mL). The organic layer was collected, dried ($MgSO_4$), and concentrated under reduced pressure. The crude product was purified via column chromatography (SiO_2) eluting with a hexanes:EtOAc (50:50 → 25:75 → 0:100) gradient to afford 2.49 g (39% over 3 steps) of **18** as a pale brown solid. HRMS (ESI) m/z: $[M - H]^-$ calcd for $C_{27}H_{29}N_4O_7$ 521.2042; found 521.20464. IR (neat, cm^{-1}) 3048, 1730, 1677, 1602, 1451, 1155. ^{1}H NMR (400 MHz, DMSO-d_6, ppm) δ 12.10 – 12.00 (br s, 1 H), 10.97 (s, 1 H), 8.64 (s, 1 H), 7.59 (t, *J* = 2.08 Hz, 1 H), 7.50 – 7.42 (br d, 1 H), 7.38 – 7.26 (m, 6 H), 6.97 – 6.92 (m, 1 H), 5.00 (s, 2 H), 3.87 (t, *J* = 7.01 Hz, 2 H), 3.42 (s, 3 H), 2.58 (t, *J* = 7.01 Hz, 2 H), 1.36 (s, 9 H). ^{13}C NMR (101 MHz, DMSO-d_6, ppm) δ 171.2, 164.6, 160.8, 153.9, 153.2, 150.8, 142.8, 138.9, 136.4, 129.6, 128.8, 128.4, 128.4, 122.6, 118.9, 117.8, 104.2, 80.3, 66.1, 46.1, 36.7, 33.5, 28.3, 20.5.

Compound 19:

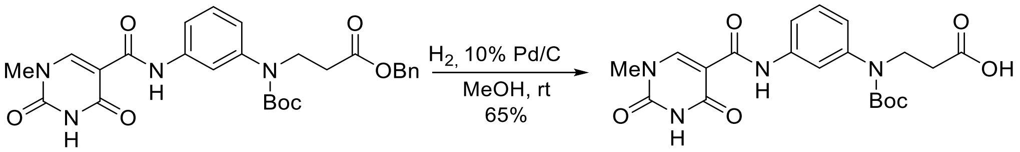

18 **19**

3-((*tert*-Butoxycarbonyl)(3-(1-methyl-2,4-dioxo-1,2,3,4-tetrahydropyrimidine-5-carboxamido)phenyl)amino)propanoic acid. A solution of **18** (483 mg, 0.925 mmol) and 10% Pd/C (98.4 mg, 0.092 mmol) in MeOH (12 mL) was backfilled with H_2 gas three times at room temperature whereupon the solution was stirred at room temperature overnight. The solution was then filtered through celite and the celite was washed with $CHCl_3$:iPrOH (3:1) (100 mL). The filtrate was then dried ($MgSO_4$), filtered and concentrated under reduced pressure to afford 260 mg (65%) of **19** as an off white solid. HRMS (ESI) m/z: $[M - H]^-$ calcd for $C_{20}H_{23}N_4O_7$ 431.1572; found 431.15756. IR (neat, cm^{-1}) 2972, 1688, 1599, 1364, 1146, 1057. ^1H NMR (400 MHz, DMSO-d_6, ppm) δ 12.22 – 11.95 (br s, 1 H), 10.98 (s, 1 H), 8.65 (s, 1 H), 7.59 (t, *J* = 2.07 Hz, 1 H), 7.48 – 7.41 (br d, 1 H), 7.32 (t, *J* = 8.04 Hz, 1 H), 7.00 – 6.93 (m, 1 H), 3.80 (t, *J* = 7.33 Hz, 2 H), 3.42 (s, 3 H), 2.43 (t, *J* = 7.33 Hz, 2 H), 1.38 (s, 9 H). ^{13}C NMR (101 MHz, DMSO-d_6, ppm) δ 164.1, 160.3, 153.4, 152.8, 150.3, 142.5, 138.4, 129.1, 122.0, 118.3, 117.2, 103.8, 79.7, 45.7 (HSQC), 32.9 (HSQC), 36.3, 27.9.

Compound 20:

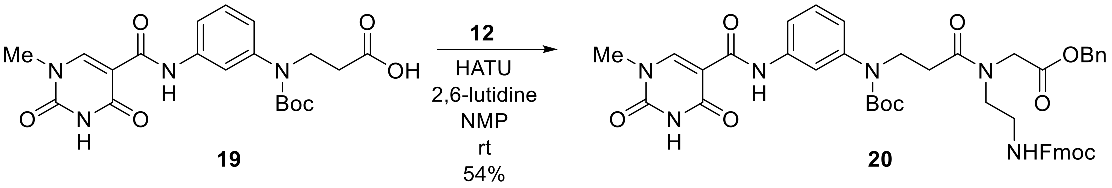

Benzyl *N*-(2-((((9*H*-fluoren-9-yl)methoxy)carbonyl)amino)ethyl)-*N*-(3-((tert-butoxycarbonyl)(3-(1-methyl-2,4-dioxo-1,2,3,4-tetrahydropyrimidine-5-carboxamido)phenyl)amino)propanoyl)glycinate. DIPEA (475 mg, 3.67 mmol, 0.64 mL) was added to a solution of **19** (794 mg, 1.84 mmol) in NMP (24.5 mL) at room temperature. The solution was stirred for three hours whereupon HATU (1.05 mg, 2.76 mmol) was added and stirred for an additional three hours. Next, **12** [1] (753 mg, 1.75 mmol) was added, and the reaction was stirred at room temperature overnight. Upon LCMS conformation of conversion, the reaction was diluted using a solution of 3:1 $CHCl_3$:iPrOH (100 mL) and the organic solution was washed with H_2O (3 × 50 mL) and sat. $NaHCO_3$ (25 mL). The organic layer was collected, dried ($MgSO_4$), and concentrated under reduced pressure. The crude product was purified via column chromatography (SiO_2) eluting with hexanes:EtOAc (50:50 → 25:75 → 0:100) to afford 273 mg (54%) of **20** as a foamy pale yellow solid. Analytical TLC, 25:75 hexanes/EtOAc eluent, $R_f = 0.41$. HRMS (ESI) m/z: $[M + H]^+$ calcd for $C_{46}H_{49}N_6O_{10}$ 845.3505; found 845.34976. IR (neat, cm^{-1}) 3063, 1688, 1645, 1598, 1448, 1247, 1161. ^1H NMR (400 MHz, DMSO-d_6, ppm, as a 40:60 mixture of rotamers) δ 12.03 (s, 1 H), 10.97 (d, *J* = 7.02 Hz, 1 H), 8.61 (s, 0.4 H), 8.59 (s, 0.6 H), 7.91 – 7.81 (m, 2 H), 7.71 – 7.55 (m, 3 H), 7.47 – 7.14 (m, 12 H), 6.94 (d, *J* = 7.84 Hz, 1 H), 5.13 (s, 0.8 H), 5.10 (s, 1.2 H), 4.32 – 3.98 (m, 5 H), 3.73 (t, *J* = 7.74 Hz, 2 H), 3.43 – 3.35 (m, 4 H), 3.10 (dd, *J* = 24.42, 6.33 Hz, 2 H), 2.63 (t, *J* = 7.80 Hz, 1 H), 1.36 (d, *J* = 2.88 Hz, 9 H). ^{13}C NMR (101 MHz, ppm, as a mixture of rotamers) δ 171.4, 171.2, 169.7, 164.6, 160.7,

156.6, 153.9, 153.2, 150.7, 144.3, 143.2, 141.2, 138.8, 136.3, 129.5, 128.9, 128.9, 128.6, 128.5, 128.4, 128.3, 128.0, 127.5, 125.4, 120.5, 118.7, 117.6, 104.2, 80.2, 66.9, 66.3, 65.8, 48.9, 47.2, 36.7, 30.5, 29.4, 28.3, 17.7.

Compound 21 *(Db3)*:

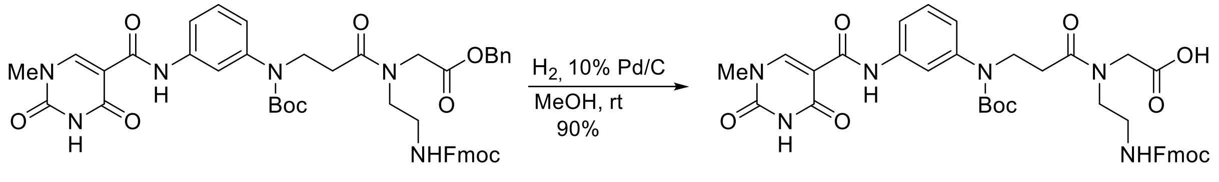

***N*-(2-((((9*H*-Fluoren-9-yl)methoxy)carbonyl)amino)ethyl)-*N*-(3-((*tert*-butoxycarbonyl)(3-(1-methyl-2,4-dioxo-1,2,3,4-tetrahydropyrimidine-5-carboxamido)phenyl)amino)propanoyl)glycine.** A solution of **20** (273 mg, 0.324 mmol) and 10% Pd/C (48.0 mg, 0.045 mmol) in a 3:1 mix of MeOH:THF (12 mL) was backfilled with H_2 gas three times at room temperature whereupon the solution was stirred at room temperature overnight. The solution was then filtered through celite and the celite was washed with MeOH (50 mL). The filtrate was then dried ($MgSO_4$), filtered and concentrated under reduced pressure to afford 220 mg (90%) of **21** as an off white solid. HRMS (ESI) m/z: $[M + H]^+$ calcd for $C_{39}H_{43}N_6O_{10}$ 755.3035; found 755.30275. IR (neat, cm^{-1}) 3192, 3067, 2970, 1689, 1651, 1602, 1448, 1252, 1061. ^1H NMR (400 MHz, DMSO-d_6, ppm, as a 40:60 mixture of rotamers) δ 12.21 – 11.84 (br s, 1 H), 10.96 (s, 0.4 H), 10.95 (s, 0.6 H), 8.63 (s, 0.4 H), 8.60 (s, 0.6 H), 7.92 – 7.82 (m, 2 H), 7.69 – 7.55 (m, 3 H), 7.47 – 7.18 (m, 8 H), 6.99 – 6.91 (m, 1 H), 4.31 – 4.03 (m, 4 H), 3.89 (s, 1 H), 3.79 – 3.70 (m, 2 H), 3.60 (dddd, *J* = 7.20, 5.11, 2.55, 1.29 Hz, 1 H), 3.41 (s, 1.2 H), 3.40 (s, 1.8 H), 3.10 (dd, *J* = 24.00, 6.39 Hz, 2 H), 2.68 – 2.56 (m, 1 H), 1.79 – 1.72 (m, 1 H), 1.37 (d, *J* = 1.94 Hz, 9 H). ^{13}C NMR (101 MHz, DMSO-d_6, ppm, as a mixture of rotamers) δ

171.8 (HMBC), 171.3 (HMBC), 164.1, 160.2, 156.1, 153.5, 152.8, 150.3, 148.6 (HMBC), 143.8, 142.6 (HMBC), 140.7, 138.4, 129.1 (HMBC), 127.6, 127.0, 125.0, 124.2, 121.8 (HMBC), 120.1, 119.9, 119.0 (HMBC), 118.2 (HMBC), 117.1 (HMBQ), 103.8, 79.7, 67.0, 65.4, 46.7, 36.3, 27.9, 27.9, 25.1, 20.1.

Experimental for Synthesis of ***Db3*** *extinction coefficient molecule:*

Compound SI-4:

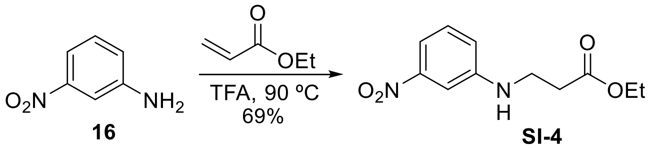

Ethyl 3-((3-nitrophenyl)amino)propanoate: Following a literature precedent [4], TFA (3 drops) was added to a suspension of 3-nitroaniline (3.73 g, 27.0 mmol) in ethyl acrylate (8 mL) at room temperature whereupon the solution was heated to 90 °C and stirred overnight. The solution was cooled to room temperature and then concentrated under reduced pressure. The crude product was purified via column chromatography (SiO_2) eluting with a hexanes/ethyl acetate/Et_3N (85:14:1 → 80:19:1) to afford 4.46 g (69%) of **SI-4** as an orange oil. Analytical TLC, 85:14:1 hexanes/ethyl acetate/Et_3N eluent, R_f = 0.21. IR (neat, cm^{-1}) 3381, 2982, 1710, 1529, 1345, 1196. ^1H NMR (400 MHz, DMSO-d_6, ppm) δ 7.38 – 7.28 (m, 3 H), 7.02 – 6.95 (m, 1 H), 6.50 - 6.37 (br s, 1 H), 4.07 (q, J = 7.08 Hz, 2 H), 3.40 – 3.29 (m, 2 H), 2.58 (t, J = 6.61 Hz, 2 H), 1.18 (t, J = 7.08 Hz, 3 H). ^{13}C NMR (101 MHz, DMSO-d_6, ppm) δ 171.4, 149.5, 148.9, 130.0, 118.2, 109.9, 105.2, 60.0, 38.5, 33.4, 14.0.

Compound SI-5:

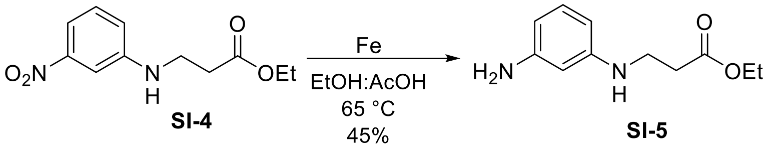

Ethyl 3-((3-aminophenyl)amino)propanoate: Fe (2.61 g, 46.8 mmol) was added to a solution of **SI-4** (4.46 g, 18.7 mmol) in EtOH (130 mL) and AcOH (57 mL). The solution was heated to 65 °C and was stirred overnight. The solution was let cool to room temperature and was concentrated under reduced pressure. The residue was cooled to 0 °C and was treated with sat. $NaHCO_3$ (170 mL) until pH 7. The aqueous layer was extracted with CH_2Cl_2 (3 x 150 mL) and the organic layers were combined, dried ($MgSO_4$), and concentrated under reduced pressure to afford 1.77 g (45%) of **SI-5** as a brown oil. Analytical TLC, 80:20 hexanes/ethyl acetate eluent, R_f = 0.09. IR (neat, cm^{-1}) 3360, 2977, 1719, 1178. ^1H NMR (400 MHz, $CDCl_3$, ppm) δ 6.96 (t, J = 7.93 Hz, 1 H), 6.07 (td, J = 7.53, 2.18 Hz, 2 H), 5.97 (t, J = 2.20 Hz, 1 H), 4.15 (q, J = 7.11 Hz, 2 H), 3.41 (t, J = 6.38 Hz, 2 H), 2.59 (t, J = 6.37 Hz, 2 H), 1.26 (t, J = 7.14 Hz, 3 H). ^{13}C NMR (101 MHz, $CDCl_3$, ppm) δ 172.6, 148.9, 147.7, 130.3, 105.3, 104.3, 99.9, 60.7, 39.5, 34.1, 14.3.

Compound SI-6:

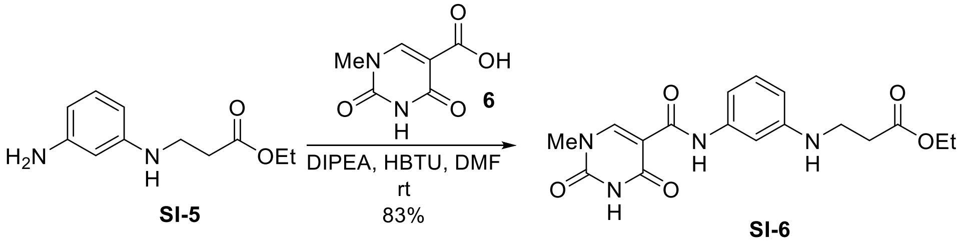

Ethyl 3-((3-(1-methyl-2,4-dioxo-1,2,3,4-tetrahydropyrimidine-5-carboxamido)phenyl)amino)propanoate: DIPEA (937 mg, 7.25 mmol, 1.3 mL) was added to a solution of **6** (617 mg, 3.63 mmol) in DMF (9.1 mL) at room temperature and was stirred for 30

minutes. HBTU (1.51 g, 3.99 mmol) was added to the reaction and was stirred for 4 h whereupon a solution of **SI-5** (755 mg, 3.63 mmol) in DMF (9.1 mL) was added to the reaction via syringe. The solution stirred at room temperature overnight whereupon the solution was concentrated under reduced pressure. The residue was redissolved in $CHCl_3$:iPrOH (3:1) (100 mL) and the organic layer was washed sequentially with H_2O (3 x 20 mL) then sat. $NaHCO_3$ (25 mL). The organic layer was collected, dried ($MgSO_4$), and concentrated under reduced pressure. The crude product was purified via column chromatography (SiO_2) eluting with a EtOAc:MeOH gradient (95:5 → 90:10) to afford 1.71 g (83%) of **SI-6** as a pale green solid. Analytical TLC, 95:5 EtOAc/MeOH eluent, R_f = 0.42. IR (neat, cm^{-1}) 3384, 3042, 1713, 1672, 1609, 1368, 1181. ^1H NMR (400 MHz, DMSO-d_6, ppm) δ 12.02 (s, 1 H), 10.77 (s, 1 H), 8.60 (s, 1 H), 7.03 (t, *J* = 8.02 Hz, 1 H), 6.97 – 6.91 (m, 1 H), 6.81 – 6.73 (m, 1 H), 6.35 – 6.28 (m, 1 H), 5.74 (t, *J* = 5.77 Hz, 1 H), 4.07 (q, *J* = 7.10 Hz, 2 H), 3.41 (s, 3 H), 3.26 (q, *J* = 6.26 Hz, 2 H), 2.56 (t, *J* = 6.76 Hz, 2 H), 1.19 (t, *J* = 7.12 Hz, 3 H). ^{13}C NMR (101 MHz, DMSO-d_6, ppm) δ 171.6, 164.2, 162.3, 159.9, 152.5, 150.3, 149.1, 138.9, 129.4, 107.5, 104.1, 103.2, 59.9, 38.2, 36.3, 33.6, 14.1.

NMR Spectra for synthetic intermediates and monomers

Compound 3

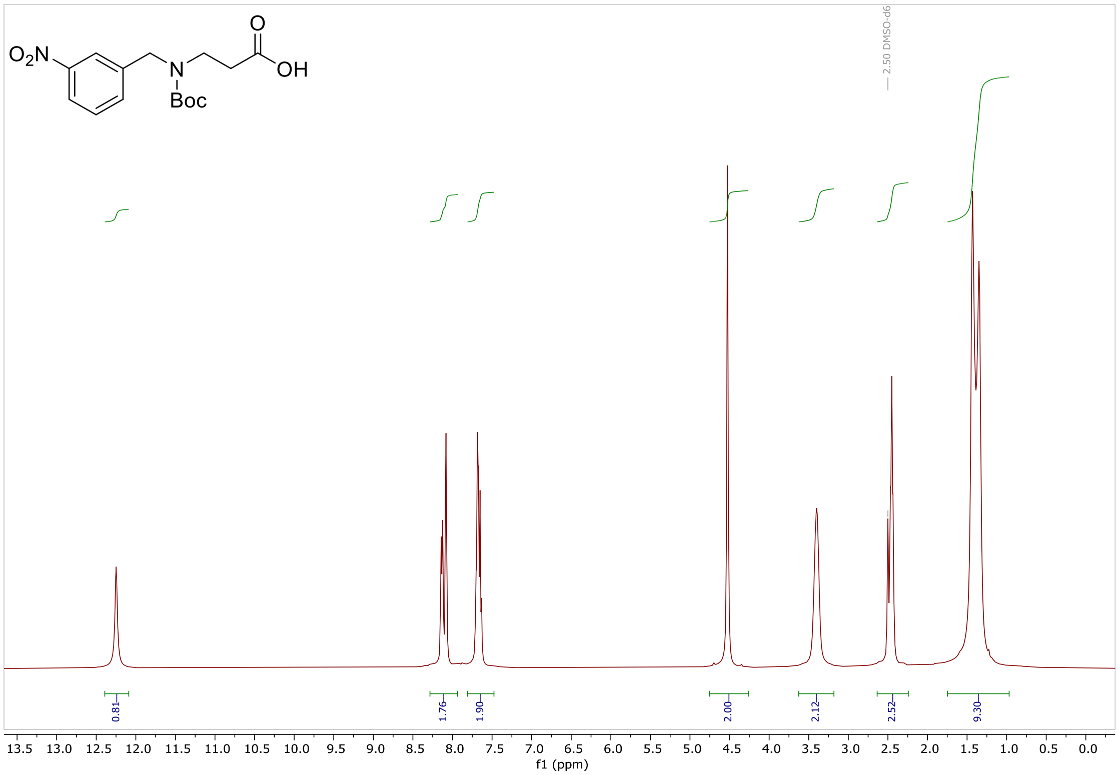

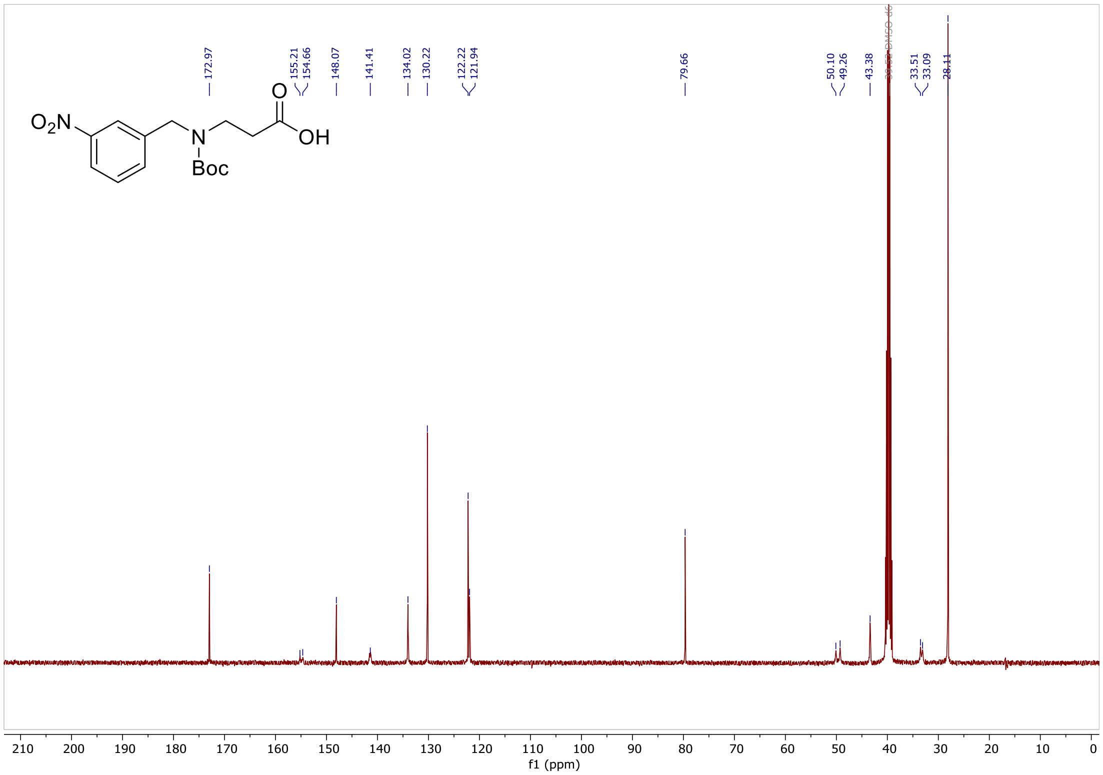

Compound 5

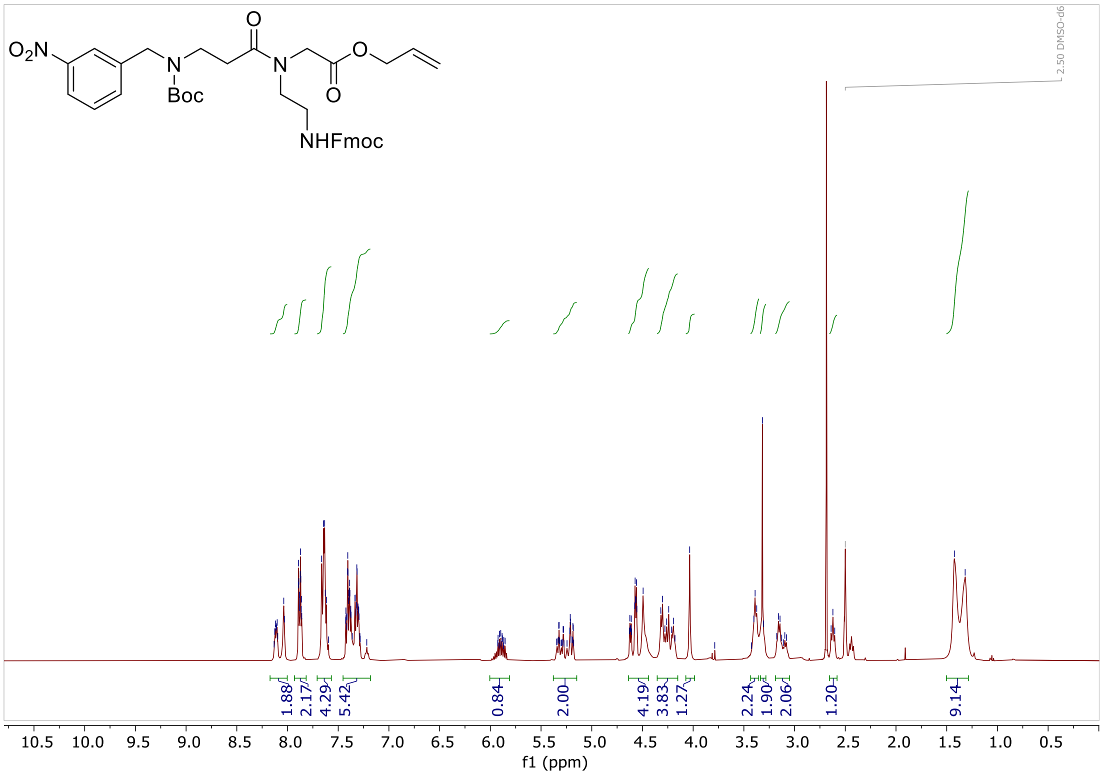

O2N
N
Boc
O
N
O
O
NHFmoc
2.50 DMSO-d6
1.88
2.17
4.29
5.42
0.84
2.00
4.19
3.83
1.27
2.24
1.90
2.06
1.20
9.14
10.5 10.0 9.5 9.0 8.5 8.0 7.5 7.0 6.5 6.0 5.5 5.0 4.5 4.0 3.5 3.0 2.5 2.0 1.5 1.0 0.5
f1 (ppm)

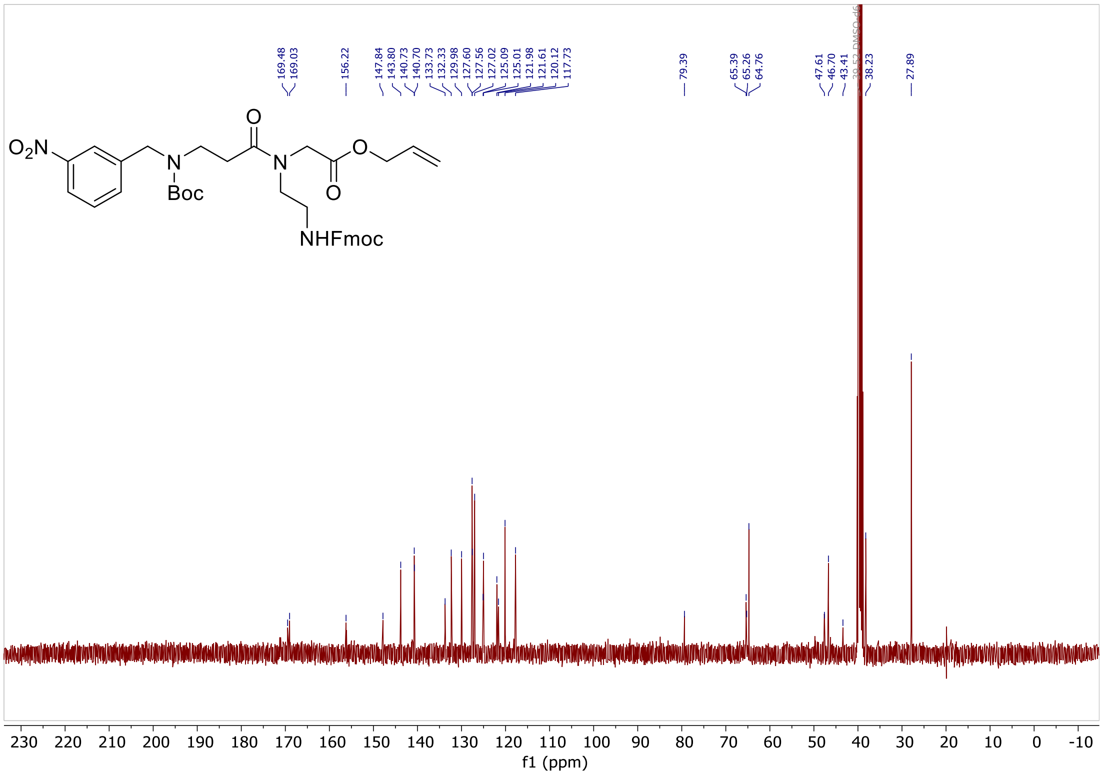

169.48
169.03
156.22
147.84
143.80
140.73
140.70
133.73
132.33
129.98
127.60
127.56
127.02
125.09
125.01
121.98
121.61
120.12
117.73
79.39
65.39
65.26
64.76
47.61
46.70
43.41
38.23
27.89
O2N
N
Boc
O
N
O
O
NHFmoc
230 220 210 200 190 180 170 160 150 140 130 120 110 100 90 80 70 60 50 40 30 20 10 0 -10
f1 (ppm)

Compound 7

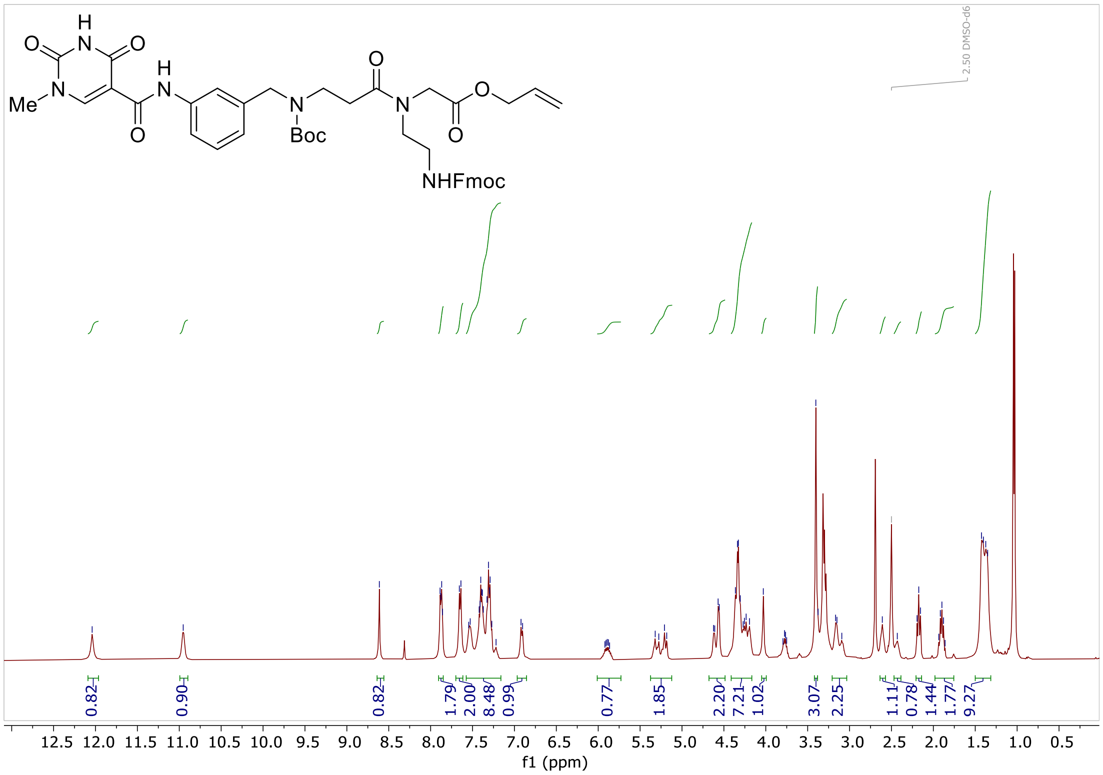
Me
Boc
NHFmoc
2.50 DMSO-d6
0.82
0.90
0.82
1.79
2.00
8.48
0.99
0.77
1.85
2.20
7.21
1.02
3.07
2.25
1.11
0.78
1.44
1.77
9.27
12.5 12.0 11.5 11.0 10.5 10.0 9.5 9.0 8.5 8.0 7.5 7.0 6.5 6.0 5.5 5.0 4.5 4.0 3.5 3.0 2.5 2.0 1.5 1.0 0.5
f1 (ppm)

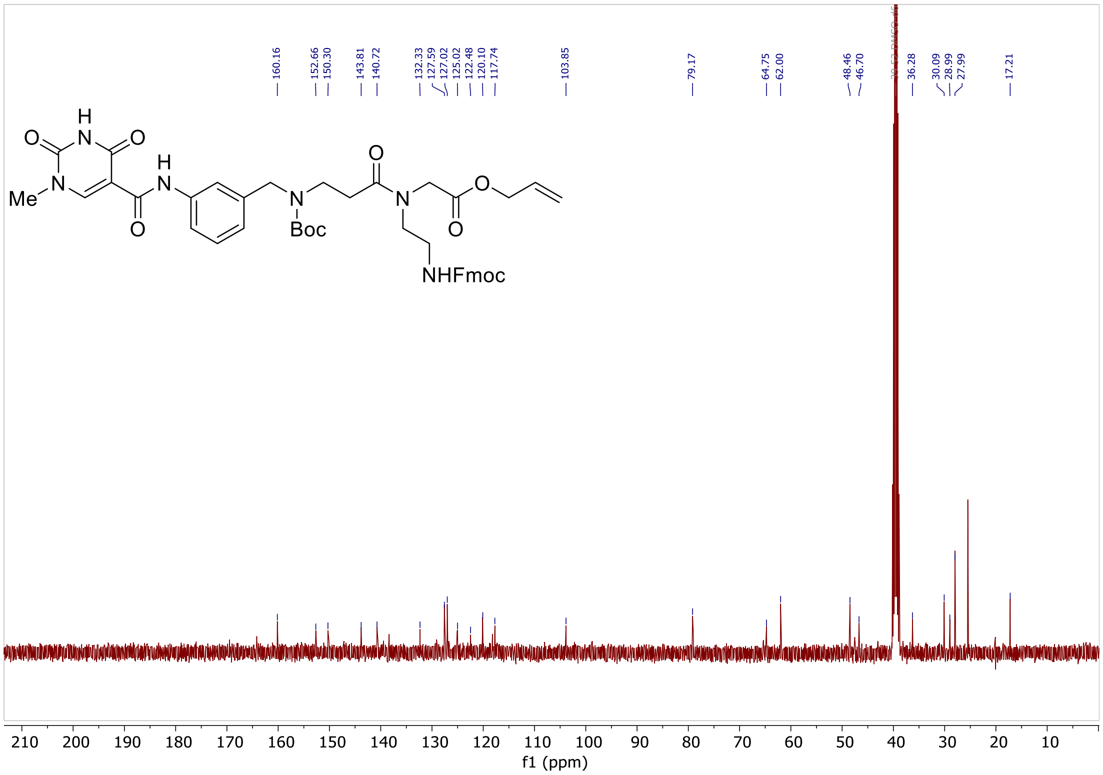
160.16
152.66
150.30
143.81
140.72
132.33
127.59
127.02
125.02
122.48
120.10
117.74
103.85
79.17
64.75
62.00
48.46
46.70
36.28
30.09
28.99
27.99
17.21
Me
Boc
NHFmoc
210 200 190 180 170 160 150 140 130 120 110 100 90 80 70 60 50 40 30 20 10
f1 (ppm)

Compound 8

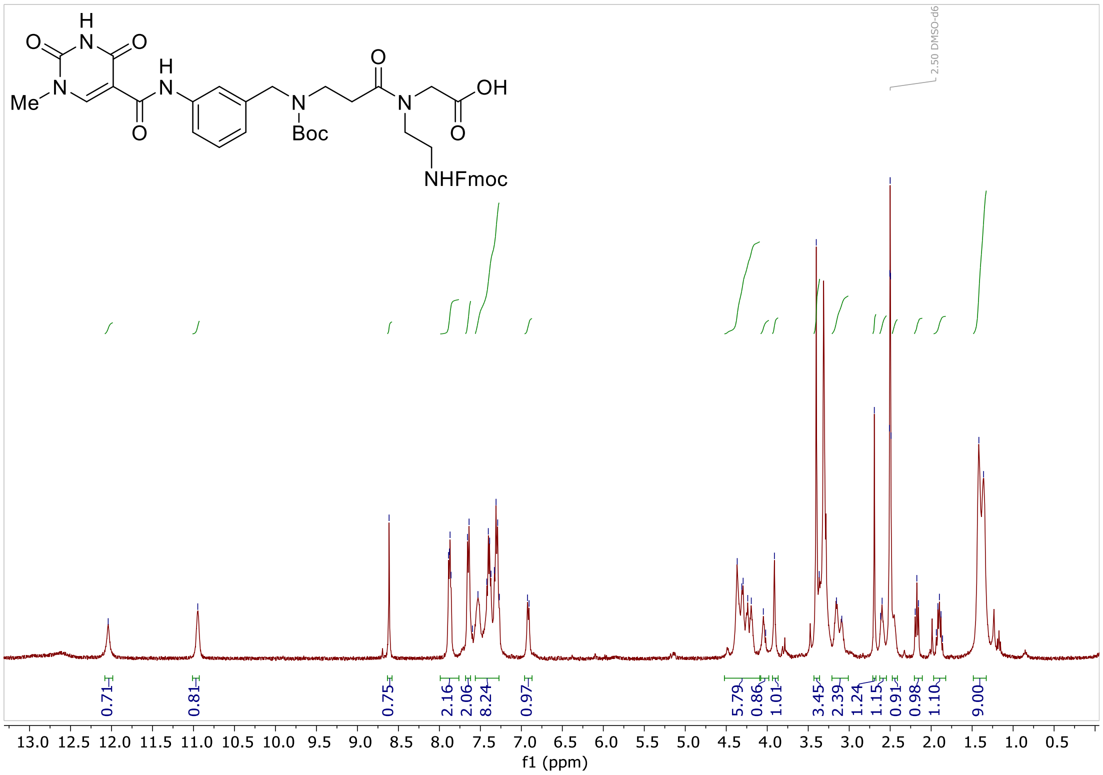

2.50 DMSO-d6
0.71
0.81
0.75
2.16
2.06
8.24
0.97
5.79
0.86
1.01
3.45
2.39
1.24
1.15
0.91
0.98
1.10
9.00
13.0 12.5 12.0 11.5 11.0 10.5 10.0 9.5 9.0 8.5 8.0 7.5 7.0 6.5 6.0 5.5 5.0 4.5 4.0 3.5 3.0 2.5 2.0 1.5 1.0 0.5
f1 (ppm)

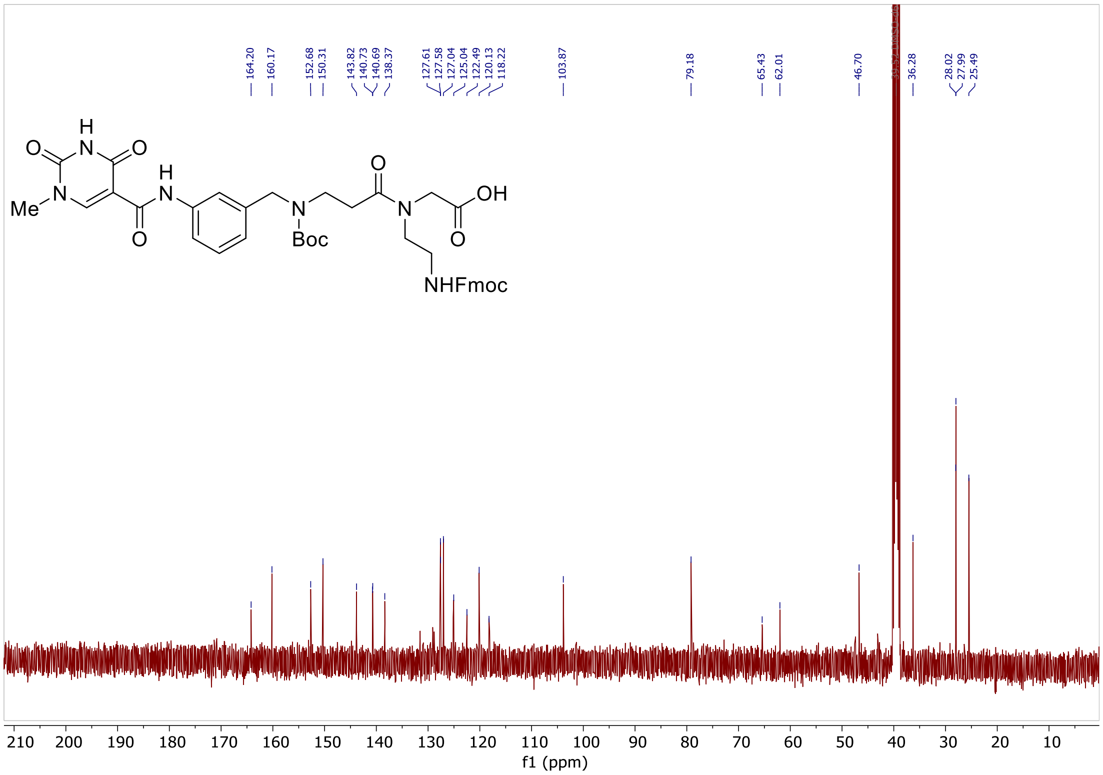

164.20
160.17
152.68
150.31
143.82
140.73
140.69
138.37
127.61
127.58
127.04
125.04
122.49
120.13
118.22
103.87
79.18
65.43
62.01
46.70
36.28
28.02
27.99
25.49
210 200 190 180 170 160 150 140 130 120 110 100 90 80 70 60 50 40 30 20 10
f1 (ppm)

Compound SI-1

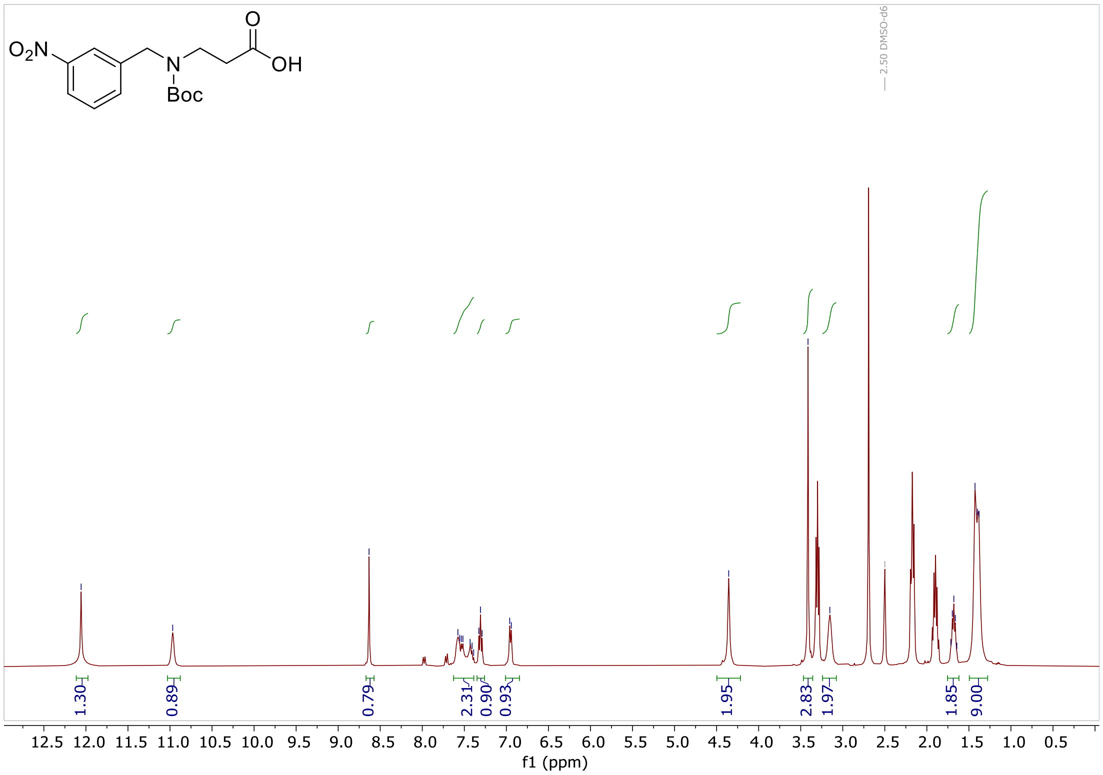

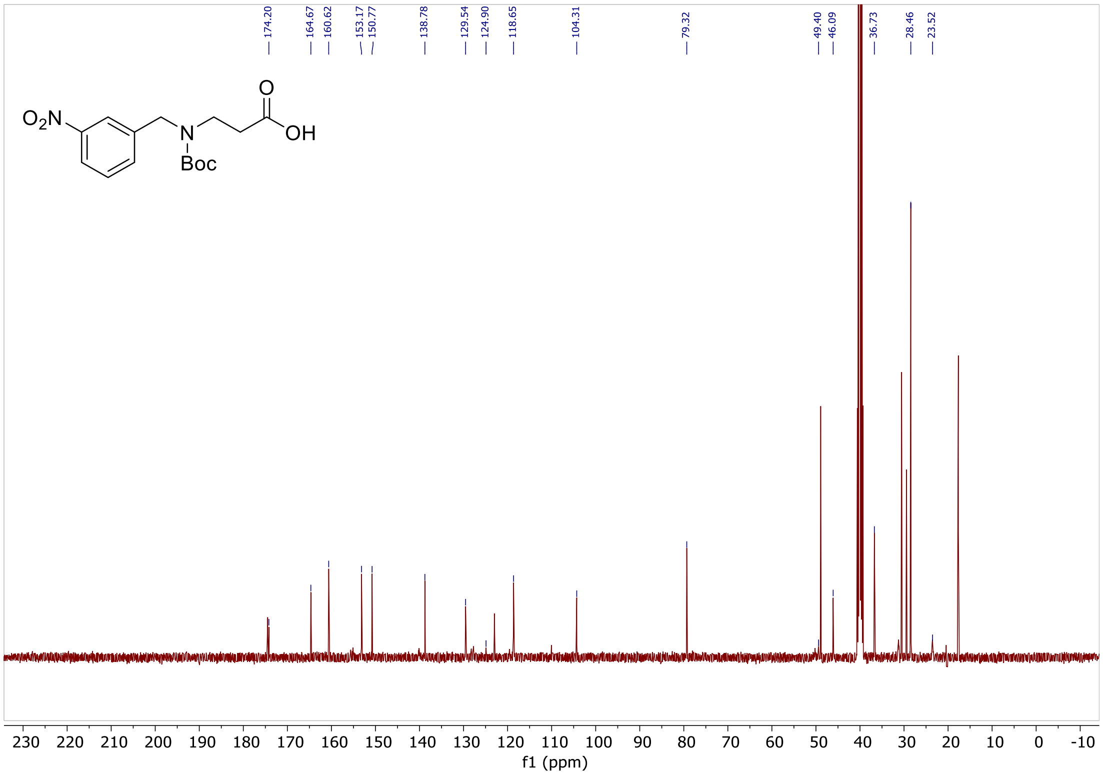

Compound 10

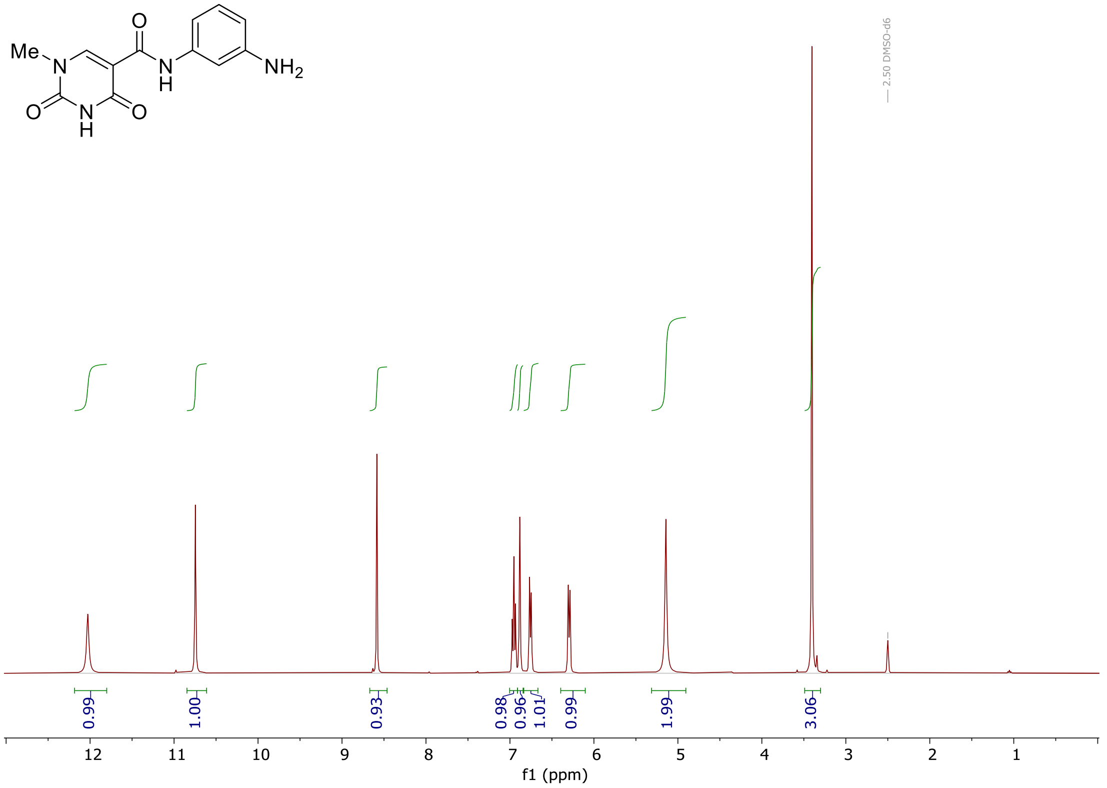
Me
N
O
N
H
O
O
N
H
NH2
2.50 DMSO-d6
0.99
1.00
0.93
0.98
0.96
1.01
0.99
1.99
3.06
12 11 10 9 8 7 6 5 4 3 2 1
f1 (ppm)

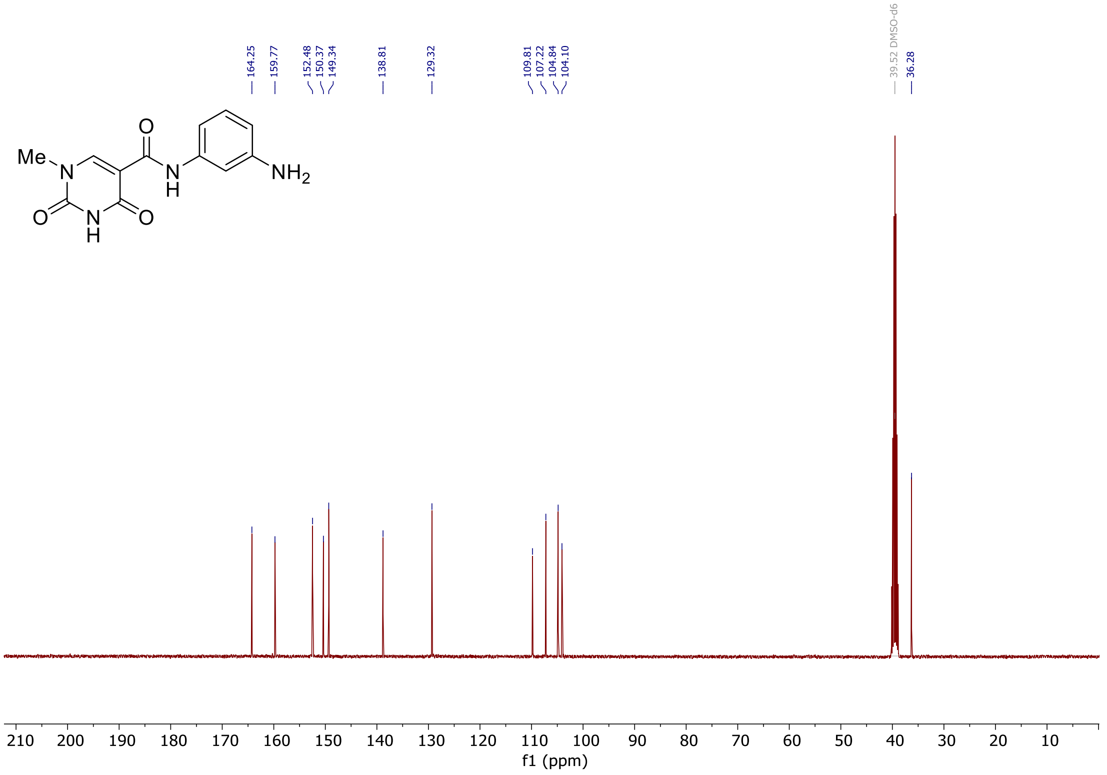
164.25
159.77
152.48
150.37
149.34
138.81
129.32
109.81
107.22
104.84
104.10
39.52 DMSO-d6
36.28
Me
N
O
N
H
O
O
N
H
NH2
210 200 190 180 170 160 150 140 130 120 110 100 90 80 70 60 50 40 30 20 10
f1 (ppm)

Compound 13

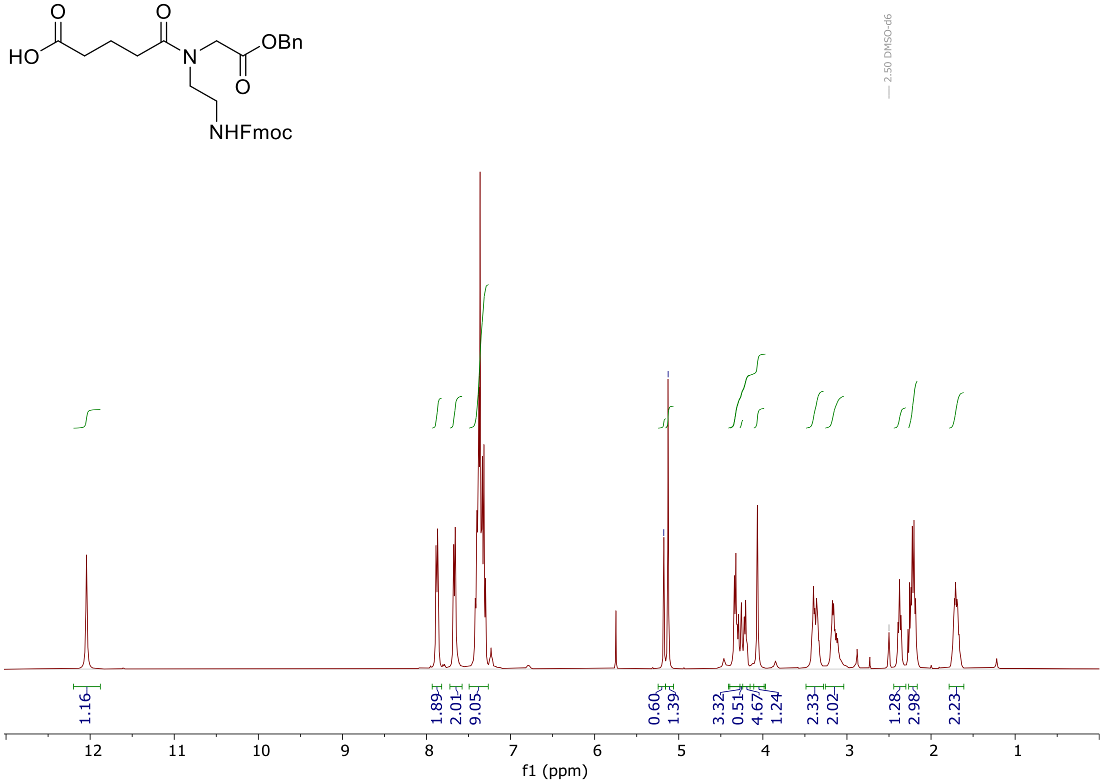

HO
O
O
N
OBn
O
NHFmoc
2.50 DMSO-d6
1.16
1.89
2.01
9.05
0.60
1.39
3.32
0.51
4.67
1.24
2.33
2.02
1.28
2.98
2.23
12 11 10 9 8 7 6 5 4 3 2 1
f1 (ppm)

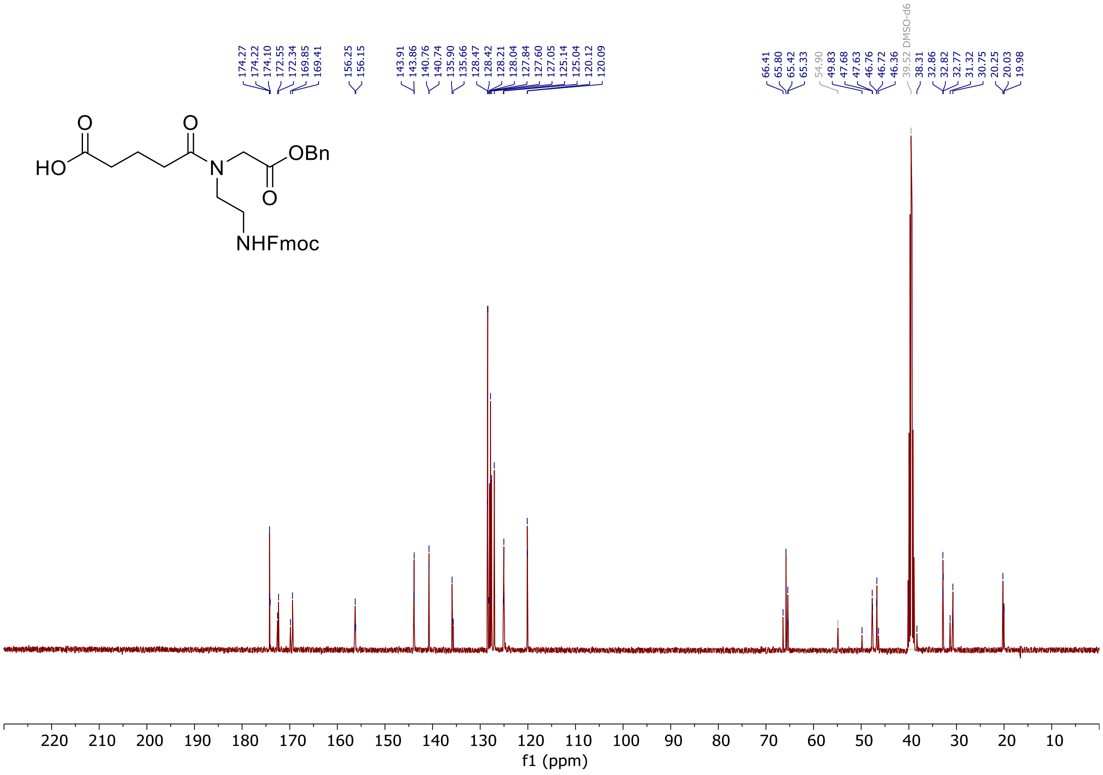

174.27
174.22
174.10
172.55
172.34
169.85
169.41
156.25
156.15
143.91
143.86
140.76
140.74
135.90
135.66
128.47
128.42
128.21
128.04
127.84
127.60
127.05
125.14
125.04
120.12
120.09
66.41
65.80
65.42
65.33
54.90
49.83
47.68
47.63
46.76
46.72
46.36
39.52 DMSO-d6
38.31
32.86
32.82
32.77
31.32
30.75
20.25
20.03
19.98
HO
O
O
N
OBn
O
NHFmoc
220 210 200 190 180 170 160 150 140 130 120 110 100 90 80 70 60 50 40 30 20 10
f1 (ppm)

Compound 14

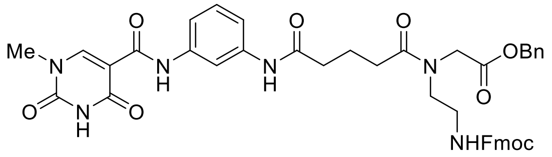
Me
N
O
N
H
O
O
N
H
O
N
H
O
O
N
OBn
O
NHFmoc

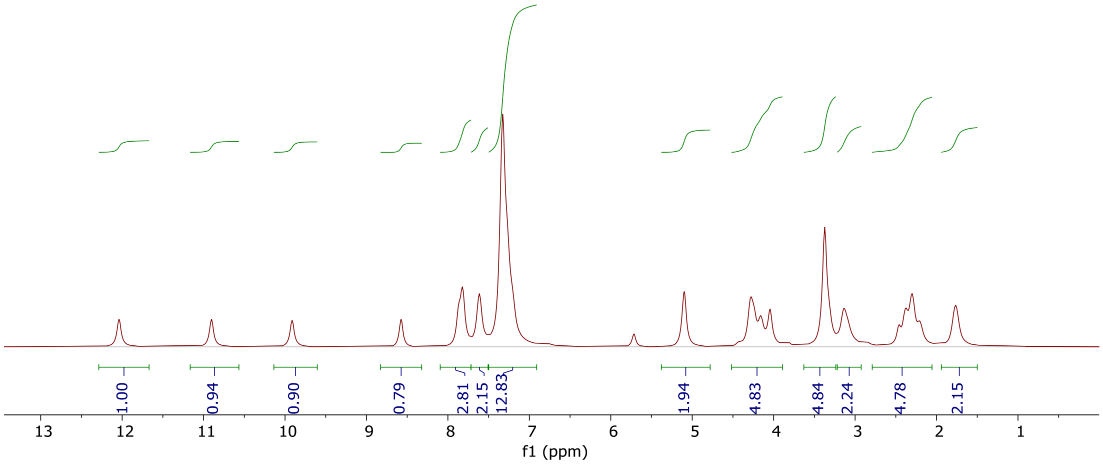
1.00
0.94
0.90
0.79
2.81
2.15
12.83
1.94
4.83
4.84
2.24
4.78
2.15
13 12 11 10 9 8 7 6 5 4 3 2 1
f1 (ppm)

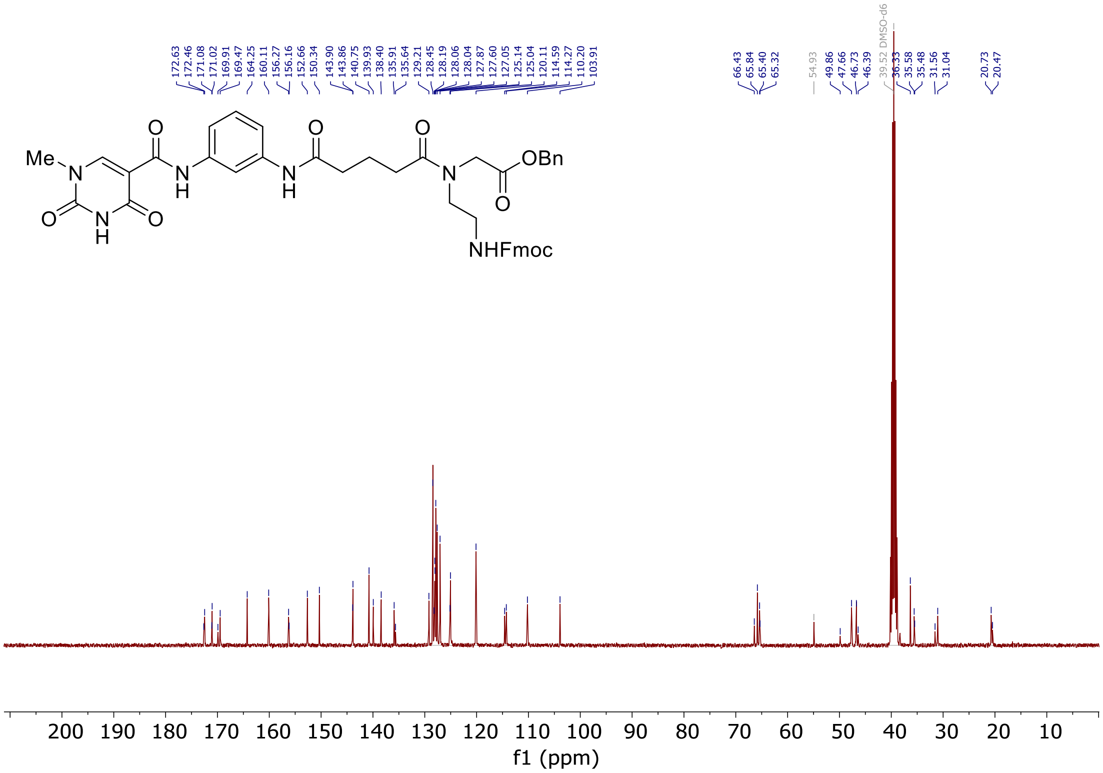
172.63
172.46
171.08
171.02
169.91
169.47
164.25
160.11
156.27
156.16
152.66
150.34
143.90
143.86
140.75
139.93
138.40
135.91
135.64
129.21
128.45
128.19
128.06
128.04
127.87
127.60
127.05
125.14
125.04
120.11
114.59
114.27
110.20
103.91
66.43
65.84
65.40
65.32
54.93
49.86
47.66
46.73
46.39
39.52 DMSO-d6
36.33
35.58
35.48
31.56
31.04
20.73
20.47
Me
NHFmoc
OBn
200 190 180 170 160 150 140 130 120 110 100 90 80 70 60 50 40 30 20 10
f1 (ppm)

Compound 15

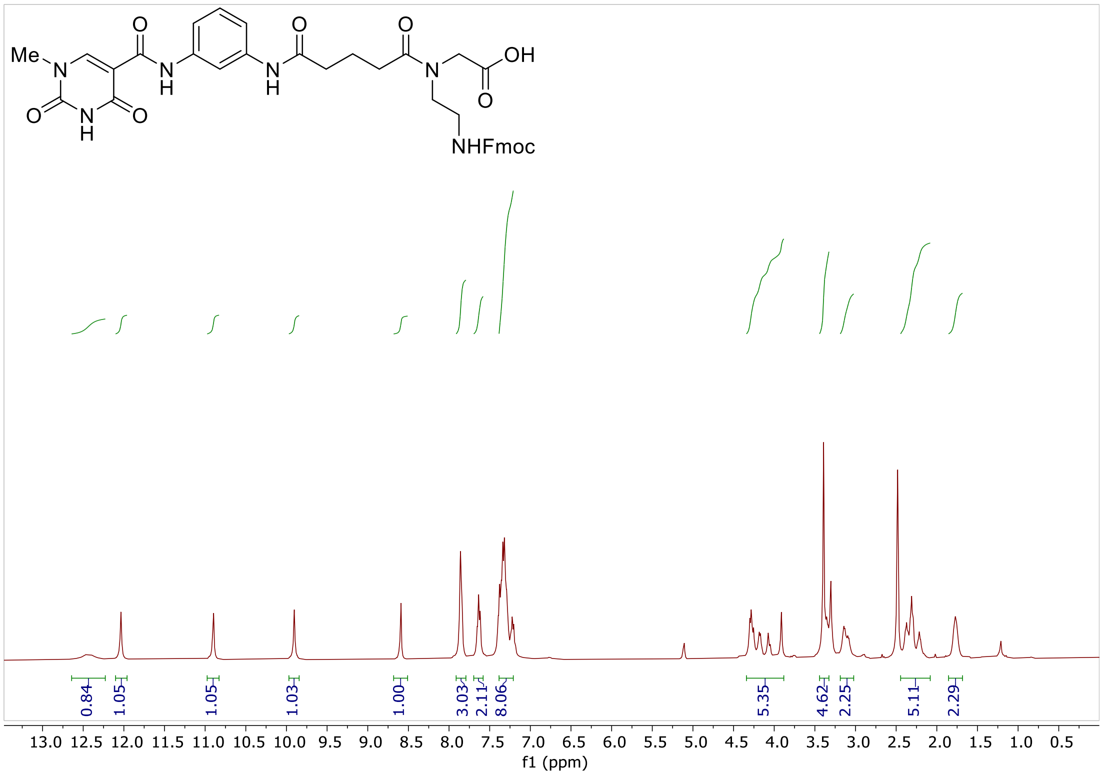

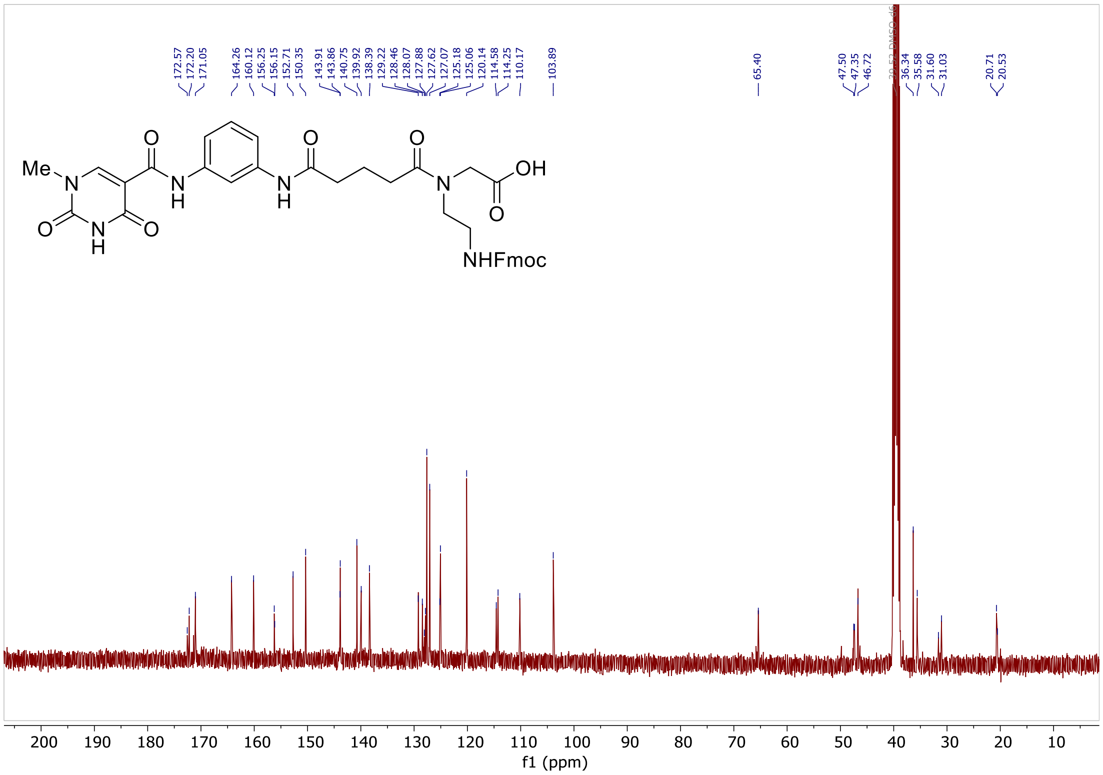

Compound 17

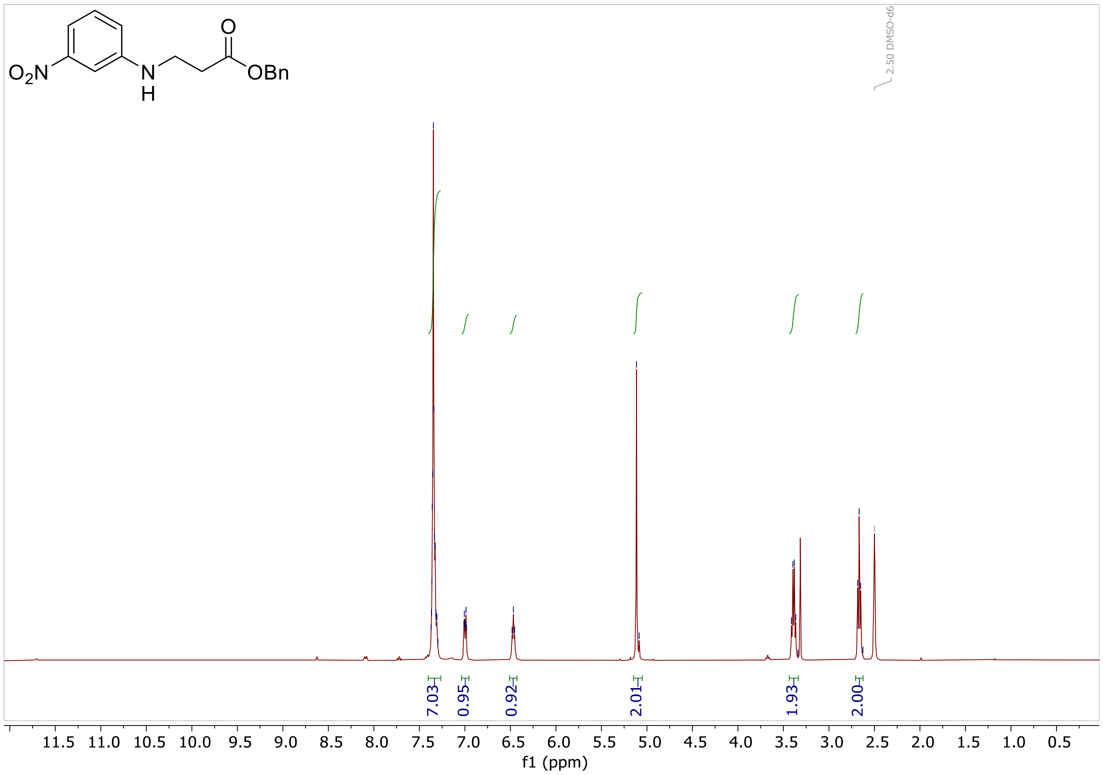

O_2N
N
H
O
OBn
2.50 DMSO-d6
7.03
0.95
0.92
2.01
1.93
2.00
11.5 11.0 10.5 10.0 9.5 9.0 8.5 8.0 7.5 7.0 6.5 6.0 5.5 5.0 4.5 4.0 3.5 3.0 2.5 2.0 1.5 1.0 0.5
f1 (ppm)

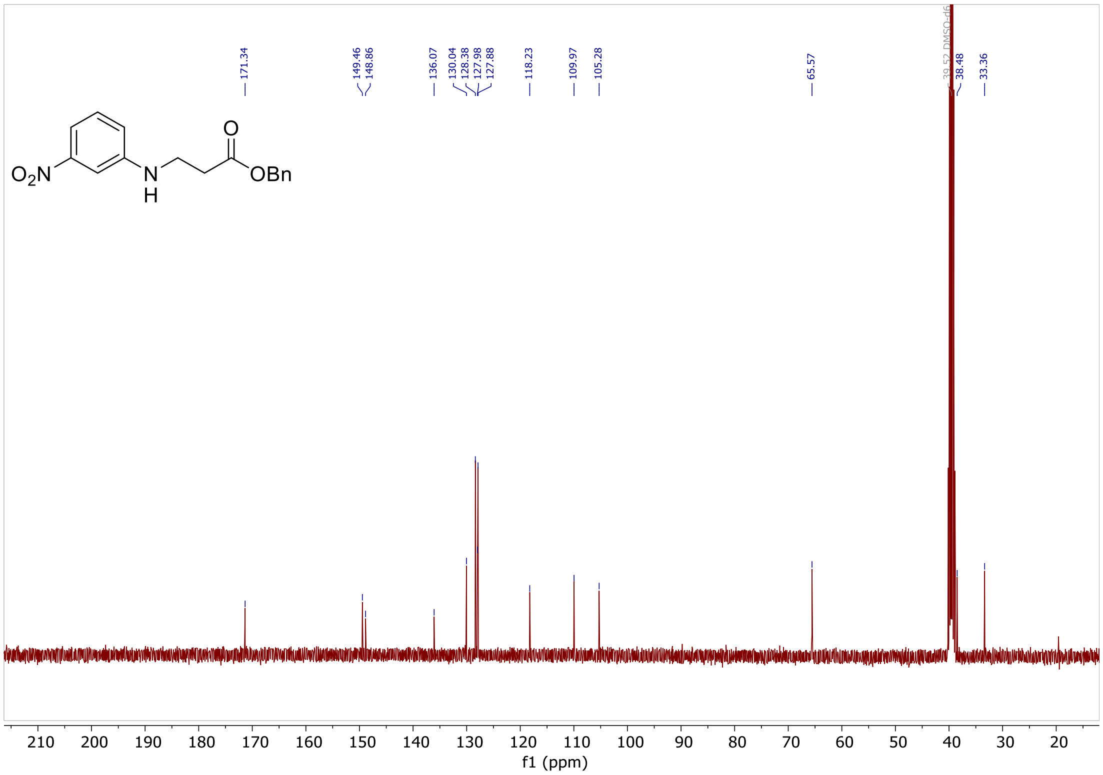

171.34
149.46
148.86
136.07
130.04
128.38
127.98
127.88
118.23
109.97
105.28
65.57
39.52 DMSO-d6
38.48
33.36
O_2N
N
H
O
OBn
210 200 190 180 170 160 150 140 130 120 110 100 90 80 70 60 50 40 30 20
f1 (ppm)

Compound 18

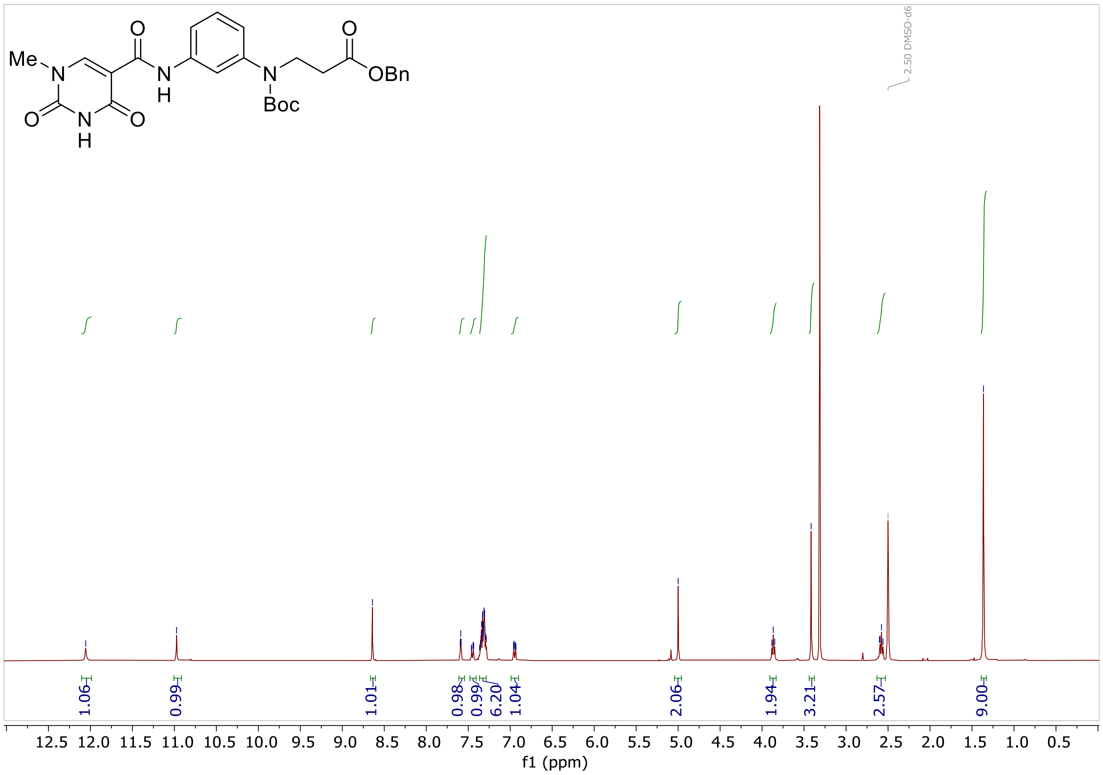

Me
N
O
N
H
O
N
H
N
Boc
O
OBn
2.50 DMSO-d6
1.06
0.99
1.01
0.98
0.99
6.20
1.04
2.06
1.94
3.21
2.57
9.00
12.5 12.0 11.5 11.0 10.5 10.0 9.5 9.0 8.5 8.0 7.5 7.0 6.5 6.0 5.5 5.0 4.5 4.0 3.5 3.0 2.5 2.0 1.5 1.0 0.5
f1 (ppm)

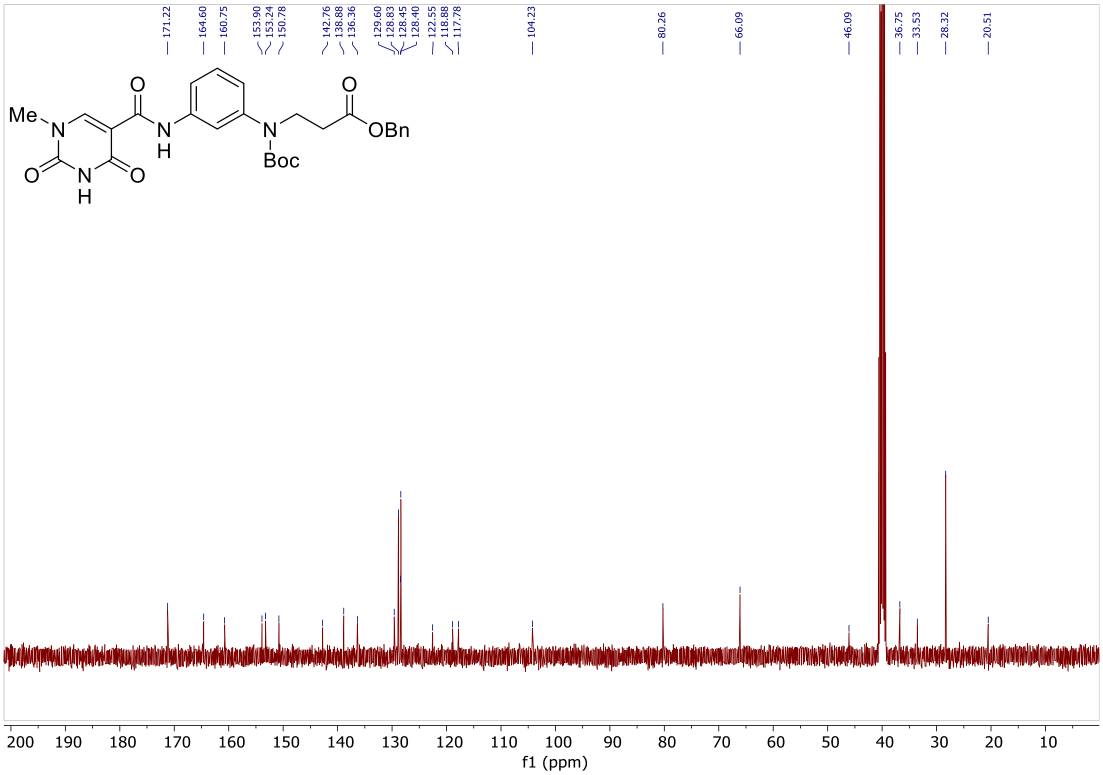

171.22
164.60
160.75
153.90
153.24
150.78
142.76
138.88
136.36
129.60
128.83
128.45
128.40
122.55
118.88
117.78
104.23
80.26
66.09
46.09
36.75
33.53
28.32
20.51
Me
N
O
N
H
O
N
H
N
Boc
O
OBn
200 190 180 170 160 150 140 130 120 110 100 90 80 70 60 50 40 30 20 10
f1 (ppm)

Compound 19

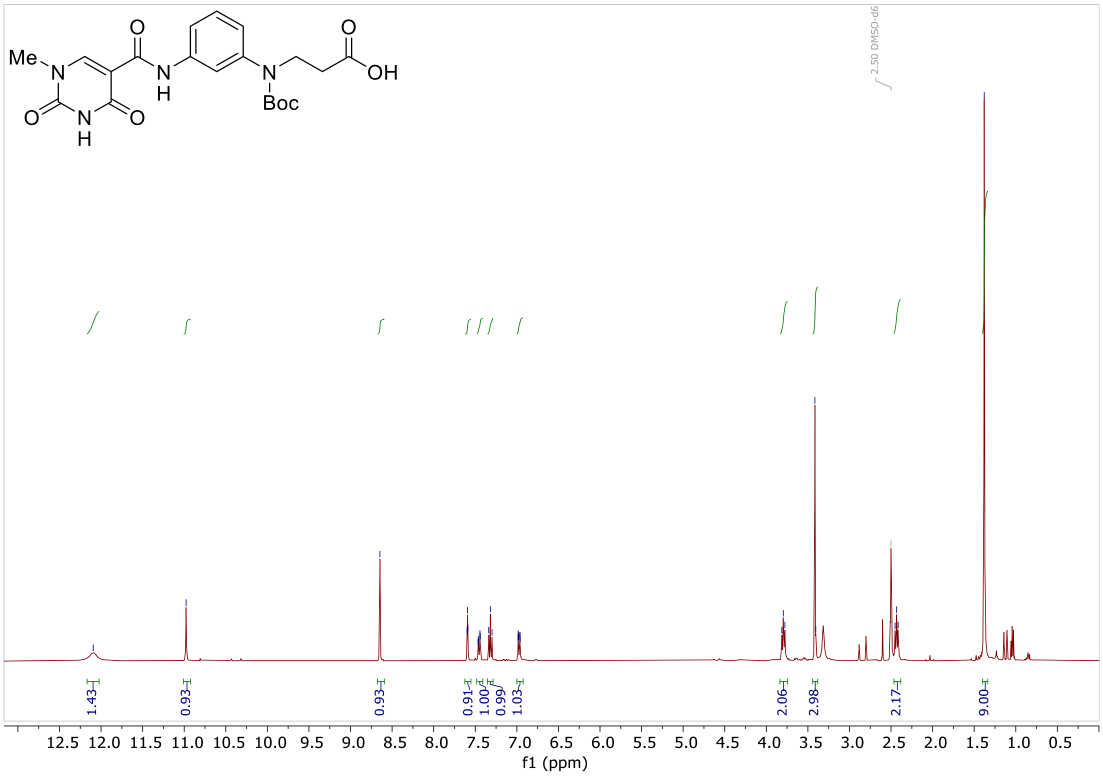

Me
N
O
N
H
O
O
N
H
N
Boc
O
OH
2.50 DMSO-d6
1.43
0.93
0.93
0.91
1.00
0.99
1.03
2.06
2.98
2.17
9.00
12.5 12.0 11.5 11.0 10.5 10.0 9.5 9.0 8.5 8.0 7.5 7.0 6.5 6.0 5.5 5.0 4.5 4.0 3.5 3.0 2.5 2.0 1.5 1.0 0.5
f1 (ppm)

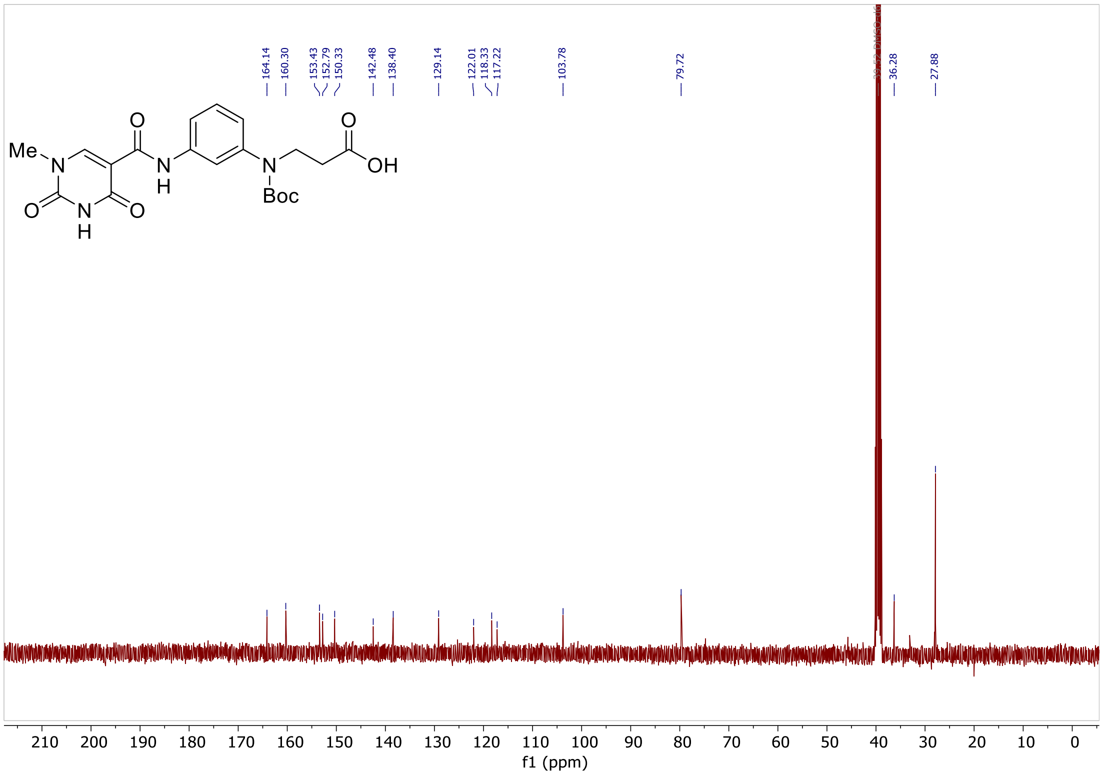

164.14
160.30
153.43
152.79
150.33
142.48
138.40
129.14
122.01
118.33
117.22
103.78
79.72
36.28
27.88
Me
N
O
N
H
O
O
N
H
N
Boc
O
OH
210 200 190 180 170 160 150 140 130 120 110 100 90 80 70 60 50 40 30 20 10 0
f1 (ppm)

Compound 20

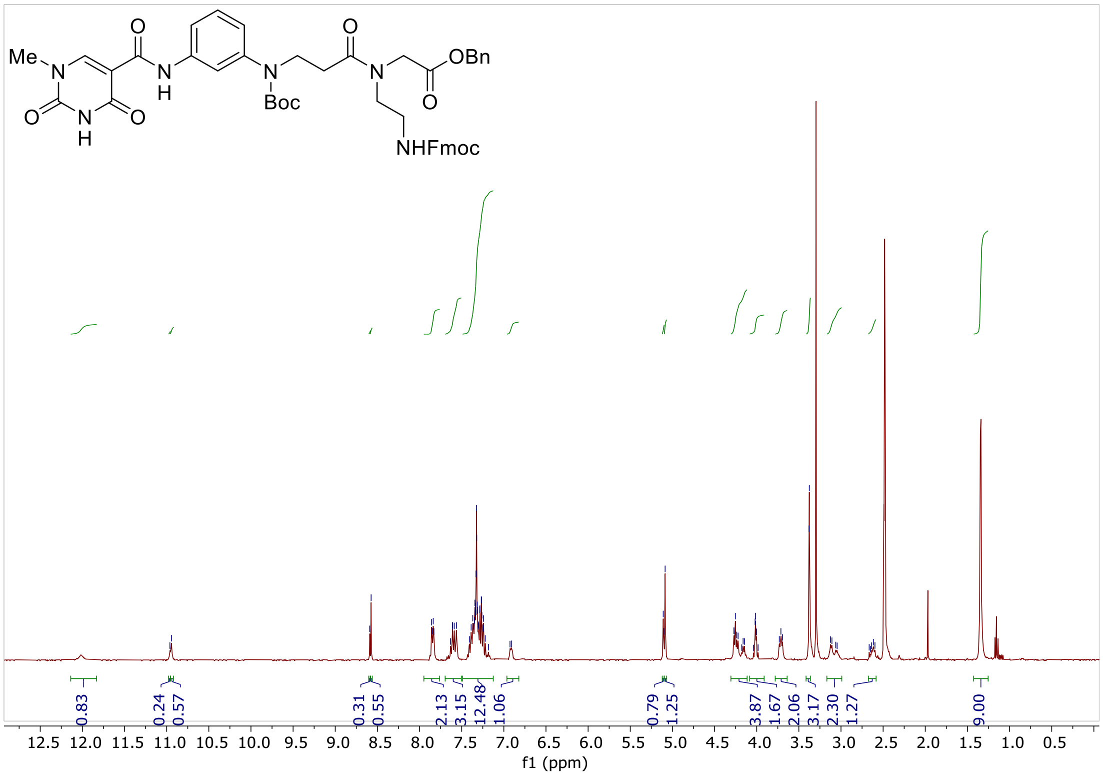

Me
N
N
H
O
O
O
N
H
N
Boc
O
N
OBn
O
NHFmoc
0.83
0.24
0.57
0.31
0.55
2.13
3.15
12.48
1.06
0.79
1.25
3.87
1.67
2.06
3.17
2.30
1.27
9.00
12.5 12.0 11.5 11.0 10.5 10.0 9.5 9.0 8.5 8.0 7.5 7.0 6.5 6.0 5.5 5.0 4.5 4.0 3.5 3.0 2.5 2.0 1.5 1.0 0.5
f1 (ppm)

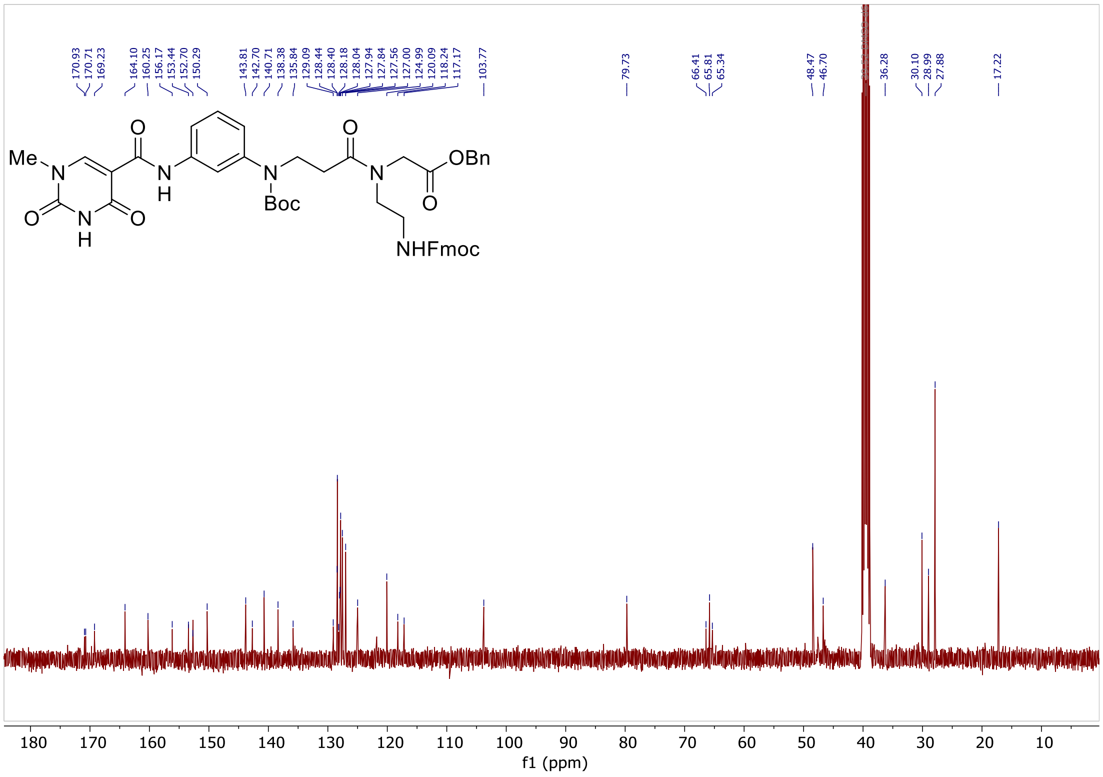

170.93
170.71
169.23
164.10
160.25
156.17
153.44
152.70
150.29
143.81
142.70
140.71
138.38
135.84
129.09
128.44
128.40
128.18
128.04
127.94
127.84
127.56
127.00
124.99
120.09
118.24
117.17
103.77
79.73
66.41
65.81
65.34
48.47
46.70
36.28
30.10
28.99
27.88
17.22
Me
N
N
H
O
O
O
N
H
N
Boc
O
N
OBn
O
NHFmoc
180 170 160 150 140 130 120 110 100 90 80 70 60 50 40 30 20 10
f1 (ppm)

Compound 21

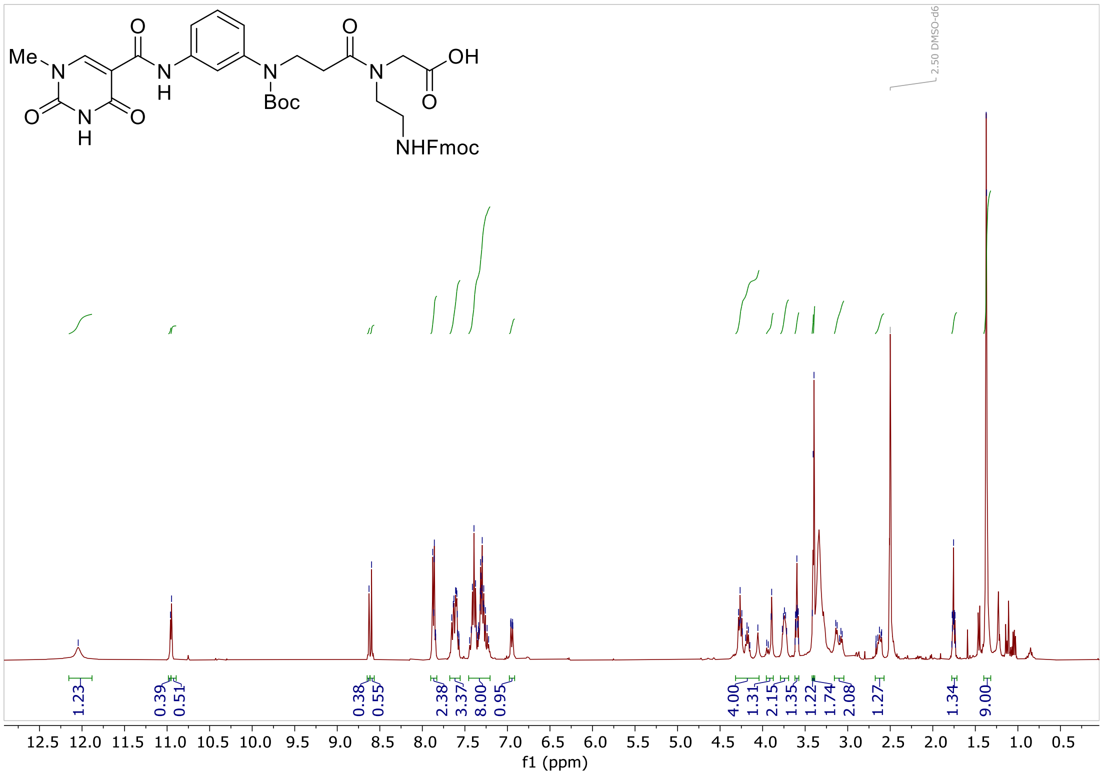

Me
N
H
O
Boc
OH
NHFmoc
2.50 DMSO-d6
1.23
0.39
0.51
0.38
0.55
2.38
3.37
8.00
0.95
4.00
1.31
2.15
1.35
1.22
1.74
2.08
1.27
1.34
9.00
12.5 12.0 11.5 11.0 10.5 10.0 9.5 9.0 8.5 8.0 7.5 7.0 6.5 6.0 5.5 5.0 4.5 4.0 3.5 3.0 2.5 2.0 1.5 1.0 0.5
f1 (ppm)

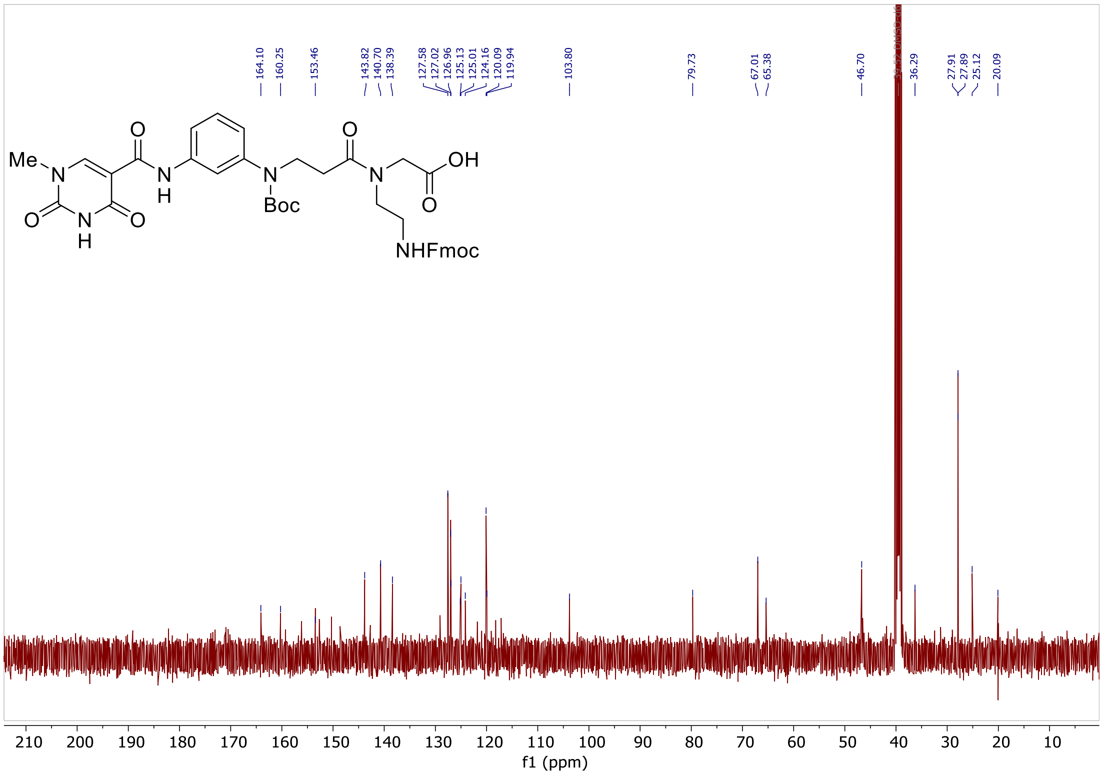

164.10
160.25
153.46
143.82
140.70
138.39
127.58
127.02
126.96
125.13
125.01
124.16
120.09
119.94
103.80
79.73
67.01
65.38
46.70
36.29
27.91
27.89
25.12
20.09
Me
N
H
O
Boc
OH
NHFmoc
210 200 190 180 170 160 150 140 130 120 110 100 90 80 70 60 50 40 30 20 10
f1 (ppm)

Compound SI-4

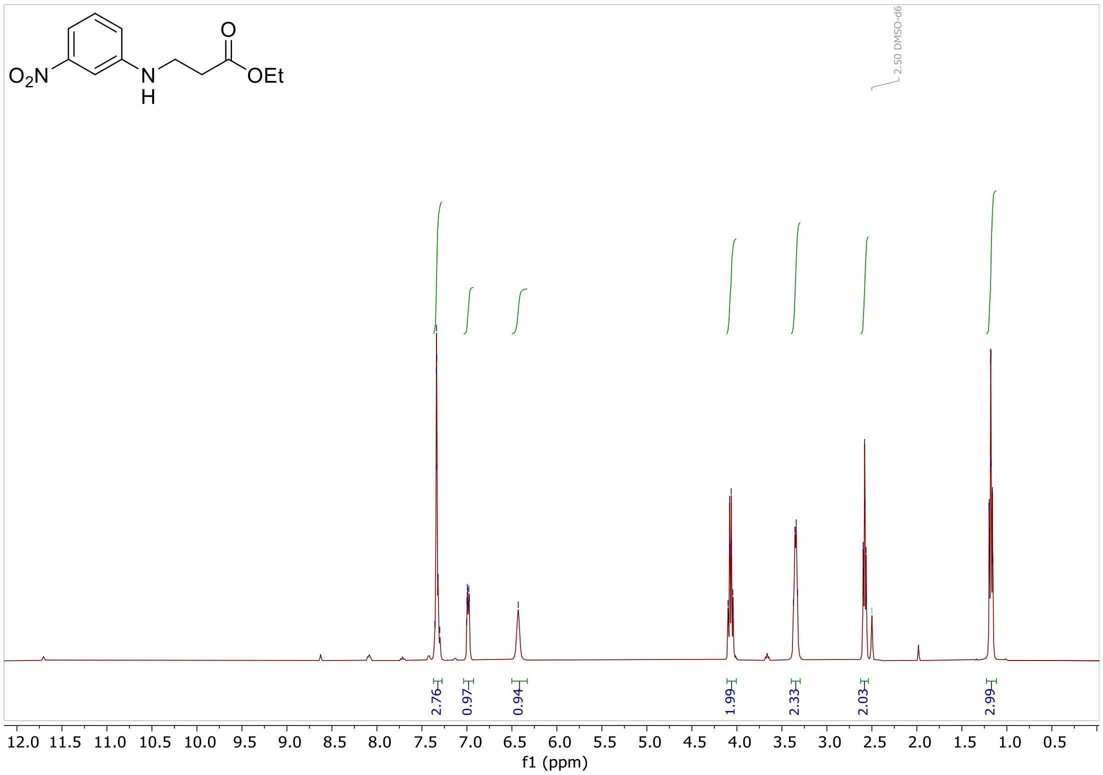

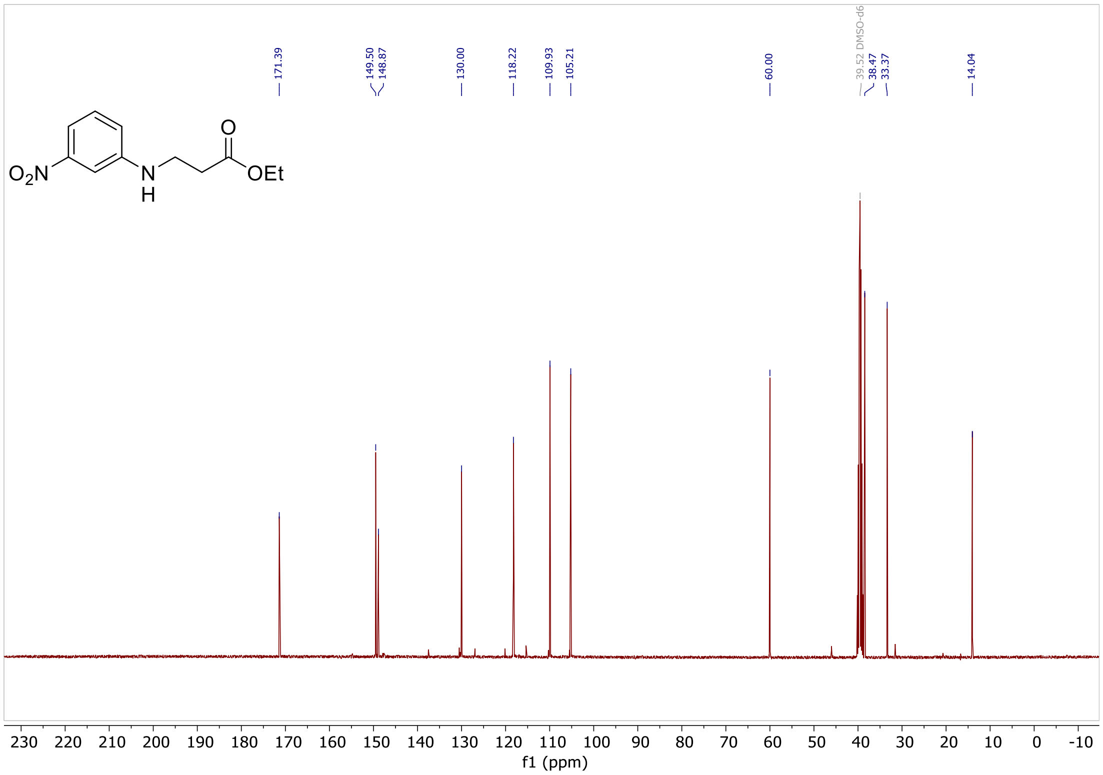

Compound SI-5

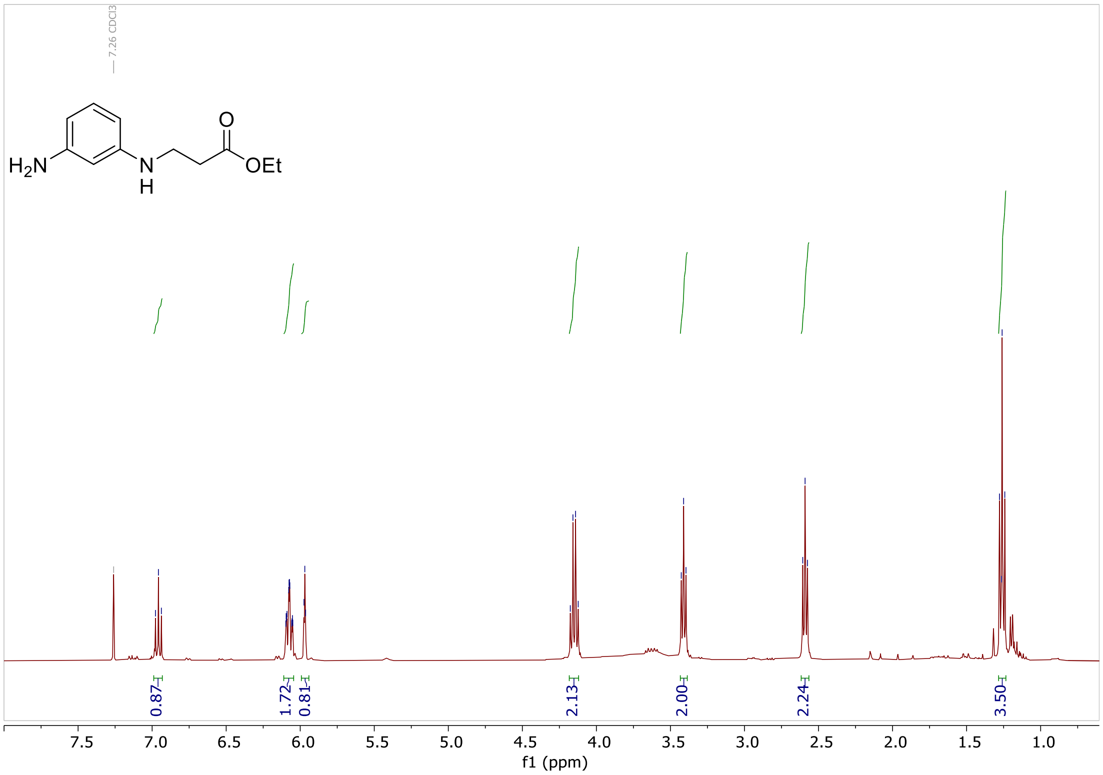

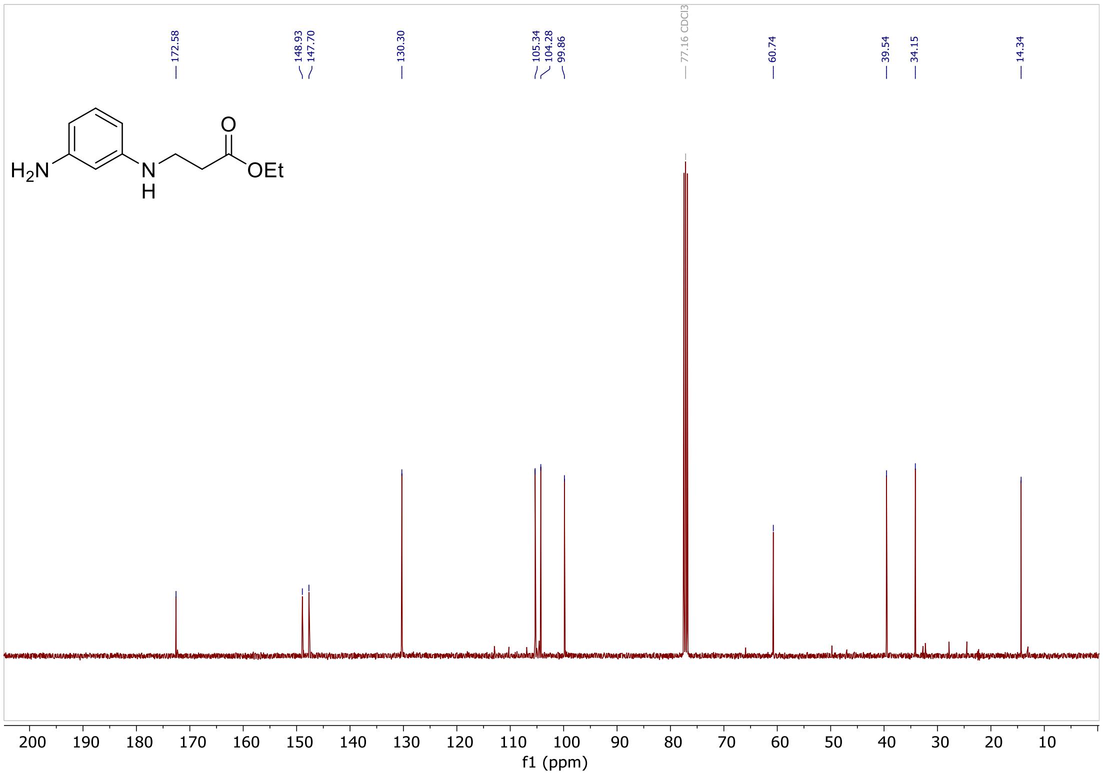

Compound SI-6

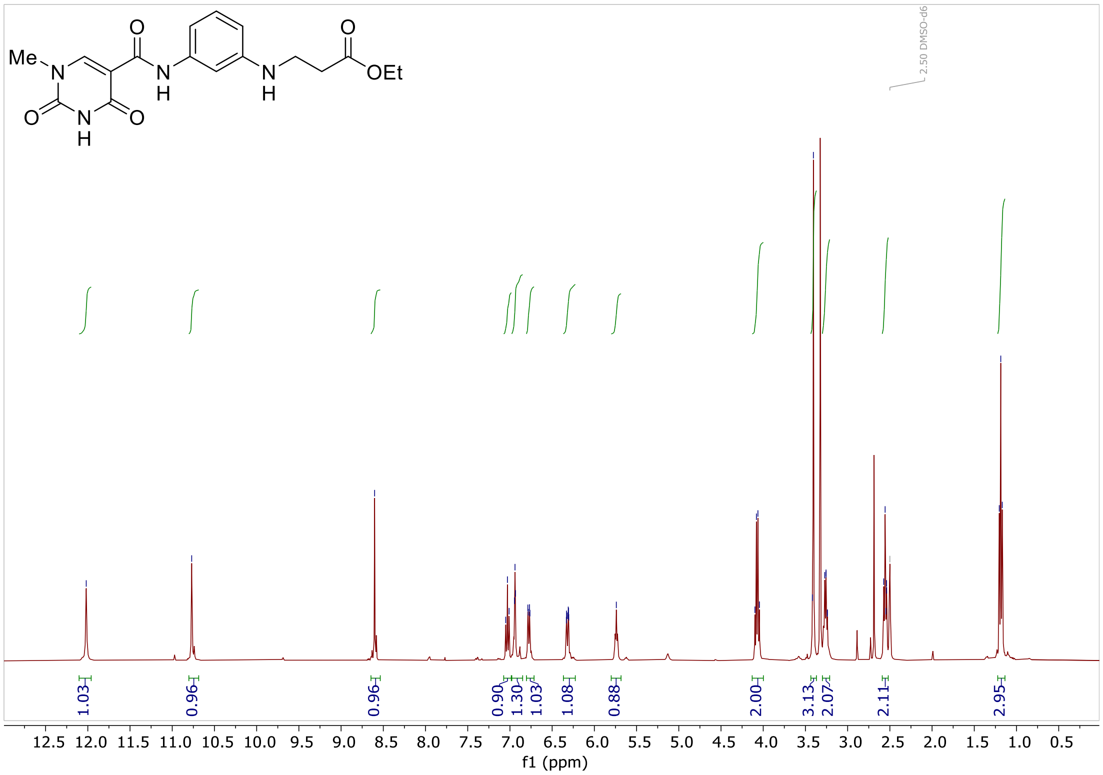

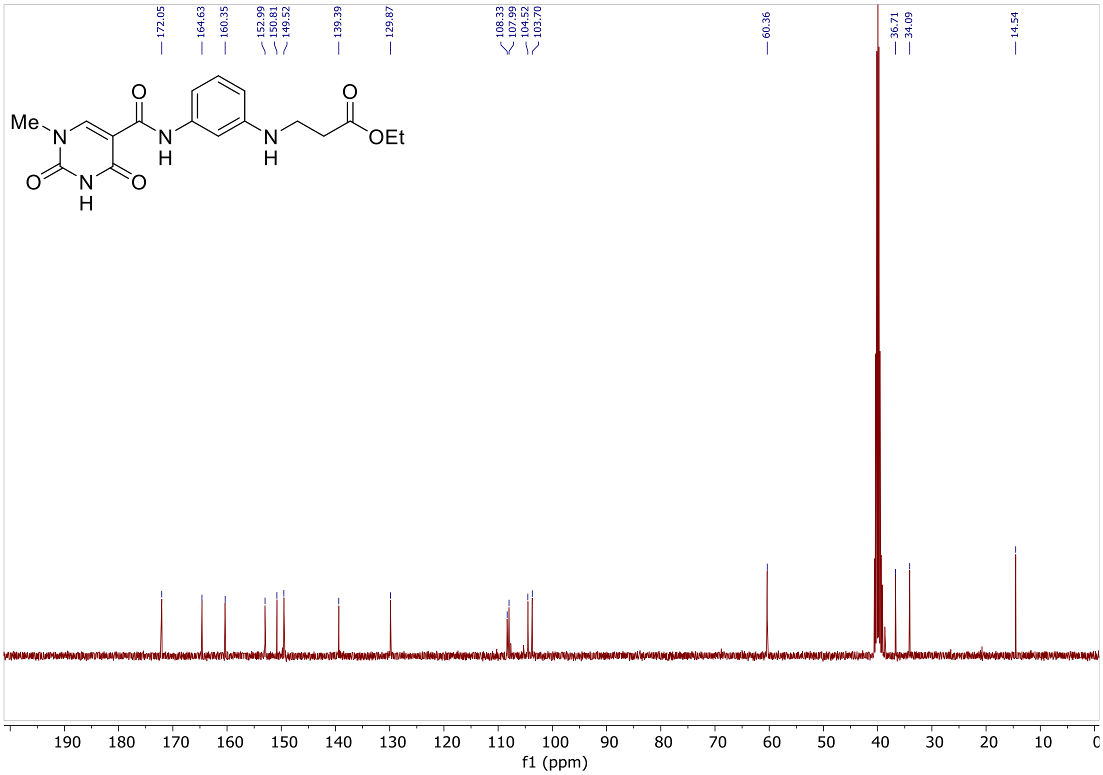

Procedures for PNA synthesis, quantification, and purification.

<u>*PNA synthesis*</u>

PNA-T-monomer was purchased from Link Technologies and **M** monomer was synthesized using a reported synthetic route [5]. PNA oligomers were synthesized on 2 µmol scale using TentaGel XV RAM (Rapp Polymere) on an automated Expedite 8909 Nucleic Acid Synthesizer using standard protocols [6]. The PNA strands were cleaved from the resin using 0.6 mL of a cleavage cocktail (95% TFA, 2.5% triisopropylsilane, 2.5% H_2O) using a two-syringe push-pull method over four hours, followed by a second wash with another 0.2 mL of cleaving cocktail. The crude PNA (200 µL in four separate Eppendorf tubes) was precipitated with cold diethyl ether, followed by centrifugation. Ether was poured off and the remaining pellet of crude PNA was dissolved in distilled water and purified by reverse phase HPLC on a Shimadzu LC-40 instrument using a Supelco Discovery Wide Pore C18 Column (4.6 × 150 mm) at 55 °C using 0.1% formic acid in water and acetonitrile with a linear gradient of 3–9% acetonitrile for all PNAs. The identity and purity of PNAs were determined by LCMS analysis using a Shimadzu 2020 single quadrupole instrument with a Cosmosil C18-MS-II column (50 mm × 2 mm × 2.5 µm) at 55 °C using 0.1% formic acid in water and acetonitrile with a linear gradient of 0–15% acetonitrile for PNA oligomers, over 30 minutes with a flow rate of 0.400 mL/min. The deconvoluted masses calculated and observed for each PNA strand in the **Db** series is reported in Table SI-1 and the LC/MS spectra for modified PNA oligomers can be seen in Figures S1 to S3.

Table S1: Mass characterization of PNAs

PNA	Mass Calc.	M/Z calc	M/Z found
PNA 1 (**Db1**)	2543	424.7, 509.5, 636.6, 1272.3	424.9, 509.7, 636.9, 1272.9
PNA 2 (**Db2**)	2571	515.1, 643.6, 857.9, 1286.3	515.3, 643.9, 858.2, 1286.8
PNA 3 (**Db3**)	2529	506.7, 633.1, 843.9, 1265.3	506.9, 633.4, 844.2, 1266.3

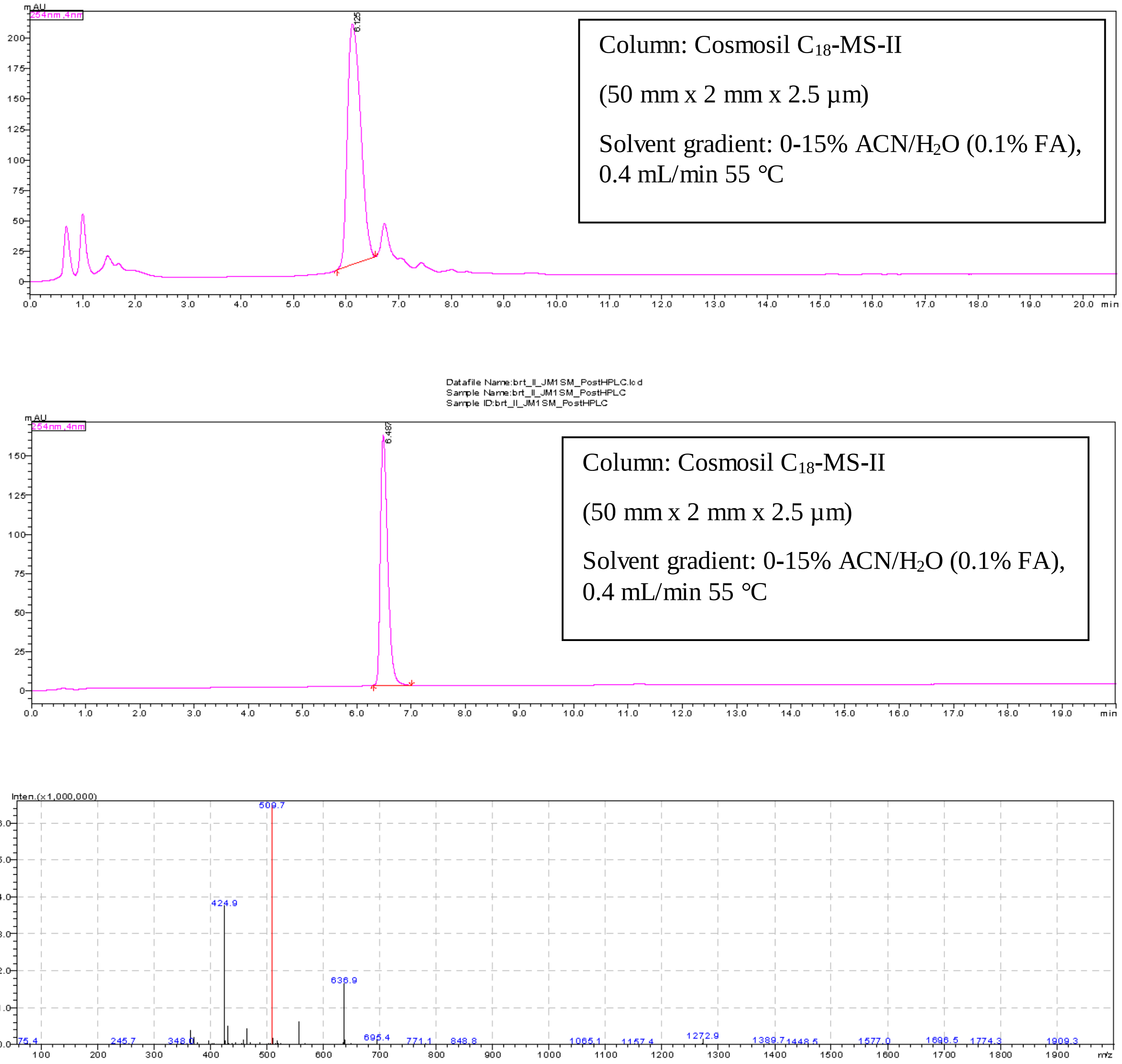

Figure S1: Crude (top) and pure (bottom) LC/MS of PNA 1

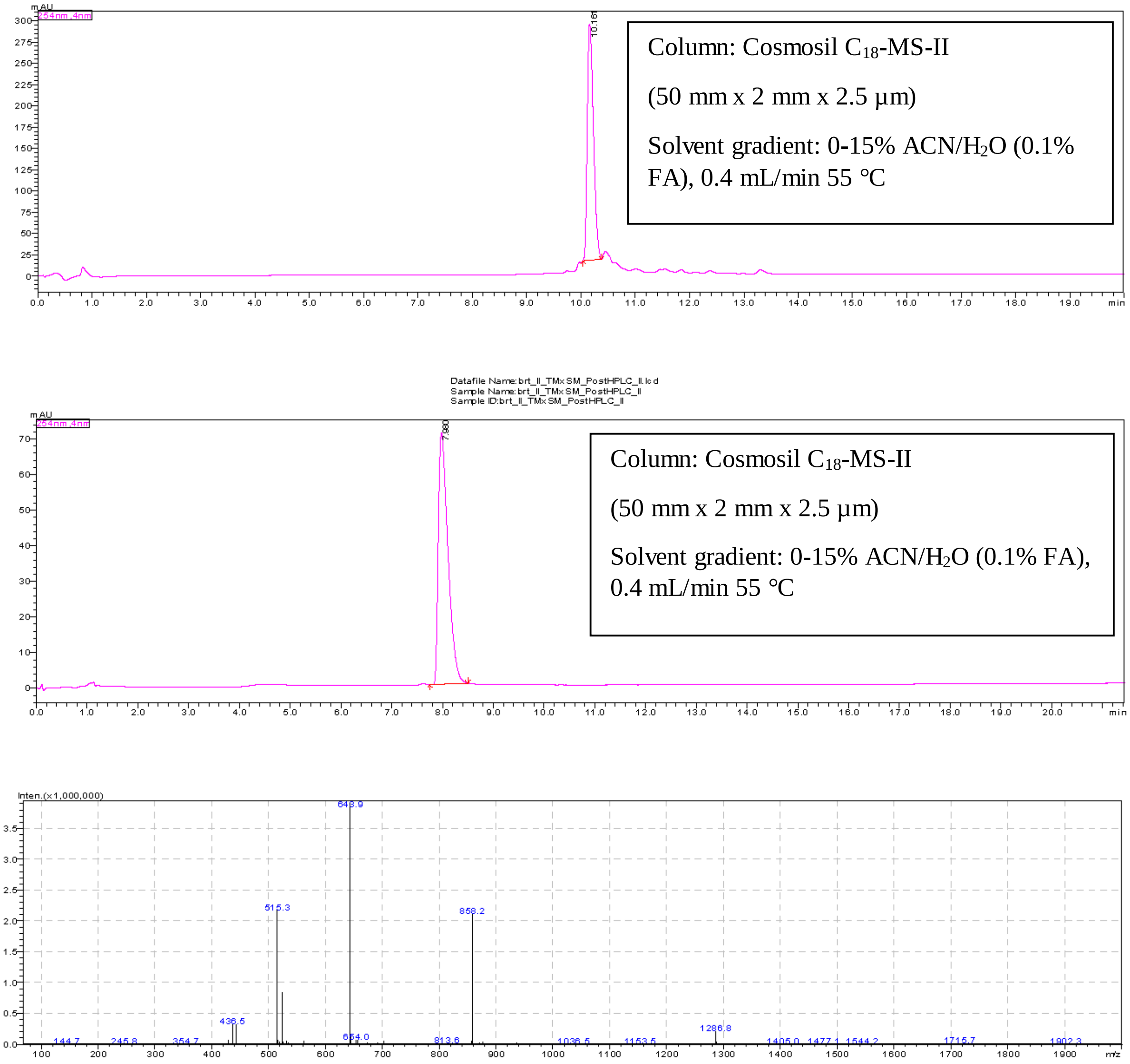

Figure S2: Crude (top) and pure (bottom) LC/MS of PNA 2

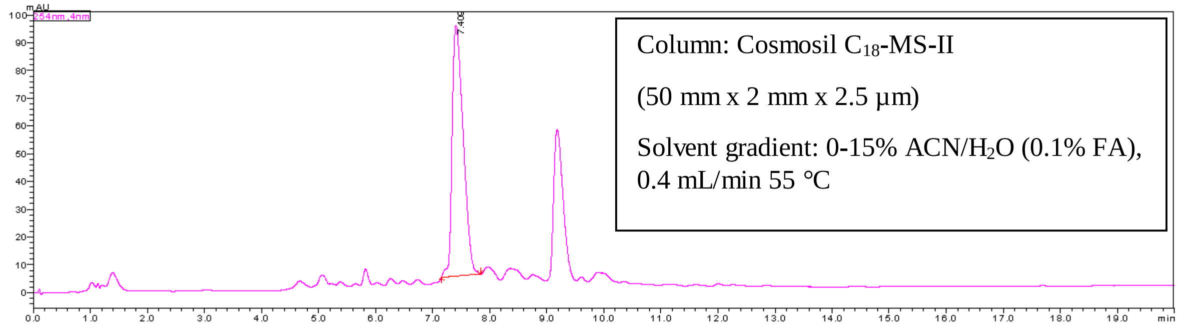

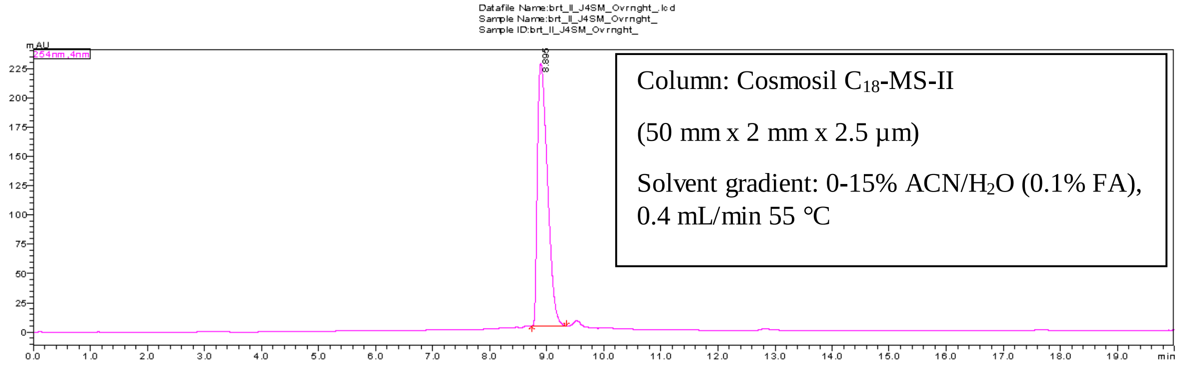

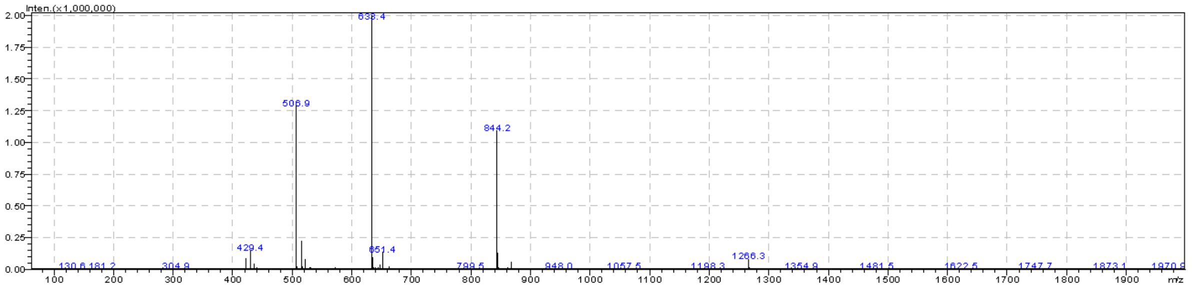

Figure S3: Crude (top) and pure (bottom) LC/MS of PNA 3

Extinction coefficient determinations

Molar extinction coefficients were calculated using standard protocol [7] for compounds **SI-7**, **10**, and **SI-6**. The Boc protecting group of **SI-1** was cleaved with 10% TFA in CH_2Cl_2 to obtain the TFA Salt (**SI-7**). Beer-Lamber's Law (A= ε c l) was used by measuring the absorbance at 260 nm of three different known concentrations of the nucleobase derivative. The absorbance readings were measured in a 1.0 mL cuvette of pathlength 1.0 cm and tabulated as shown in the table below (Table S2).

Table S2: Calculation of molar extinction coefficient for ***Db1*** monomer

Nucleobase derivative	M.Wt. (g/mol)	Wt. (mg)	Con. (mM)	Dilution Factor	Abs. at 260 nm	ε_{260} (M^{-1} cm^{-1} L)
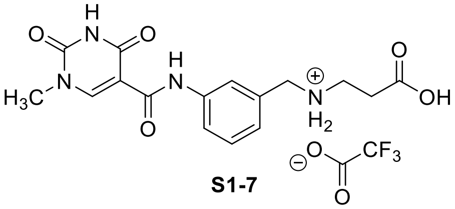 S1-7	460.4	3	0.26	25	1.5	5700
		2.3	0.20	25	1.1	5300
		1.9	0.17	25	0.96	5800
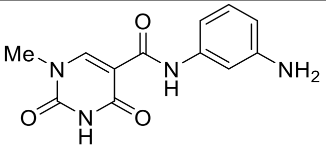 10	260.3	1.8	0.28	25	1.2	4300
		2.2	0.34	25	1.5	4300
		2.6	0.40	25	1.5	3700
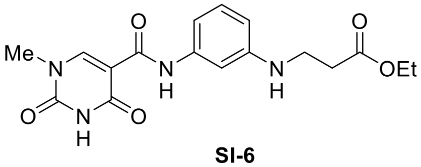 SI-6	360.37	2.0	0.22	25	2.4	11000
		1.5	0.17	25	1.8	11000
		1.9	0.21	25	2.1	10000

The molar extinction coefficient of the PNA sequence was calculated by summing up the individual extinction coefficients of the monomers using the above numbers and 8560 $M^{-1}cm^{-1}L$ for T and 806 $M^{-1}cm^{-1}L$ for M. The average molar extinction coefficients are the following; **SI-**

7, ε_{260} = 5600 ± 270 $M^{-1}cm^{-1}L$. **10**, ε_{260} = 4,100 ± 340 $M^{-1}cm^{-1}L$, **SI-6**, ε_{260} =11000 ± 430 $M^{-1}cm^{-1}L$.

Biophysical assays

Preparation of RNA hairpins

The commercial 2'-O-ACE protected hairpin RNAs were purchased from Dharmacon, Inc. The 2'-O-ACE protection of hairpin RNAs was removed using the recommended protocol from Dharmacon, Inc. The crude RNAs were purified on reverse phase HPLC using 50 mM TEAA buffer, pH ~7.4. The purified samples were lyophilized and re-dissolved in deionized water and again lyophilized. This process repeated a couple more times to get rid of the TEAA volatiles yielding hairpin RNAs as a white foam.

UV thermal melting studies

UV melting studies were performed on a Shimadzu UV-2600 spectrophotometer equipped with a TMSPC-8 temperature controller. The UV thermal melting temperature (T_m, °C) is an average of six 18 µM replicates performed in phosphate buffer (2 mM $MgCl_2$, 90 mM KCl, 10 mM NaCl, and 50 mM potassium phosphate at pH 7.4). The absorbance of the solution as the triplex denatured was measured at 300 nm, with the temperature increasing at a rate of 1 °C per minute. The absorbance was plotted against the temperature, producing a sigmoidal curve with the mid-point corresponding to the melting temperature (T_m) that was obtained using Shimadzu LabSolutions T_m Analysis software version 1.31.

Table S3: UV-Metling data (°C) for PNA 1 (Db1)

Hairpin	HRP1 (U-A)	HRP2 (G-C)	HRP3 (A-U)	HRP4 (C-G)
Melting Temp. (°C)	41.8<br>42.2<br>41.8<br>42.0<br>41.2<br>41.5	35.9<br>35.0<br>36.2<br>35.9<br>36.5<br>35.8	35.7<br>36.3<br>36.2<br>36.6<br>36.1<br>35.8	38.2<br>38.6<br>38.2<br>37.7<br>38.4<br>37.2
Average<br>St. dev.	41.8<br>0.4	35.9<br>0.5	36.1<br>0.3	38.0<br>0.5

Table S4: UV-Metling data (°C) for PNA 2 (Db2)

Hairpin	HRP1 (U-A)	HRP2 (G-C)	HRP3 (A-U)	HRP4 (C-G)
Melting Temp. (°C)	36.3<br>36.0<br>36.2<br>36.1<br>36.5<br>36.2	27.3<br>26.7<br>28.3<br>27.1<br>27.8<br>27.7	33.7<br>34.1<br>33.4<br>32.8<br>32.9<br>32.8	41.7<br>41.7<br>41.2<br>41.4<br>41.3<br>41.2
Average<br>St. dev.	36.2<br>0.2	27.5<br>0.6	33.3<br>0.5	41.4<br>0.2

Table S5: UV-Metling data (°C) for PNA 3 (Db3)

Hairpin	HRP1 (U-A)	HRP2 (G-C)	HRP3 (A-U)	HRP4 (C-G)
Melting Temp. (°C)	41.3<br>41.6<br>41.3<br>41.5<br>40.9<br>41.0	27.3<br>27.1<br>27.8<br>27.3<br>27.9<br>27.7	34.3<br>34.2<br>34.5<br>34.5<br>34.6<br>34.2	38.9<br>38.6<br>38.2<br>38.4<br>37.9<br>38.0
Average<br>St. dev.	41.3<br>0.3	27.5<br>0.3	34.4<br>0.2	38.3<br>0.4

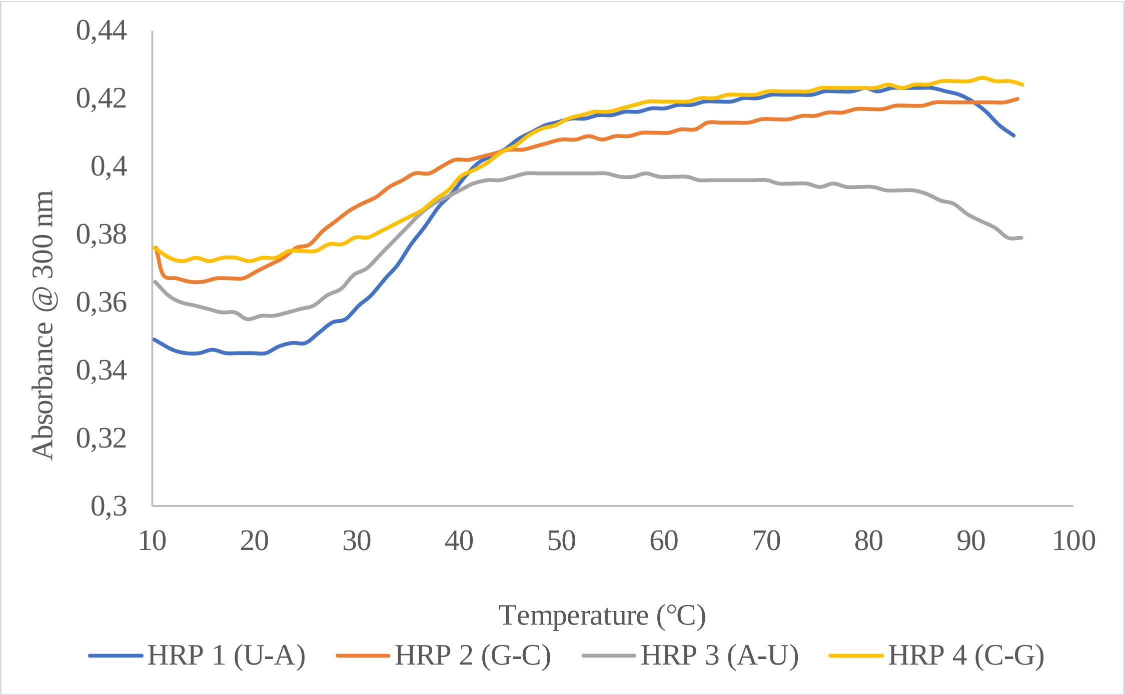

Figure S4: Representative UV-melting curves for PNA 1 (Db1)

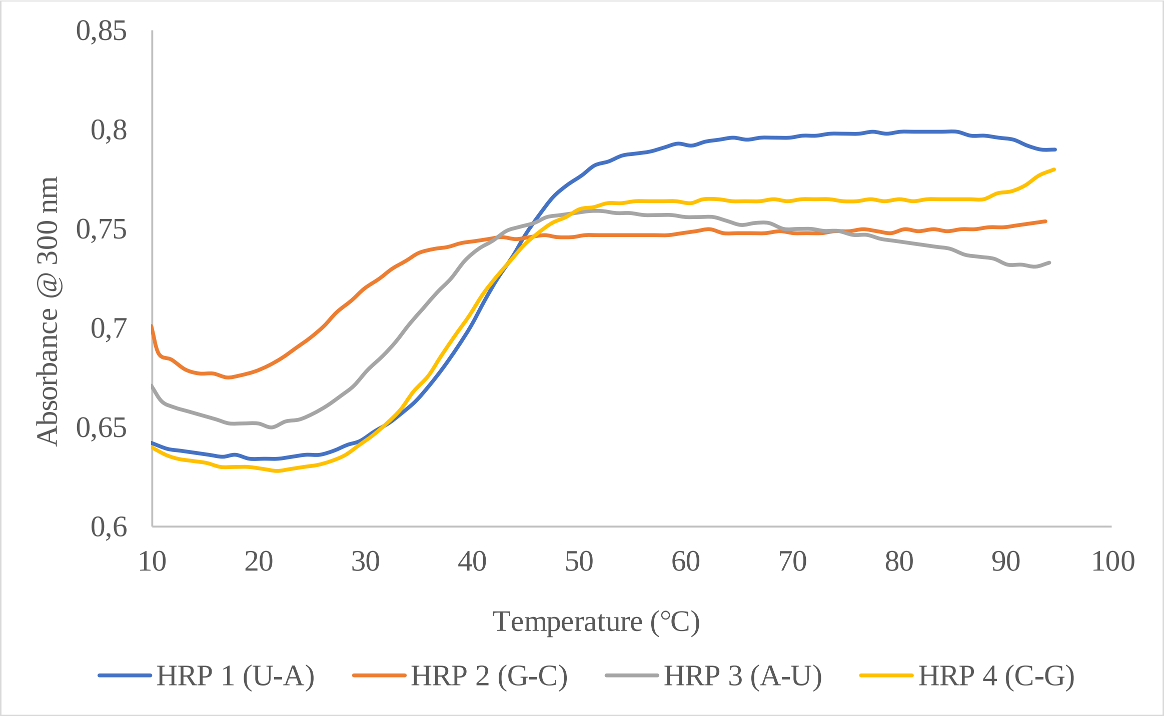

Figure S5: Representative UV-melting curves for PNA 2 (Db2)

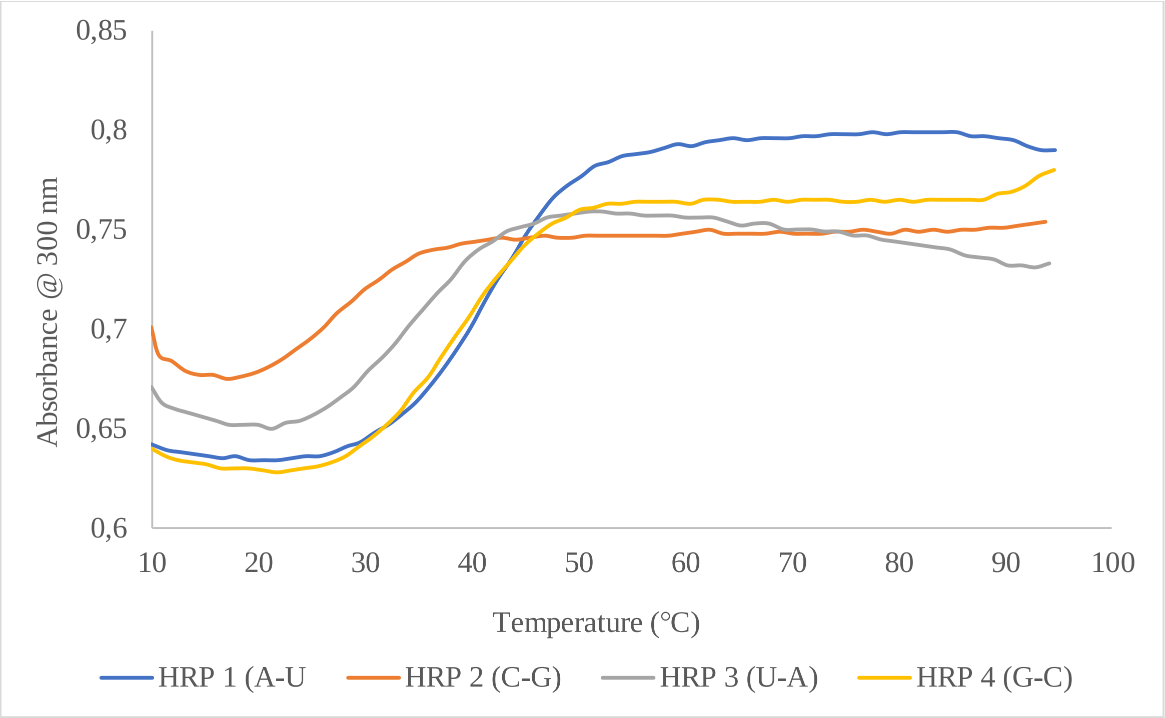

Figure S6: Representative UV-melting curves for PNA 3 (Db3)

Isothermal titration calorimetry experiments

Isothermal titration calorimetry experiments were performed on a MicroCal ITC200 instrument at 25 °C in 50 mM potassium phosphate buffer (pH 7.4) containing 2 mM $MgCl_2$, 90 mM KCl, and 10 mM NaCl. Solutions were prepared as follows. Stock solutions of PNA (20 µL of 240 µM) and the respective RNA hairpin (12.5 µL of 240 µM) were aliquoted and lyophilized. 64 µL of buffer was added to the PNA pellet, and 300 µL of buffer was added to the RNA hairpin pellet. Solutions were then vortexed and centrifuged to allow for proper mixing. The solution of the RNA hairpin was then heated to 95 °C for 5 minutes and then cooled to 25 °C to allow for correct folding. The standard concentration of PNA and RNA was 10 µM and 75 µM, respectively. Aliquots of PNA solution (2.45 µL) were sequentially injected from a 40 µL rotating syringe (750 rpm) into 200 µL of RNA solution. Results were analyzed using MicroCal PEAQ-ITC software.

Table S6: PNA 1 binding affinity and thermodynamic data obtained by ITC

Hairpin	N (sites)	Kd (M)	Ka (M-1)	ΔH (kcal/mol)	ΔG (kcal/mol)	-TΔS(kcal/mol)
HRP1 01	0.85	2.16E-07	4.63E+06	-80	-9.1	71
HRP1 02	0.81	2.21E-07	4.52E+06	-64	-9.08	55
HRP1 03	0.87	2.15E-07	4.65E+06	-80	-9.11	71
Average	0.84	2.17E-07	4.60E+06	-75	-9.10	66
St. Dev.	0.03	3E-09	7E+04	9	0.02	9

Table S7: PNA 2 binding affinity and thermodynamic data obtained by ITC

Hairpin	N (sites)	Kd (M)	Ka (M-1)	ΔH (kcal/mol)	ΔG (kcal/mol)	-TΔS(kcal/mol)
HRP1 01	0.78	1.05E-06	9.52E+05	-40	-8.17	32
HRP1 02	1.1	1.03E-06	9.71E+05	-37	-8.18	29
HRP1 03	0.84	9.89E-07	1.01E+06	-28	-8.2	20
Average	0.9	1.02E-06	9.78E+05	-35	-8.18	27
St. Dev.	0.2	3E-08	3.00E+04	6	0.02	6

Table S8: PNA 3 binding affinity and thermodynamic data obtained by ITC

Hairpin	N (sites)	Kd (M)	Ka (M-1)	ΔH (kcal/mol)	ΔG (kcal/mol)	-TΔS(kcal/mol)
HRP1 01	0.86	3.41E-07	2.93E+06	-46	-8.82	38
HRP1 02	0.81	2.97E-07	3.37E+06	-68	-8.91	59
HRP1 03	0.89	3.07E-07	3.26E+06	-40	-8.9	31
Average	0.85	3.15E-07	3.19E+06	-51	-8.88	43
St. Dev.	0.04	2E-08	2E+05	15	0.05	15

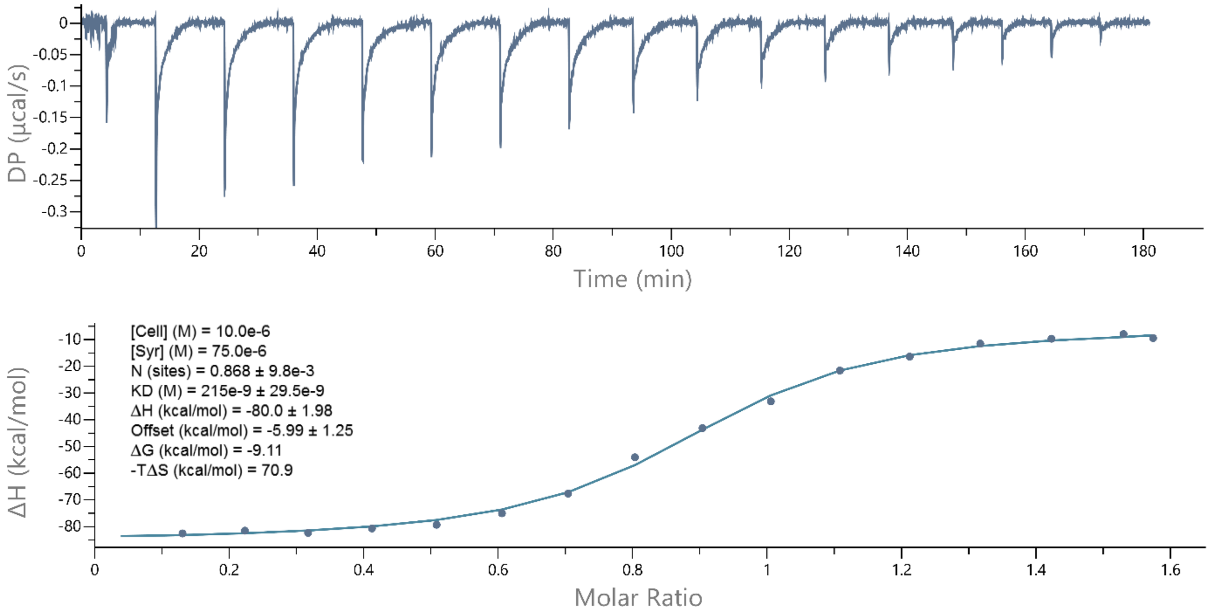

Figure S7: Representative ITC trace of PNA 1 (Db1)

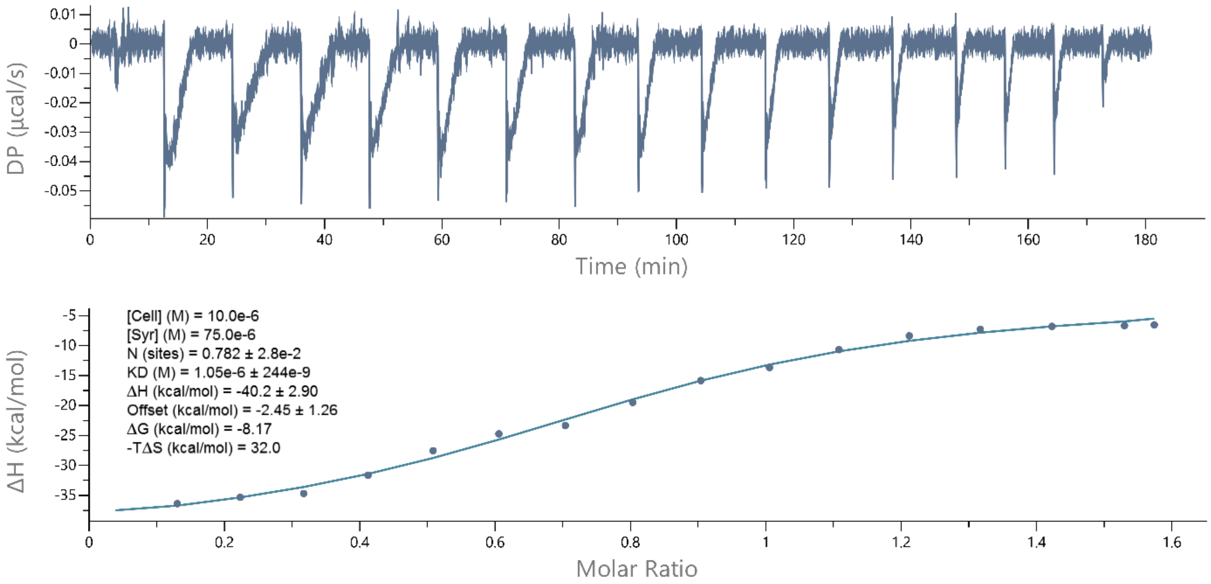

Figure S8: Representative ITC trace of PNA 2 (Db2)

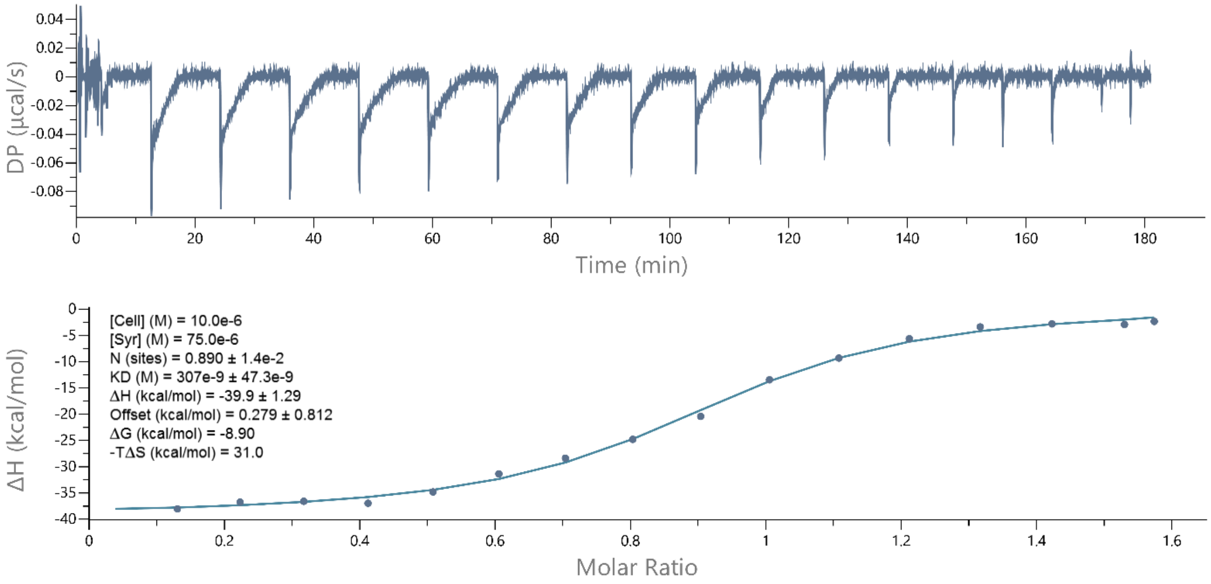

Figure S9: Representative ITC trace of PNA 3 (Db3)

Computational Details

The MD simulations were performed using Maestro Version 13.4.134, Release 2022-4 (Schrödinger, LLC). The initial PNA-dsRNA triple helix structure was derived from a previously validated model [8]. RNA sequences were modified using Maestro's built-in mutate residue tool, while PNA nucleobases were adjusted manually using default fragments provided by the software. Each modified triple helix was energy-minimized with MacroModel employing the OPLS4 force field and the Truncated Newton Conjugate Gradient (TNCG) method, achieving a convergence threshold of 0.05 kcal/mol/Å for the gradient.

The MD simulations utilized the OPLS4 force field. The system was solvated using the TIP3P water model within a cubic box extending 10 Å beyond the solute in all directions. Physiological conditions were mimicked by neutralizing the system with Na^+ ions and adding 0.15 M NaCl.

Relaxation involved five stages at 10 K: Brownian Dynamics NVT (100 ps), NVT (12 ps), NPT (12 ps), NPT with solute restraints (12 ps), and NPT without restraints (24 ps).

The production MD simulation was conducted in the NPT ensemble at 300 K and 1 atm for 50 ns, using a 2 fs timestep. The last 20 ns, representing the equilibrated phase, were used for analysis. Images were derived from Desmond trajectory clustering, performing trajectory RMSD analysis. In this work, we present images of the most abundant conformations identified through RMSD-based trajectory clustering of the equilibrated phase of the MD simulations.

References for SI

1. Wojciechowski, F.; Hudson, R. H. E. *J. Org. Chem.* **2008**, *73*, 3807-3816.

2. Holmes, B.; Lee, J.; Landon, K. A.; Benavides-Serrato, A.; Bashir, T.; Jung, M. E.; Lichtenstein, A.; Gera, J. *J. Biol. Chem.* **2016**, *291*, 14146-14159.

3. Accetta, A.; Corradini, R.; Sforza, S.; Tedeschi, T.; Brognara, E.; Borgatti, M.; Gambari, R.; Marchelli, R. *J. Med. Chem.* **2009**, *52*, 87-94.

4. Liu, Q.; Shi, W.-K.; Ren, S.-Z.; Ni, W.-W.; Li, W.-Y.; Chen, H.-M.; Liu, P.; Yuan, J.; He, X.-S.; Liu, J.-J.; Cao, P.; Yang, P.-Z.; Xiao, Z.-P.; Zhu, H.-L. *Eur. J. Med. Chem.* **2018**, *156*, 126-136.

5. Kumpina, I.; Baskevics, V.; Walby, G. D.; Tessier, B. R.; Saei, S. F.; Ryan, C. A.; MacKay, J. A.; Katkevics, M.; Rozners, E. *Synlett* **2024**, *35*, 649-653.

6. Brodyagin, N.; Hnedzko, D.; MacKay, J. A.; Rozners, E. Nucleobase-modified triplex-forming peptide nucleic acids for sequence-specific recognition of double-stranded RNA. In *Peptide Nucleic Acids. Methods and Protocols*; Nielsen, P. E., Ed.; Springer US: New York, 2020, pp. 157-172.

7. Brodyagin, N.; Maryniak, A. L.; Kumpina, I.; Talbott, J. M.; Katkevics, M.; Rozners, E.; MacKay, J. A. *Chem. - Eur. J.* **2021**, 4332-4335.

8. Ryan, C. A.; Baskevics, V.; Katkevics, M.; Rozners, E. *Chem. Commun. (Cambridge, U. K.)* **2022**, *58*, 7148-7151.
